# Densified HKUST-1 Monoliths as a Route to High Volumetric and Gravimetric Hydrogen Storage Capacity

**DOI:** 10.1021/jacs.2c04608

**Published:** 2022-07-25

**Authors:** David Gerard Madden, Daniel O’Nolan, Nakul Rampal, Robin Babu, Ceren Çamur, Ali N. Al Shakhs, Shi-Yuan Zhang, Graham A. Rance, Javier Perez, Nicola Pietro Maria Casati, Carlos Cuadrado-Collados, Denis O’Sullivan, Nicholas P. Rice, Thomas Gennett, Philip Parilla, Sarah Shulda, Katherine E. Hurst, Vitalie Stavila, Mark D. Allendorf, Joaquin Silvestre-Albero, Alexander C. Forse, Neil R. Champness, Karena W. Chapman, David Fairen-Jimenez

**Affiliations:** † The Adsorption & Advanced Materials Laboratory (A^2^ML), Department of Chemical Engineering & Biotechnology, University of Cambridge, Philippa Fawcett Drive, Cambridge CB3 0AS, U.K.; ‡ Department of Chemical Sciences and Bernal Institute, University of Limerick, Limerick V94 T9PX, Ireland; § Department of Chemistry, Stony Brook University, Stony Brook, New York 11790-3400, United States; ∥ Nanoscale and Microscale Research Center (nmRC), University of Nottingham, University Park, Nottingham NG7 2RD, U.K.; ⊥ School of Chemistry, University of Nottingham, University Park, Nottingham NG7 2RD, U.K.; # Synchrotron SOLEIL, Gif sur Yvette Cedex, Saint-Aubin 91190, France; ¶ 10 Laboratory for Synchrotron Radiation—Condensed Matter, Paul Scherrer Institute, PSI, 11, Villigen 5232, Switzerland; ∇ Laboratorio de Materiales Avanzados (LMA), Departamento de Química Inorgánica-IUMA, Universidad de Alicante, San Vicente del Raspeig 03690, Spain; ○ Immaterial Ltd., 25 Cambridge Science Park, Milton Road, Cambridge CB4 0FW, U.K.; ⧫ Materials and Chemical Science and Technology Directorate, National Renewable Energy Laboratory, Golden, Colorado 80401, United States; †† Chemistry, Combustion, and Materials Science Center, Sandia National Laboratories, Livermore, California 94551, United States; ‡‡ Yusuf Hamied Department of Chemistry, University of Cambridge, Cambridge CB2 1EW, U.K.; §§ School of Chemistry, University of Birmingham, Edgbaston, Birmingham B15 2TT, U.K.

## Abstract

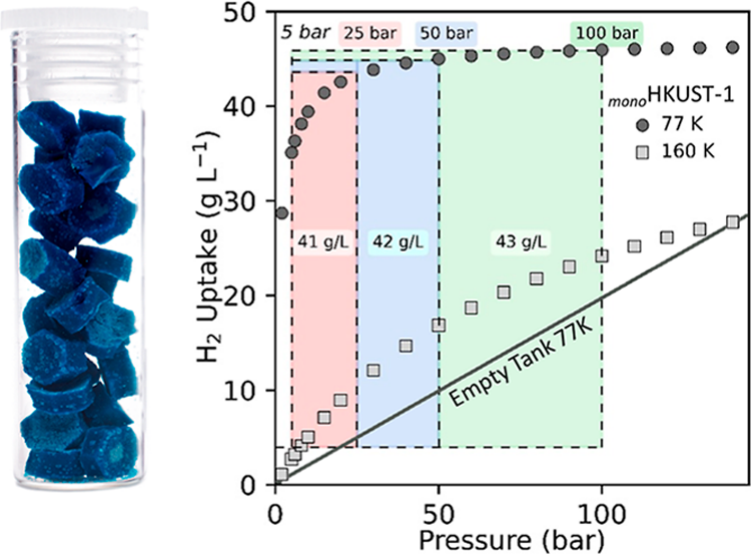

We are currently witnessing the dawn of hydrogen (H_2_) economy, where H_2_ will soon become a primary fuel for heating, transportation, and long-distance and long-term energy storage. Among diverse possibilities, H_2_ can be stored as a pressurized gas, a cryogenic liquid, or a solid fuel *via* adsorption onto porous materials. Metal–organic frameworks (MOFs) have emerged as adsorbent materials with the highest theoretical H_2_ storage densities on both a volumetric and gravimetric basis. However, a critical bottleneck for the use of H_2_ as a transportation fuel has been the lack of densification methods capable of shaping MOFs into practical formulations while maintaining their adsorptive performance. Here, we report a high-throughput screening and deep analysis of a database of MOFs to find optimal materials, followed by the synthesis, characterization, and performance evaluation of an optimal monolithic MOF (_mono_MOF) for H_2_ storage. After densification, this _mono_MOF stores 46 g L^–1^ H_2_ at 50 bar and 77 K and delivers 41 and 42 g L^–1^ H_2_ at operating pressures of 25 and 50 bar, respectively, when deployed in a combined temperature–pressure (25–50 bar/77 K → 5 bar/160 K) swing gas delivery system. This performance represents up to an 80% reduction in the operating pressure requirements for delivering H_2_ gas when compared with benchmark materials and an 83% reduction compared to compressed H_2_ gas. Our findings represent a substantial step forward in the application of high-density materials for volumetric H_2_ storage applications.

## Introduction

We are currently living in a time of great change as global transport transitions away from fossil fuels. As an alternative, H_2_ gas has long held great promise as a sustainable energy vector and an automotive transportation fuel as part of the H_2_ economy.^1−3^ H_2_ gas is a clean, potentially green, and non-toxic renewable fuel that contains much greater chemical energy per mass (142 MJ kg^–1^) when compared to hydrocarbon fuels. The combustion of H_2_ releases only water vapor as a by-product, allowing H_2_ fuel cell vehicles (FCV) to potentially provide zero-emission transportation. While containing *ca.* three times more energy per unit mass than gasoline, its onboard storage presents significant challenges. H_2_ is a very light gas and displays weak H_2_···H_2_ intermolecular forces, thus requiring cryogenic cooling and/or compression for storage at quantities (>5.6 kg) deemed sufficient for driving ranges (*ca.* 300 miles) comparable to traditional fuels.^4^

The US Department of Energy (DOE) set ambitious targets for FCV onboard H_2_ storage, requiring an initial system (including tank and materials) delivery capacity of 30 g L^–1^ (4.5 wt %) and an ultimate target of 50 g L^–1^ (6.5 wt %). FCVs utilizing compressed H_2_ gas (CHG) and cryo-compression methods have already been produced by major automobile manufacturers (BMW, Toyota, and Honda). However, these vehicles still require high gas operating pressures (>350 bar) and costly carbon fiber-reinforced storage tanks. Adsorbed gas storage (AGS) is considered a viable alternative to cryogenic or compressive storage, utilizing nanoporous materials to boost the hydrogen density in a tank at reduced operating pressures (*ca.* 100 bar). While traditional nanoporous materials such as activated carbons have been widely studied for H_2_ storage, these materials lack the versatility and structural tunability to be considered viable options for AGS technologies.^5−7^

**Figure 1:**
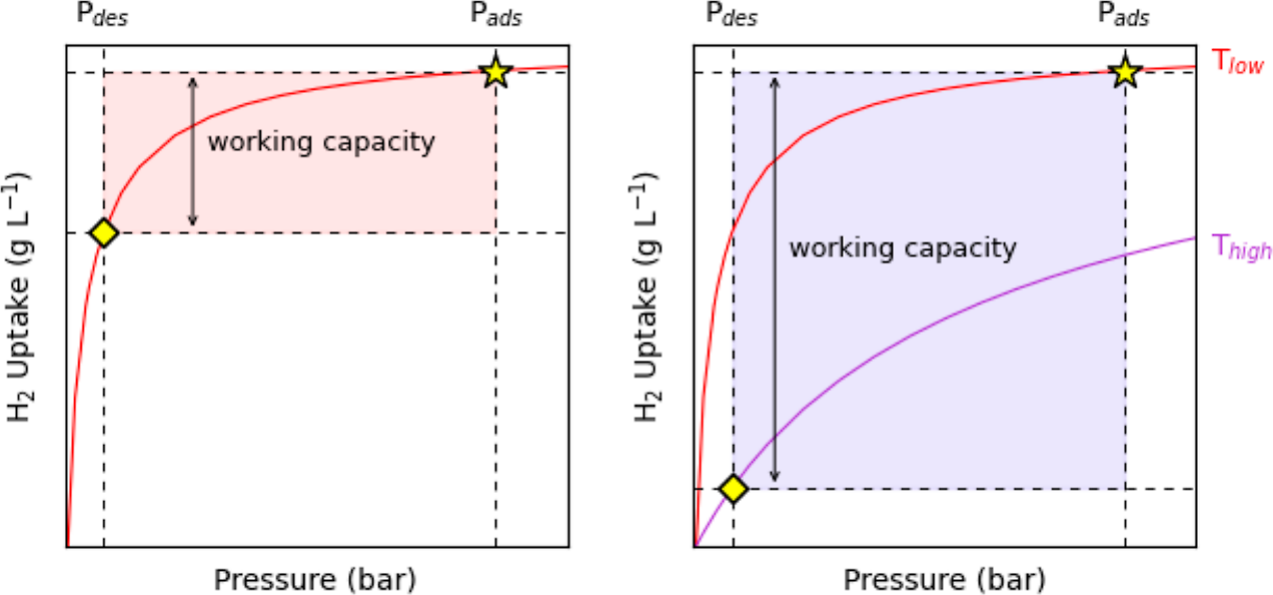
Idealized H_2_ adsorption isotherms for FCV storage systems. Illustration of usable volumetric capacity for (a) pressure swing and (b) temperature–pressure swing storage systems. Total volumetric adsorption isotherms are shown as purple and red curves, corresponding to high and low temperatures, respectively. The “charged” state of the tank is represented by a gold star, and the “discharged” state is represented by gold diamonds. Double-sided arrows represent volumetric usable capacities achieved for each system, with *P*_ads_ = 100 bar and *P*_des_ = 5 bar.

As an alternative, metal–organic frameworks (MOFs) are a class of nanoporous materials with a great potential for gas storage and separation applications. The tunability of this class of materials has given way to the synthesis of over 100,000 reported structures with a large array of interesting properties in terms of chemical and structural diversity.^8,9^ This versatility of MOFs has made them widely studied for AGS applications, including H_2_ and CH_4_. Several high-surface-area MOFs display benchmark performance with impressive gravimetric and volumetric H_2_ storage densities, both on the materials and system-based levels.^4^ Despite these advances, two major issues need to be addressed before MOFs can be deployed in FCVs. First, MOFs generally display type I isotherms for the adsorption of H_2_ under cryogenic conditions (Figure 1), with very high loadings at low pressures, followed by a saturation of the H_2_ uptake at higher pressures. This limits the overall working capacity of the adsorbent materials. To address this issue, the DOE Hydrogen Storage Engineering Center of Excellence (HSECoE) has proposed designing tanks for cryo-adsorption storage that operate with H_2_ loading occurring at 77 K and 100 bar and discharge occurring at 160 K and 5 bar, ensuring that the amount of deliverable H_2_ in nanoporous MOFs is maximized (Figure 1).^10^

The second, and arguably more important, issue hampering the deployment of MOFs for gas storage applications is the shaping and densification of MOF materials. While many MOFs display exceptional gravimetric H_2_ adsorption capacity, their performance does not readily translate to volumetric performance due to issues relating to MOF densification. MOFs are traditionally synthesized as powders with very low packing density that are formulated into shaped bodies *via* mechanical processes.^11−13^ These processes often yield low-density final products or materials with reduced performance as a result of the low pressures used in the processing or structural collapse when the pressures are high.^12,14^ Despite its importance, this is an area of research that has received relatively low attention, with many researchers choosing to report volumetric values based upon theoretical crystal densities as opposed to experimental bulk densities.^15^ While theoretical crystal densities play an important role in identifying candidate materials for H_2_ storage, the final packing densities of shaped materials can often be only a fraction of the theoretical crystal densities. Indeed, many MOFs suffer significant losses in porosity and overall adsorption performance upon densification due to pore collapse.^12,16,17^ As an alternative to the densification of bulk powders, control of particle size, morphology, and monodispersity before densification has recently shown potential for improving the packing densities for MOFs.^18^

In this work, we first used high-throughput computational screening and principal component analysis (PCA) to evaluate the landscape of the properties required to optimize hydrogen uptake in MOFs and to find an optimal structure, HKUST-1. Then, we used our recent developments in advanced sol–gel synthesis, engineering, and densification of MOFs to produce a pure monolithic HKUST-1 (_mono_HKUST-1) structure of up to about 1 cm^3^ in size without using high pressures or additional binders.^17,19−21^ We subsequently examined the unique nature of the local structures of the high-density _mono_HKUST-1 material using advanced characterization techniques such as synchrotron X-ray total scattering, mapping pair distribution function (PDF) studies, Raman microscopy, and solid-state nuclear magnetic resonance (NMR) spectroscopy studies. Finally, we examined the exceptional adsorption performance of _mono_HKUST-1 as the top-performing densified MOF for volumetric H_2_ storage. The performance of _mono_HKUST-1 suggests that advanced monolithic MOFs could pave the way for a new generation of high-performance, high-density adsorbents for both onboard vehicular AGS and stationary applications, dramatically reducing the pressure requirements for onboard H_2_ storage while improving both vehicle safety and driving distances in support of the H_2_ economy.

## Results and Discussion

### High-Throughput Computational Screening of MOFs

The exceptional tunability of MOFs has led to the experimental synthesis of thousands of MOFs and the prediction of millions.^8,22^ To evaluate the landscape of MOFs in hydrogen storage in this vast chemical space, we conducted high-throughput screening (HTS) studies by performing grand canonical Monte Carlo (GCMC) simulations on a database of 2,932 experimentally synthesized MOFs at four pressures of 5, 25, 50, and 100 bar and five temperature of 77, 160, 198, 233, and 298 K. We went one step further by performing a PCA on the vast amount of data generated in the HTS studies. We also highlighted 10 benchmark MOF materials for hydrogen storage in our screening—HKUST-1, MOF-5, NU-100/PCN-100, NU-1501-Al, NU-1500-Al, Ni(dobdc), MIL-101, IRMOF-10, UMCM-9, and IRMOF-20. Although some previous HTS studies have been reported in the literature, none have explored the range of conditions considered here.^23−25^

**Figure 2:**
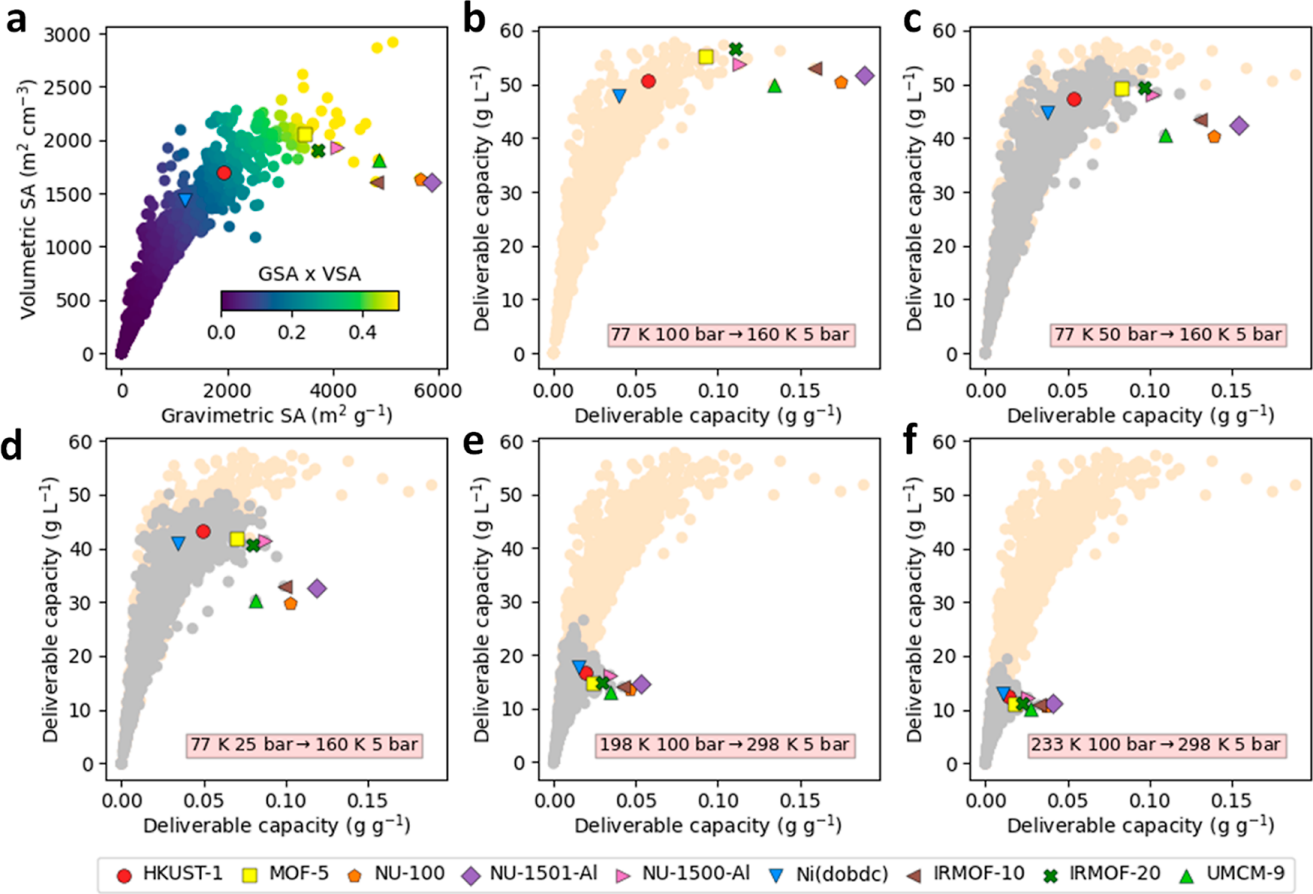
Computational screening of benchmark MOF materials. (a) Relationship between volumetric and gravimetric BET areas for 2940 MOFs. (b) Hydrogen volumetric and gravimetric deliverable capacities for a combined temperature–pressure H_2_ delivery system (100 bar/77 K → 5 bar/160 K). (c) Hydrogen volumetric and gravimetric deliverable capacities for a combined temperature–pressure H_2_ delivery system (50 bar/77 K → 5 bar/160 K). (d) Hydrogen volumetric and gravimetric deliverable capacities for a combined temperature–pressure H_2_ delivery system (25 bar/77 K → 5 bar/160 K). (e) Hydrogen volumetric and gravimetric deliverable capacities for a combined temperature–pressure H_2_ delivery system (100 bar/198 K → 5 bar/298 K). (f) Hydrogen volumetric and gravimetric deliverable capacities for a combined temperature–pressure H_2_ delivery system (100 bar/233 K → 5 bar/298 K). Peach-colored points in (b) to (f) represent H_2_ performance for a 100 bar/77 K → 5 bar/160 K system, while gray points represent H_2_ performance for the named system in (c) to (f).

Figure 2a shows the general landscape of the gravimetric and volumetric surface areas of the MOFs studied here. Benchmark MOF materials such as MOF-5, IRMOF-20, and NU-1500-Al displayed both exceptional gravimetric and volumetric surface areas. While materials such as NU-1501-Al and NU-100 displayed high gravimetric surface areas, the denser structure of HKUST-1 gave way to a higher volumetric surface area. On top of that, for hydrogen tank storage, an ideal MOF structure should not only have a high hydrogen storage capacity but, more importantly, should also possess a high deliverable capacity.^15^ To further probe the gas storage/adsorption performance, we determined the theoretical H_2_ deliverable capacities of the studied MOFs under five different combined temperature–pressure swing gas delivery systems, ranging from purely cryogenic (25, 50, and 100 bar/77 K → 5 bar/160 K) to near-ambient H_2_ delivery (100 bar/198 K and 100 bar/233 K → 5 bar/298 K). Figure 2b–f show the gravimetric and volumetric H_2_ deliverable capacities; the raw data are available in a dynamic visualization tool at: https://aam.ceb.cam.ac.uk/mofexplorer.html. Part 1 of the tool contains the data for purely cryogenic H_2_ delivery, whereas part 2 contains the data for near-ambient H_2_ delivery. Under cryogenic conditions and high pressure (100 bar/77 K), benchmark MOFs such as MOF-5, IRMOF-20, NU-1500-Al, IRMOF-10, and NU-1501-Al get the highest values in terms of both gravimetric and volumetric deliverable capacity (Figure 2b). Interestingly, as the storage pressure decreases (Figure 2c,d), denser MOFs with open-metal sites such as HKUST-1 and Ni(dobdc) begin to match and outperform large gravimetric surface area materials under volumetric conditions; the deliverable capacities of H_2_ for HKUST-1 display *ca.* 10% reduction when the storage pressure is reduced from 100 to 25 bar at 77 K. Under near-ambient conditions, HKUST-1 and Ni_2_(dobdc) outperformed all the other benchmark materials in terms of volumetric deliverable capacity. The exceptional performance of HKUST-1 and Ni_2_(dobdc) can be attributed to the denser crystal structure and high density of unsaturated metal centers, which give way to enhanced adsorbate–adsorbent interactions. The results of the HTS suggest that higher surface areas and larger pore volumes give way to exceptional H_2_ deliverable capacities at low temperatures and high pressures. Conversely, and as expected, denser structures and stronger adsorbent–adsorbate interactions give way to enhanced H_2_ deliverable capacities at lower pressures and higher temperatures.^12,23,26^

Once the HTS data have been collected, we moved to a PCA. Commonly used for dimensionality reduction, PCA helps to choose the minimum number of variables needed to explain the maximum amount of variance in the dataset. The raw data for the PCA are available in the dynamic PCA visualization tool at https://hydrogen-storage-pca.herokuapp.com. The Supporting Information (Figures S8–S10 and Table S7) provides more details about the geometric properties’ calculation, HTS studies, and PCA. From these studies, it is clear that optimizing the density of the material along with selecting an appropriate adsorption pressure for the process is crucial. Indeed, while it is clear that densification is key for the deployment of MOFs,^15^ it is also well known that the excess capacity reaches a maximum and then declines with increasing pressure because it becomes more efficient to pack molecules in the gas phase than on the surface.^27^

### Synthesis and Characterization

**Figure 3:**
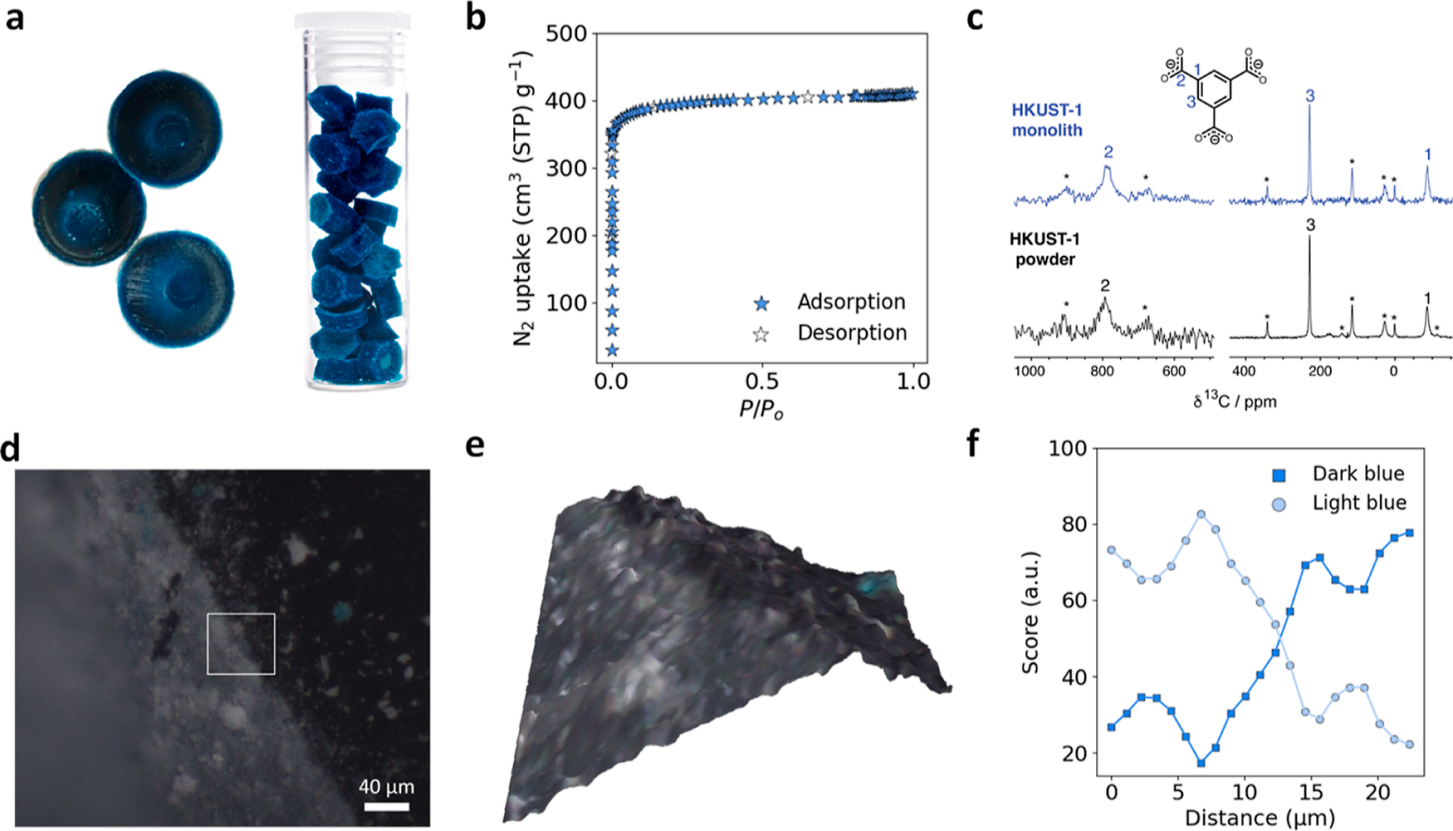
Characterization, NMR spectroscopy, and Raman microscopy studies of pristine _mono_HKUST-1. (a) Optical images of _mono_HKUST-1 prepared via traditional (Left) and scaled-up synthesis (Right). (b) Linear plot of the 77 K N_2_ adsorption isotherm for _mono_HKUST-1. (c) ^13^C NMR spectra of _mono_HKUST-1 and HKUST-1 powder samples. (d) Optical image and (e) three-dimensional reconstruction of the _mono_HKUST-1 section mapped by Raman microscopy, (f) showing the corresponding normalized CLS scores associated with Raman spectra of the dark blue and light blue portions over the mapped section.

Based on the HTS and PCA, we selected HKUST-1. Not only are its predicted volumetric absolute and deliverable capacities high but also looking at industrial production, it is based on a commercially available organic ligand and a simple synthesis process. In addition to standard synthetic methods, HKUST-1 can be made through spray-drying^28^ and mechanosynthesis.^29^ Here, we performed the synthesis of HKUST-1 not as a powder but as a high-density _mono_HKUST-1 using the previously reported sol–gel method.^17^ After the formation of the crystalline primary MOF particles at the beginning of the reaction, the mother solution was centrifuged, and the resulting MOF gel was washed to remove unreacted precursors. After three washing steps, the MOF gel was then allowed to dry overnight at room temperature, resulting in the formation of _mono_HKUST-1. Figure 3a displays an optical image of _mono_HKUST-1, while Figure S1 displays the powder X-ray diffraction (PXRD) patterns of the material. Once the _mono_HKUST-1 was dry, activation was carried out by heating to 120 °C under vacuum for 12 h. The _mono_HKUST-1 retains the macroscopic monolithic morphology and shape of the mold after activation. We obtained the envelope and particle packing densities of the monolithic and powdered materials, respectively, by mercury intrusion porosimetry (Figure S26). The measured envelope density of _mono_HKUST-1 is in agreement with the previously reported data, with an overall density of 1.07 g cm^–3^^17^ and verified by Particle Authority as a part of NREL H_2_ capacity characterization. We then evaluated the porosity using N_2_ adsorption at 77 K (Figures 3b, S2, and S3). Table S19 compares the densities, gravimetric and volumetric Brunauer, Emmett, and Teller (BET) areas calculated using Rouqueroĺs updated criteria implemented in BETSI (Figures S4 and S5),^30^ and pore volumes of _mono_HKUST-1 with those of powder and densified benchmark MOF materials. While _mono_HKUST-1 displays one of the lowest observed gravimetric BET areas (1552 m^2^ g^–1^) and total pore volume (0.634 cm^3^ g^–1^) of the materials presented, the critical advantage of the monolithic MOF is the high bulk density, which enables benchmark volumetric performance (BET area = 1,651 m^2^ cm^–3^; pore volume = 0.675 cm^3^ cm^–3^) which far exceeds that of powdered and mechanically pressed MOF counterparts (Table S19 and Figure S46).^12,16,31^ The measured bulk density of _mono_HKUST-1 (1.07 g cm^–3^) is higher than the crystal densities of HKUST-1 (0.883 g cm^–3^), which can be attributed to the presence of amorphous, denser phases within the monolithic material.^17^ Similar observations of high bulk density retention leading to high microporosity have been seen for previously studied _mono_ZIF-8 and _mono_UiO-66.^20,21^

Aiming to see if there are any structural or chemical differences between the powder and monolithic materials, we first examined the local environment of _mono_HKUST-1 by NMR spectroscopy. The ^13^C NMR spectra (Figure 3c) for _mono_HKUST-1 and HKUST-1 powder show similar peak assignments (Table S18) to those previously reported in the literature for HKUST-1,^32^ with no additional local environments observed for the BTC^3–^ linker (BTC^3–^ = 1,3,5-benzenetricarboxylate) in any sample. These results suggest, therefore, that the local chemical environment of the linker molecule in the powder and monolith materials is very similar at the bulk level. To further examine the local environment, we analyzed _mono_HKUST-1 by Raman microscopy. Raman spectra were initially collected by focusing on two independent regions of the monolithic sample corresponding to the lighter and darker blue sections, respectively, as seen by optical microscopy (Figures 3d and S33–S35); Figure S36 shows the Raman spectra for both sections. The dark blue regions display a spectrum that was found to be similar to previous reports on HKUST-1,^33^ whereas the lighter blue region contains additional peaks that can be attributed to BTC^3–^ hydrates and copper paddlewheel hydration. When the Raman mapping was performed (Figure 3e,f), by monitoring the normalized scores obtained from classic least squares (CLS) regression analysis and fitting the full spectra obtained from the dark and light blue regions of the _mono_HKUST-1 material, we can see a clear trend. Here, the spectrum associated with HKUST-1 becomes dominant as the Raman probe moves from the lighter blue to the darker blue section.

**Figure 4:**
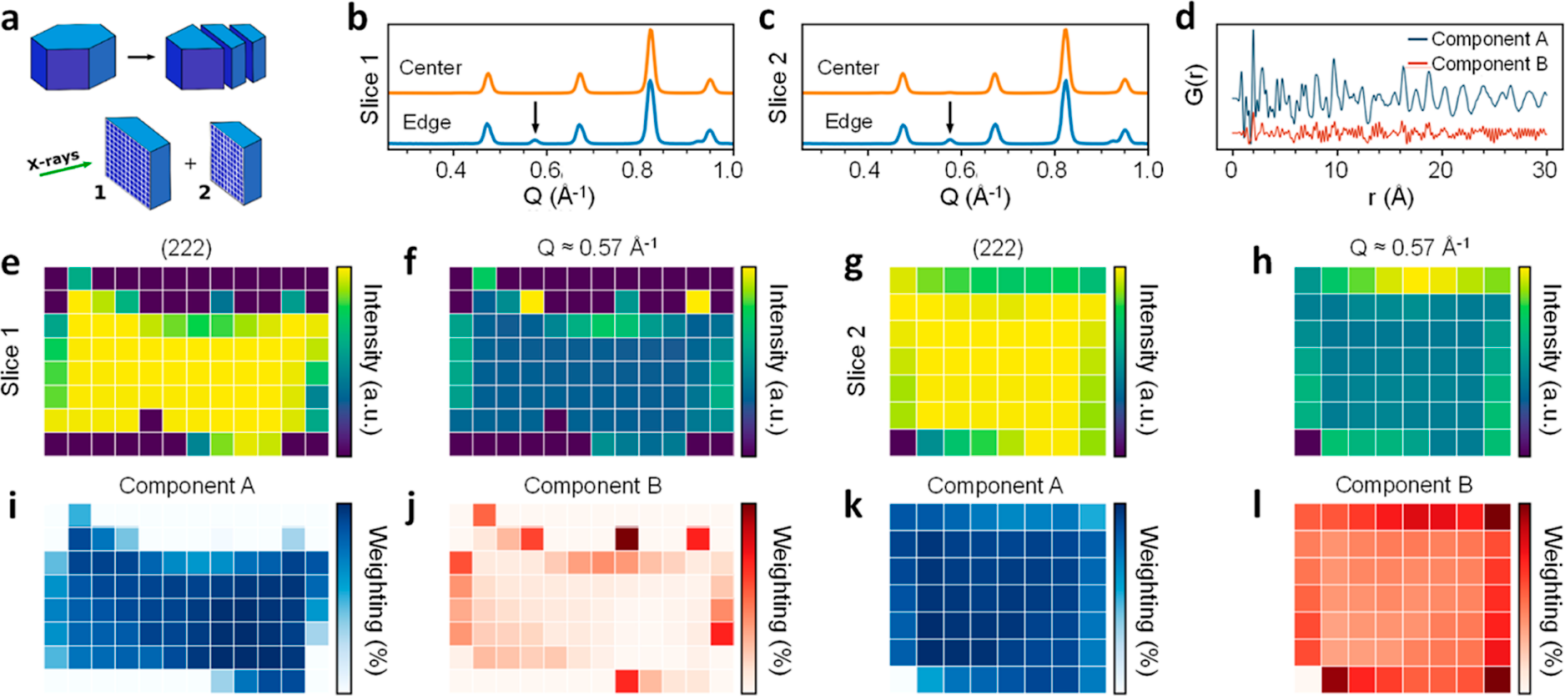
Mapping XRD and PDF studies for _mono_HKUST-1. (a) Monolithic samples were segmented into slices, and X-rays were used to map their cross-sections. (b,c) PXRD patterns collected at the edge (blue) and center (orange) of slices 1 and 2, respectively. Patterns collected at the edge exhibit spurious peaks at *Q* ≈ 0.57 Å^–1^ (noted with the black arrow) as well as at 0.93 Å^–1^. For comparison, the integral intensity of the (222) peak of HKUST-1 (*Q* ≈ 0.83 Å^–1^) is mapped for each slice (e,g, respectively) as well as the integral intensity of the peak at *Q* ≈ 0.57 Å^–1^ (f,h, respectively). (d) PDF components derived from NMF of all total scattering mapping data; the fractional weighting of components A (i,k) and B (j,l) are mapped to depict their distribution across the monolith.

We further probed the structural heterogeneity of _mono_HKUST-1 using synchrotron X-ray scattering experiments across multiple length scales by small-angle X-ray scattering (SAXS), PDF, and X-ray diffraction (XRD). We used SAXS (Figure S31) to determine the size of the primary MOF particles for both monolithic and powdered HKUST-1. Interestingly, while the _mono_HKUST-1 sample contains primary particles with a spherical diameter of *ca.* 20 nm (Figure S32), the powdered HKUST-1 sample was found to contain two broader distributions, with particles of *ca.* 24 to 92 nm in diameter. To evaluate the uniformity of the monolith, we sectioned _mono_HKUST-1 samples into *ca.* 1 mm segments (Figure S27) and mapped them in two dimensions with 500 μm^2^ resolution (Figure 4a). Diffraction patterns revealed differences in the scattering data collected from probe volumes at the external surface of the monolith (Figure 4b,c). To compare the relative presence of impurities, the normalized integral intensity of the spurious diffraction peak at *Q* = 0.83 Å^–1^ and the (222) peak (*Q* = 0.83 Å^–1^) of HKUST-1 was fitted using the cumulative trapezoid method as implemented in the Python package scipy.integrate (Figure 4e–h). To further probe the monolithic HKUST-1 PDF, we analyzed the data using previously described non-negative matrix factorization (NMF) techniques.^34^ Two components were used to describe the data (Figures 4d,i–l and S29). Comparison of these maps (Figure S30) reveals reasonable corroboration between the PDF-NMF components and the spurious diffraction peaks observed, with component A having a mean Pearson correlation of 0.92 with the (222) peak of slice 1 and 0.98 with the (222) peak of slice 2, and component B having a mean Pearson correlation of 0.78 with the spurious peak of slice 1 and 0.91 with the spurious peak of slice 2. Diffractograms collected in this region included additional peaks consistent with those previously ascribed to the hydrolytic decomposition of HKUST-1^35^ (Figure 4l). In contrast, the center of the monolith samples exhibited little to no presence of these peaks (Figure 4j) and fitted well with an HKUST-1 model without evidence of impurities. Indeed, this mapping matches with an observed difference in color between the center (dark blue) and edge (light blue) of the sample. Taking into account the above observations on Raman scattering, this further suggests the existence of HKUST-1 and a hydrated form, respectively.^33^ An analysis of the PDF data decomposed the data into two phases that correlate well with the distribution of HKUST-1 and the additional diffraction peaks. The PDF component corresponding to additional diffraction peaks exhibits limited radial distance atom–atom correlations with the exception of an increase in Cu–Cu distances, consistent with the hydration of the paddlewheel (Figure S30). It is remarkable that densification of the material in _mono_HKUST-1 not only improves volumetric adsorption but may also improve the hydrolytic stability of the material by limiting accessible surfaces to the outer edges of the monolith. The hydration of the Cu paddlewheels on the outer surfaces of _mono_HKUST-1 may act in a “sacrificial” manner similar to that observed for STAM-17-OEt, enabling the retention of the bulk porosity upon exposure to moisture.^36^ This was confirmed using 77 K N_2_ adsorption isotherms, which were performed on a _mono_HKUST-1 sample stored at room temperature for 18 months. This sample was found to retain over 90% of its overall BET area and porosity after 18 months of storage (Figure S48). The monolithic nature was found to significantly improve the chemical stability of the _mono_HKUST-1 material.

### Hydrogen Storage Performance

**Figure 5:**
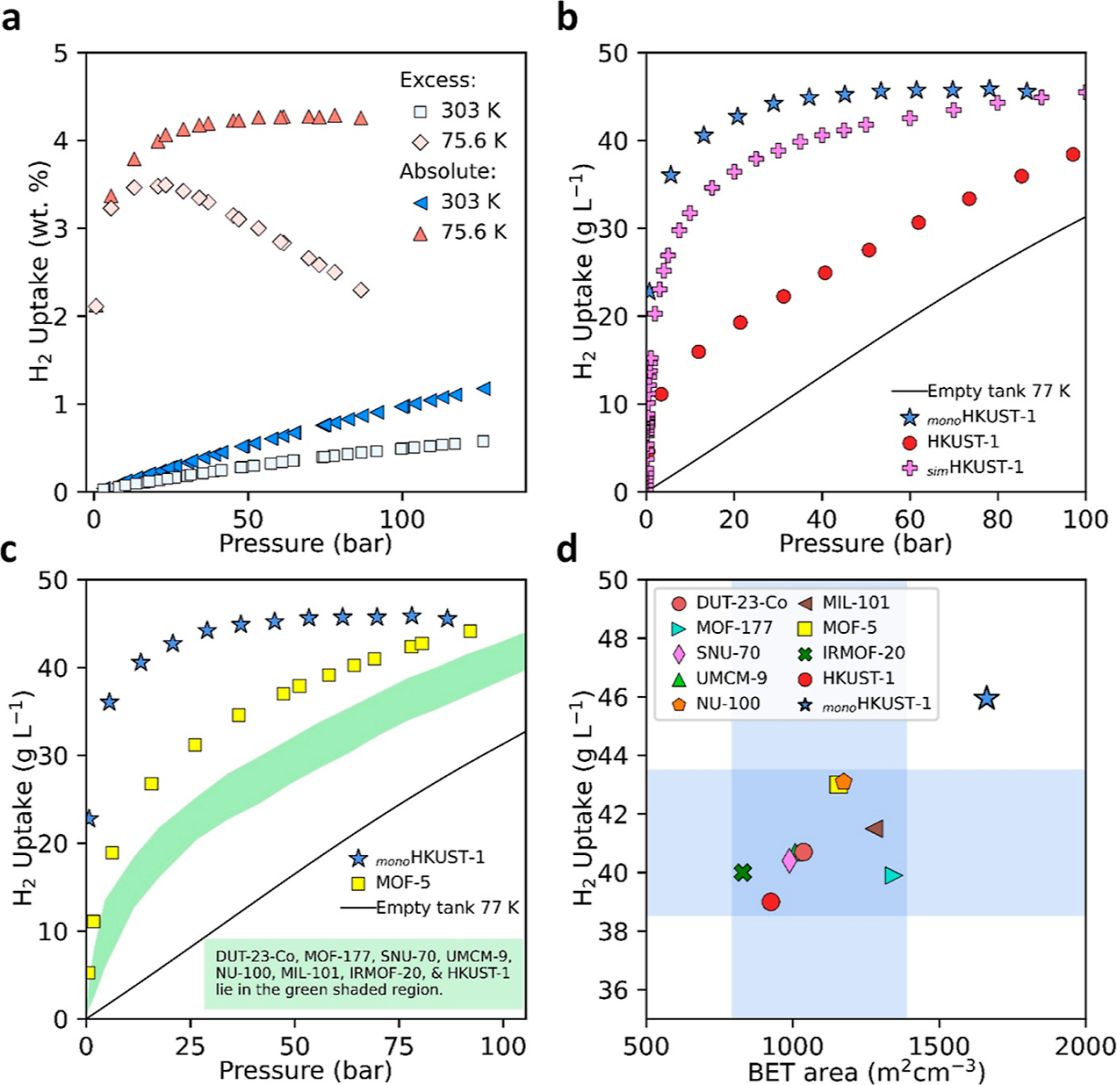
H_2_ adsorption isotherms of densified and monolithic MOF materials. (a) Excess and total (*N*_abs_) H_2_ adsorption isotherms for _mono_HKUST-1 measured at 75.6 K (liquid nitrogen measurement made at the National Renewable Energy Laboratory, elevation 5768 feet (1758 m)) and 303 K. An envelope density of 1.07 g mL^–1^ was used to calculate the volumetric H_2_ uptake of the _mono_HKUST-1 material. (b) High-pressure absolute (*N*_abs_) H_2_ isotherms of _mono_HKUST-1 compared to pressed HKUST-1 powder and simulated HKUST-1 uptake at 77 K. (c) 77 K H_2_ adsorption isotherms of _mono_HKUST-1 and previously reported densified MOFs.^12,41^ (d) 100 bar and 77 K H_2_ adsorption capacity vs volumetric BET area of _mono_HKUST-1 and previously reported benchmark densified MOF materials.^12,41^

To probe the improved performance of densified MOFs in H_2_ storage, we collected high-pressure adsorption isotherms at eight temperatures from 75.6 to 303 K and up to 140 bar (Figures 5 and S12–S15) on _mono_HKUST-1. To ensure reproducibility of the data, this was done in three separate laboratories: NREL, the University of Alicante, and the University of Cambridge. It is important to note that the experimentally measured values are excess amounts adsorbed (*N*_exc_), which were then transformed into absolute uptakes (*N*_abs_) by using eq 1 (Supporting Information, eq 5)
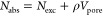
1where ρ is the density of the gas at the given adsorption pressure and temperature, obtained from the National Institute of Standards and Technology (NIST),^37^ and *V*_pore_ is the pore volume of the adsorbent.^27^ The calculated absolute adsorption (*N*_abs_) (Supporting Information, eq 5) based on excess (*N*_exc_) H_2_ isotherms collected at 75.6, 77, and 77 K (Figures 5a, S14, and S15) at NREL, the University of Cambridge, and the University of Alicante, respectively, were found to be in good agreement, displaying similar H_2_ uptakes at corresponding pressures for each of the three isotherms. Figures S12 and S13 display the NREL excess and calculate total (*N*_tot_) (Supporting Information, eq 6) H_2_ uptake at 75.6 and 303 K for comparison.^38−40^Figure 5b shows the absolute (*N*_abs_) volumetric adsorption isotherms of H_2_ at 75.6 K in _mono_HKUST-1 compared with a densified HKUST-1 powder and a simulated H_2_ isotherm for HKUST-1. The difference between isotherms is striking; interestingly, _mono_HKUST-1 displays higher H_2_ uptake at lower pressures compared to the densified powder sample, achieving a saturation uptake of *ca.* 46 g L^–1^ at 50 bar. In comparison, the densified HKUST-1 powder achieves an uptake of only *ca.* 28 g L^–1^ at 50 bar and of *ca.* 38 g L^–1^ at 100 bar. In comparison, the simulated absolute H_2_ uptake of HKUST-1 (*ca.* 45 g L^–1^) and _mono_HKUST-1 isotherms displays similar features. The higher uptake of _mono_HKUST-1 compared to the simulated isotherm can be attributed to the envelope density observed in _mono_HKUST-1 (1.07 g cm^–3^), which exceeds the theoretical crystal density for HKUST-1 (0.883 g cm^–3^).

Figure 5c compares the volumetric H_2_ adsorption performance of _mono_HKUST-1 with the performance of previously reported densified MOF materials (Figure S41) using real bulk densities of the materials;^12,13,31,41−44^Figure S40 compares the performance of _mono_HKUST-1 with the performance of previously reported benchmark MOF materials, with the caveat that this is done based on theoretical single-crystal density.^10,24,25,45,46^ Typically, a densified polycrystalline powder, as described above, will be many times limited to a density 50% lower than the theoretical crystal density.^12,16^ Importantly, a recent report has shown that the precise control of the MOF particle’s shape and size can give way to improved bulk densities in shaped MOF materials.^18^ Although the gravimetric H_2_ uptake of _mono_HKUST-1 is lower than that of all the previously reported materials studied herein (Table S19), the _mono_HKUST-1 material displays benchmark volumetric H_2_ adsorption performance. The closest material to _mono_HKUST-1 in terms of performance is MOF-5, displaying a H_2_ adsorption capacity of *ca.* 43 g L^–1^ at 100 bar. The performance of _mono_HKUST-1 was also found to outperform the benchmark carbon-based material AX21 at 100 bar and 77 K (Table S19).^47^ The H_2_ uptake performance of _mono_HKUST-1 at 25 and 50 bar exceeds the 100 bar uptake of the densified powder MOFs (Table S19, Figures 5c and S42). The exceptional performance of the _mono_HKUST-1 sample is attributed to the high bulk density achieved *via* a sol–gel synthesis approach, overcoming the lower densities and mechanical degradation issues associated with traditional powder pressing techniques.^12,16^ The effects of mechanical pressing of MOFs have a detrimental impact on the overall H_2_ adsorption performance for storage applications.^12,16^ For conformed, pressed polycrystalline powder materials, typical H_2_ excess adsorption capacities are generally retained up to a point where the density is increased up to *ca.* 50% of the single-crystal density (Figure S47). After that point, although the density continues to increase, the maximum excess adsorption value starts to decrease due to the continued mechanical collapse of the MOF.^12,16^ In contrast, the _mono_HKUST-1 sample was found to retain a high H_2_ adsorption capacity at bulk densities exceeding those of the crystal density of HKUST-1 (i.e., 1.07 vs 0.883 g cm^–3^). As has been seen elsewhere, the high-pressure H_2_ adsorption performance of the densified MOFs was found to follow a similar trend to that of the volumetric BET areas of the materials studied (Figure 5d).^12,25^ This means that volumetric BET area, using an appropriate density, is a valuable descriptor to predict the volumetric performance of MOFs.

**Figure 6:**
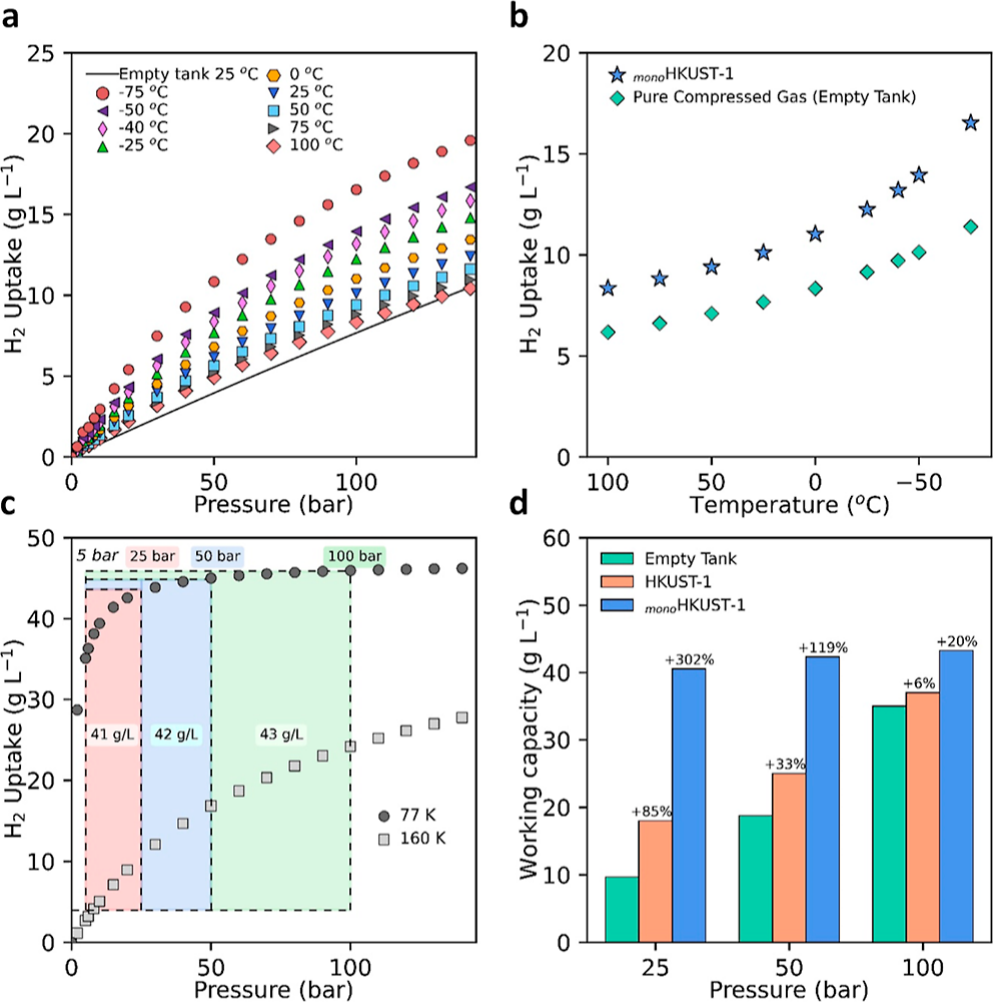
_mono_HKUST-1 H_2_ storage working capacities. (a) Near-ambient absolute H_2_ adsorption isotherms for _mono_HKUST-1 compared to compressed H_2_ gas at 25 °C. (b) Gas storage performance at 100 bar of _mono_HKUST-1 compared to compressed gas at near-ambient temperatures. (c) Cryogenic H_2_ gas delivery for the temperature–pressure swing (100 bar/77 K → 5 bar/160 K) storage system. (d) Deliverable H_2_ capacity of _mono_HKUST-1 compared to HKUST-1 powder and an empty tank at various adsorption pressures at 77 K.

To determine the adsorbate–adsorbent interaction energies for _mono_HKUST-1, we calculated the isosteric heats of adsorption (*Q*_st_) from H_2_ isotherms collected at eight temperatures using the Virial method (Figures S38 and S39). The experimental *Q*_st_ value for _mono_HKUST-1 was found to be in the range of 3.7–5.5 kJ mol^–1^. This value was found to be consistent with previously reported values for HKUST-1 in addition to other benchmark copper paddlewheel MOFs (NOTT-112 and NU-125).^25^*Q*_st_ is an important variable in understanding how easy it is to release the gases at lower pressures and/or higher temperatures. Indeed, the storage and release temperatures are another key factor for H_2_ storage materials. Since the current DOE targets only address hydrogen delivery temperature (−40 to 85 °C, to meet fuel cell system operation specifications) and not the storage system operating temperature, a range of possible system designs can be considered. To assess the performance of _mono_HKUST-1 over a wide range of temperatures, we applied the dual-process Langmuir (DPL)^48^ equation to the experimental isotherms (Figures S16–S25). We found the DPL equation to be in good agreement with the experimental data collected at eight temperatures. The initial conditions assessed for storage of H_2_ were near ambient (−75 to 100 °C) up to 100 bar (Figure 6a and Table S21). _mono_HKUST-1 displays a H_2_ adsorption capacity of 10.1 g L^–1^ at 25 °C and 100 bar, which is, to the best of our knowledge, the highest measured H_2_ capacity of a densified MOF—using real MOF density—under these conditions. However, the usable capacity in this case, with no temperature swing, is slightly reduced to 9.3 g L^–1^ due to the uptake of 0.84 g L^–1^ at 5 bar. This still outperforms compressed hydrogen, which would require compression to over 150 bar to obtain the same total volumetric usable capacity at 25 °C (Figure 6b). At 100 bar and a temperature of −75 °C, _mono_HKUST-1 takes up a total of 16.5 g L^–1^ H_2_, which corresponds to a total usable capacity of 14.8 g L^–1^. If the use of a temperature swing in a storage system is considered, through the application of active cooling at high filling levels, the usable capacities attained with _mono_HKUST-1 are even higher. For example, adsorption at −40 °C and desorption at 25 °C afford a usable capacity of 12.4 g L^–1^. A temperature swing from adsorption at −75 °C to desorption at 25 °C gives a usable capacity of 15.7 g L^–1^. This usable capacity represents the highest H_2_ volumetric usable capacity achieved to date for a densified adsorbent operating in this temperature range. Although these values are comparable to the current theoretical benchmarks (Ni_2_(dobdc), MOF-5, and V_2_Cl_2.8_(btdd))^10,49^ under these conditions (Table S21), it is important to highlight that these previous values are based on theoretical crystal densities and not experimental envelope densities, as reported here for _mono_HKUST-1. A natural assumption is to expect a *ca.* 50% reduction in the density and therefore in volumetric capacities in densified powders.^12,16^

When cryogenic conditions are employed for H_2_ storage, the use of a temperature swing step (i.e., 77 to 160 K) can increase the usable capacities by increasing the quantity of H_2_ desorbed upon cycling. While _mono_HKUST-1 displays an overall H_2_ uptake of 46.0 g L^–1^ at 100 bar and 77 K, the high H_2_ uptake at low pressures limits the working capacity to 11.2 g L^–1^. When a combined temperature–pressure swing system (100 bar/77 K → 5 bar/160 K) is employed, the working capacity increases to 43.3 g L^–1^ (Figure 6c). This exceeds the performance of all densified MOF benchmarks under similar conditions (Table S20).^12^ Although, a priori, this may sound contrary to the results obtained from the HTS, here it is important to point out that the force fields used in simulations tend to under-predict the H_2_ uptake for MOFs containing open-metal sites—including benchmark MOFs such as HKUST-1, NU-100, Ni(dobdc), and MIL-101 considered in this study—particularly at low pressures where polarization can play a significant role in H_2_ adsorption.^50^ Also, the structures used in silico are perfect single crystals and do not contain defects such as missing linkers, missing clusters, and so forth commonly seen in experimental structures. Combined, this can lead to differences between experimentally determined and theoretically delivered capacities. Interestingly, the _mono_HKUST-1 sample reaches >95% (43.8 g L^–1^) saturation at 25 bar, enabling H_2_ saturation at much lower pressures compared to many benchmark MOFs. When lower adsorption pressures are taken into account (Figure 6d), _mono_HKUST-1 exhibits working capacities of 42.3 and 40.5 g L^–1^, for loading pressures of 50 (50 bar/77 K → 5 bar/160 K) and 25 bar (25 bar/77 K → 5 bar/160 K), respectively. These values represent a 302 and 119% increase in the H_2_ volumetric storage capacities of an empty tank at 25 and 50 bar, respectively. By comparison, under ambient temperatures, H_2_ gas would need to be pressurized to 145 and 700 bar at 77 and 298 K, respectively, to achieve similar storage capacities. Again, to the best of our knowledge, this is the highest deliverable capacity achieved by any adsorbent after successful pelletization and shaping.

To design a material for adsorption applications, its volumetric capacity is not the only parameter that needs to be taken into consideration. Looking at the DOE targets, heat management due to the exo/endothermic nature of the adsorption/desorption phenomena, as well as efficient packing of a monolith in a tank, impurity tolerance (e.g., CO, H_2_O), recyclability (e.g., 100 cycles), and cost of adsorbent need to be optimized. In the case of _mono_HKUST-1, the three times higher density compared with that of the powder is expected to improve heat transfer significantly. Moreover, the generic approach of the sol–gel synthesis also allows for doping with materials such as activated carbon with higher thermal conductivity.^11^ In terms of cost, the primary limiting factors for _mono_HKUST-1 production include the starting materials’ cost, high solvent usage, and centrifuge cycling times.^51−53^ Solvent reduction and recovery combined can massively reduce _mono_HKUST-1 production costs (Figure S50). Additionally, by employing liquid-assisted grinding, it is possible to significantly reduce mixing times by using prepared nanocrystalline powders to form high-density _mono_HKUST-1 materials while maintaining monolith quality (Figure S49). Predictably, yield is also a large cost driver, and any cost-effective production will seek to maximize yield. The simplicity of the synthesis of monolithic MOFs combined with their exceptional performance indicates that monolithic MOFs could play an important role in fuel gas storage in the coming decades.

## Conclusions

In conclusion, we have investigated computationally the landscape of MOFs for H_2_ storage and selected HKUST-1 as the optimal structure due to its adsorption capacity and ease of synthesis. Following this, we synthesized and analyzed the structure and H_2_ adsorption properties of the monolithic version, _mono_HKUST-1. SAXS, NMR spectroscopy, and Raman microscopy studies demonstrated that _mono_HKUST-1 exhibits similar characteristics in terms of composition and connectivity to powdered HKUST-1. However, the small and uniform primary particles result in exceptionally close packing, giving way to high-density final materials when mild drying conditions are applied. The monolithic structure of _mono_HKUST-1 also gives way to reduced material degradation, a common issue with HKUST-1 powders. The high-density structure forms an oxidized layer on the external surface which reduces the exposure of HKUST-1 particles within the monolith to moisture, maintaining exceptional performance after prolonged periods of exposure to the atmosphere. The exceptional high-density structure of _mono_HKUST-1 gives way to record-breaking H_2_ storage performance. The unique synthesis mechanism for _mono_HKUST-1 enables the formation of materials that maintain porosity after shaping and display benchmark volumetric BET areas, which in turn gives way to exceptional H_2_ sorption performance. The _mono_HKUST-1 materials were found to be capable of achieving H_2_ working capacities at 25 bar under cryogenic conditions, which was only possible by compressing H_2_ to 700 bar at room temperature. This reduction in operating pressures has the potential to significantly reduce the systemwide engineering requirements and cost while simultaneously improving the overall safety of onboard H_2_ storage for vehicular transport. While further development is required to identify more stable materials with high working capacities, this work represents a significant step forward in the shaping and densification of MOFs for H_2_ storage applications.

## Supporting Information Available

Additional experimental procedures, in situ procedures and results, and computational information (PDF)
Full details of previous high-throughput screening (HTS) studies of MOFs for H_2_ storage (XLSX)
