# Supplementary material for: Densified HKUST-1 Monoliths as a Route to High Volumetric and Gravimetric Hydrogen Storage Capacity
Source: J Am Chem Soc. 2022 Jul 25;144(30):13729–39. doi: 10.1021/jacs.2c04608 (PMC9354247; doi:10.1021/jacs.2c04608)
Supplement: Supplementary file 1 — ja2c04608_si_001.pdf [file ja2c04608_si_001.pdf]

Supporting Information for:

Densified HKUST-1 Monoliths as a Route to High Volumetric and Gravimetric Hydrogen Storage Capacity

David Gerard Madden[a]†*, Daniel O'Nolan[c]†, Nakul Rampal[a]†, Robin Babu[a], Ceren Çamur[a], Ali N. Al Shakhs[a], Shi-Yuan Zhang[a], Graham A. Rance[d,e], Javier Perez[f], Nicola Pietro Maria Casati[g], Carlos Cuadrado-Collados[h], Denis O'Sullivan[i], Nicholas P. Rice[i], Thomas Gennett[j], Philip Parilla[j], Sarah Shulda[j], Katherine E. Hurst[j], Vitalie Stavila[k], Mark D. Allendorf[k], Joaquin Silvestre-Albero[h], Alexander C. Forse[l], Neil R. Champness[m], Karena W. Chapman[c]* and David Fairen-Jimenez[a]*

[a]*The Adsorption & Advanced Materials Laboratory (A[2]ML), Department of Chemical Engineering & Biotechnology, University of Cambridge, Philippa Fawcett Drive, Cambridge CB3 0AS, UK.*

[b]*Department of Chemical Sciences and Bernal Institute, University of Limerick, Limerick V94 T9PX, Ireland.*

[c]*Department of Chemistry, Stony Brook University, Stony Brook, New York 11790-3400, United States*

[d]*Nanoscale and Microscale Research Centre (nmRC), University of Nottingham, University Park, Nottingham, NG7 2RD.*

[e]*School of Chemistry, University of Nottingham, University Park, Nottingham, NG7 2RD.*

[f]*Synchrotron SOLEIL, Gif sur Yvette Cedex, France*

[g]*10 Laboratory for Synchrotron Radiation—Condensed Matter, Paul Scherrer Institute, 5232 Villigen–PSI, 11 Switzerland.*

[h]*Laboratorio de Materiales Avanzados (LMA), Departamento de Química Inorgánica-IUMA, Universidad de Alicante, 03690 San Vicente del Raspeig, Spain*

[i]*Immaterial Ltd., 25 Cambridge Science Park, Milton Road, Cambridge CB4 0FW, UK*

[j]*Materials and Chemical Science and Technology Directorate, National Renewable Energy Laboratory, Golden, Colorado 80401, United States*

[k]*Chemistry, Combustion, and Materials Science Center, Sandia National Laboratories, Livermore, California 94551, United States*

[l]*Yusuf Hamied Department of Chemistry, University of Cambridge, Cambridge CB2 1EW, UK*

[m]*School of Chemistry, University of Birmingham, Edgbaston, Birmingham, B15 2TT, UK*

[*]*E-mail: dm850@cam.ac.uk, karena.chapman@stonybrook.edu, df334@cam.ac.uk*

†These authors contributed equally

Table of Contents

Materials and Methods....S3

Materials....S3

Synthesis of HKUST-1 samples S3

Powder X-ray diffraction (PXRD)....S3

Gas adorption measurements. S4

Molecular Simulations S7

Principal Component Analysis (PCA) S15

NREL high-pressure gas adsorption measurements S19

University of Cambridge/University of Alicante gas adsorption studies S25

Dual-Process Langmuir Model S30

Mercury porosimetry density measurement....S36

Solid State Nuclear Magnetic Resonance (NMR) S37

X-ray Total Scattering Studies....S37

Small Angle X-Ray Scattering (SAXS) Studies....S41

Raman microscopy experimental....S43

Isosteric heat of adsorption (Q_{st}) calculations. S46

High Pressure H_2 Adsorption Comparisons....S48

Monolith Stability Testing S59

Synthesis Scale-Up Cost Reductions....S60

References....S62

Materials and Methods

Materials

$Cu(NO_3)_2 \cdot 2.5H_2O$ (98%), 1,3,5-benzenetricarboxylic acid (BTC) (95%) and ethanol (≥99.5%) were purchased from Sigma Aldrich and used as received.

Synthesis of HKUST-1 samples

$_{mono}$HKUST-1 was synthesized *via* the previously reported method [1]. Solutions of BTC (10 ml, 0.062 M) and $Cu(NO_3)_2 \cdot 2.5H_2O$ (10 ml, 0.064 M) in ethanol were mixed and stirred for 10 min at room temperature (20 ± 1 °C). After centrifugation, the solid was kept in the Falcon tube and washed in ethanol for 10 min (15 ml, 3 times) and then dried in an incubator at room temperature (20 ± 1 °C) overnight. The solid was then transferred to a glass vial and was further dried at 120 °C in an incubator under vacuum overnight. Powdered HKUST-1 was obtained by drying the washed solid after centrifugation, at high temperature (120 °C) rather than allowing them to dry first at room temperature. All samples were activated at 180 °C overnight under vacuum.

Powder X-ray diffraction (PXRD) patterns (**Fig. S1**) were recorded with a Bruker D8 diffractometer at 40 kV and 40 mA using $CuK_{\alpha 1}$ (λ = 1.5405 Å) radiation with a step of 0.02° at a scanning speed of 8 s per step. Monolith powders were prepared for PXRD analysis by gently crushing with a pestle and mortar before being placed on a zero-background silicon wafer.

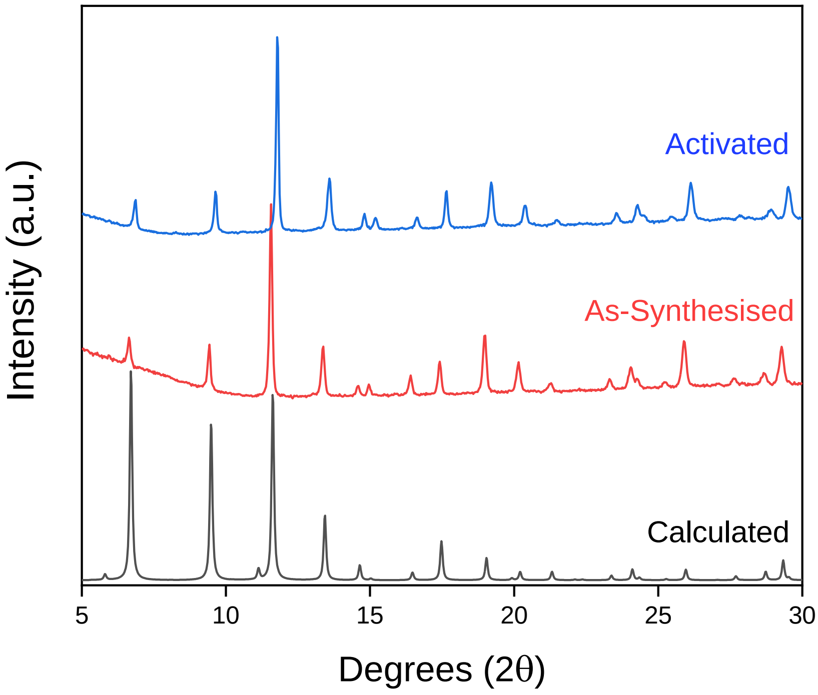

Figure S1. Powder X-ray diffraction patterns (PXRD) for as-synthesised and activated $_{mono}$HKUST-1 compared to calculated PXRD.

Gas adorption measurements. Ultra-high-purity grade N_2 was used for gas sorption experiments. Adsorption experiments (up to 1 bar) for different pure gases were performed on Micromeritics 3Flex surface area and pore size analyzer (**Fig. S2-S3**). About 200 mg of activated samples were used for the measurements. Liquid nitrogen was used to maintain constant temperature in the bath through the duration of the experiment. Samples were degassed on a Smart VacPrep prior to the analysis.

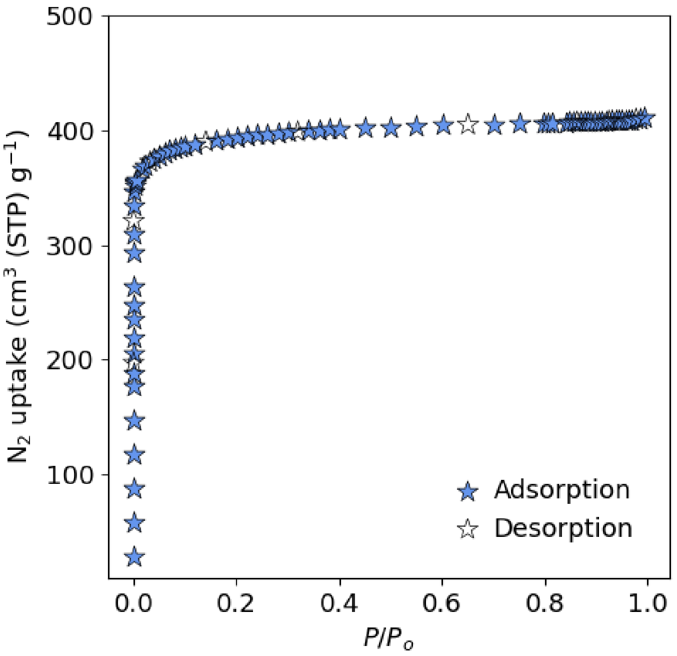

Figure S2. Linear 77 K N_2 adsorption isotherm for $_{mono}$HKUST-1. Closed stars represent adsorption whilst open stars represent desorption.

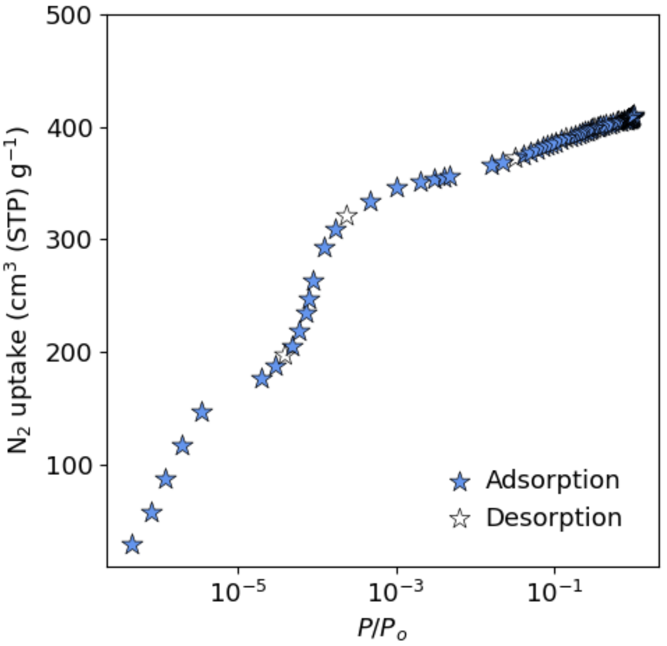

Figure S3. Logarithmic 77 K N_2 adsorption isotherm for $_{mono}$HKUST-1. Closed stars represent adsorption whilst open stars represent desorption.

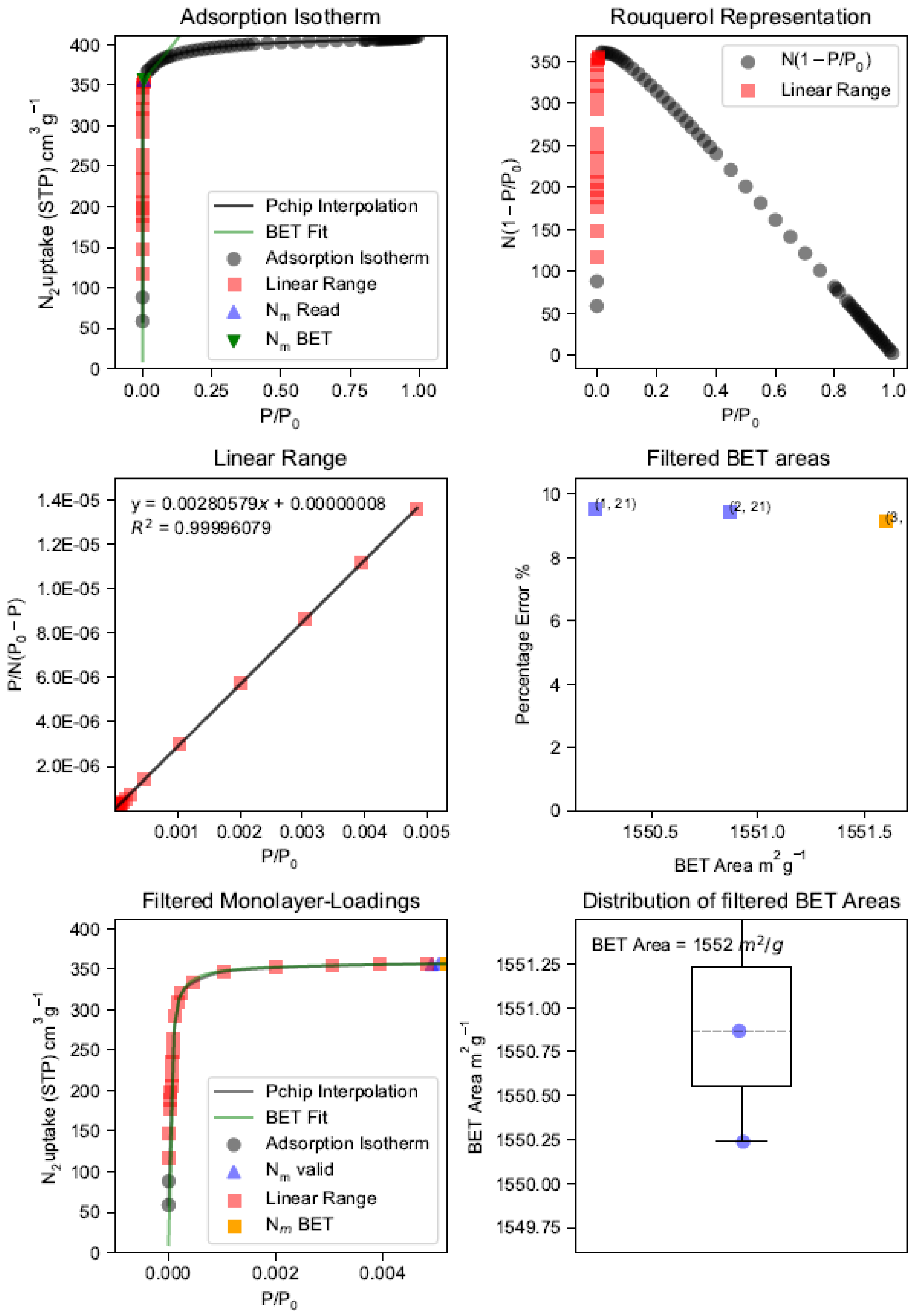

Figure S4. BETSI fitting and BET area calculations for ${}_{mono}$HKUST-1.

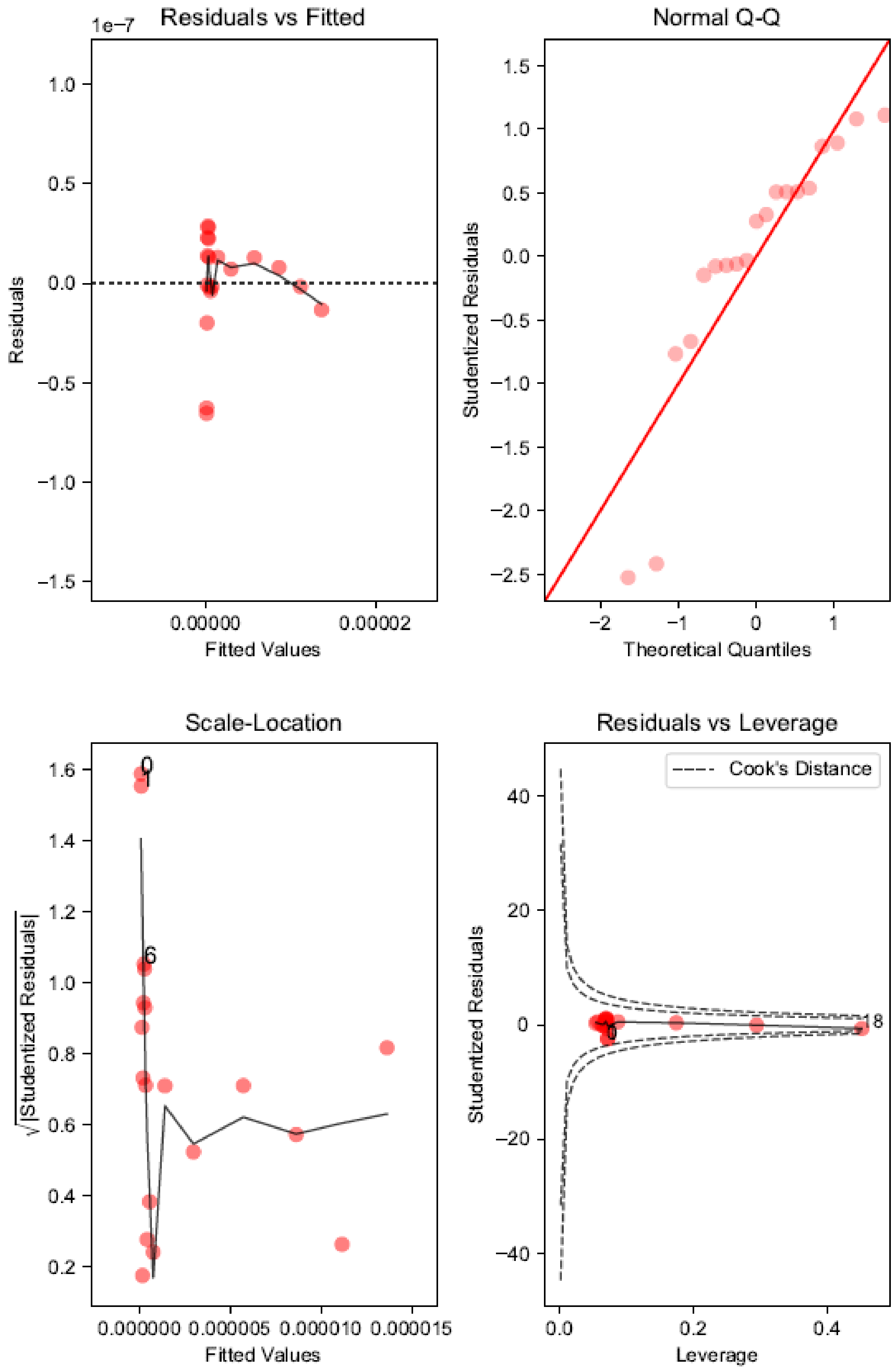

Figure S5. Regression analysis for BETSI fitting and BET area calculations for $_{mono}$HKUST-1.

Molecular Simulations

The crystallographic information files (cif) of the 2932 MOFs studied in this work are taken from the DDEC database [2]. We also included 10 MOFs with benchmark performance for H_2 storage in our high-throughput screening study – HKUST-1, MOF-5, NU-100/PCN-610, NU-1501-Al, NU-1500-Al, Ni(dobdc), MIL-101, IRMOF-10, UMCM-9, and IRMOF-20. More details about the cif of these benchmark materials are given in **Table S1**. The low pressure adsorption of N_2 and absolute adsorption capacity for H_2 at 77 K is simulated using the Grand Canonical Monte Carlo method as implemented in the RASPA simulation package [3]. Monte Carlo moves consist of insertion, deletion, rotation and translation moves, each with equal probabilities. For all the pressure points we use 20,000 cycles for equilibration and another 10,000 cycles to average the properties. A cycle is defined as the maximum of 20 steps or the number of molecules in the system. This implies that on an average a Monte Carlo move has been attempted on all the molecules during each cycle. Intermolecular interactions are modeled using the Lennard-Jones (LJ) potential (1) with a cutoff of 12.8 Å:

$$V_{ij}^{LJ}(r) = 4\varepsilon_{ij}\left(\left(\frac{\sigma_{ij}}{r}\right)^{12} - \left(\frac{\sigma_{ij}}{r}\right)^{6}\right) \tag{1}$$

where ε_{ij} is the depth of the potential well, σ_{ij} is the distance at which the intermolecular potential between the two particles is zero, and r_{ij} is the distance between atom i and atom j. Lorentz Berthelot mixing rules are used for all the cross-interaction terms:

$$\varepsilon_{ij} = \sqrt{\varepsilon_i \varepsilon_j} \text{ and } \sigma_{ij} = \sqrt{\sigma_i \sigma_j} \tag{2}$$

where ε and σ are the LJ parameters (as described above) and the subscripts indicate atom i and atom j. Electrostatic interactions are modeled using the coulombic potential (3) and are computed using the Ewald summation method with the precision set to 10^{-6}:

$$\boldsymbol{V_{ij}^{electrostatic}} = \boldsymbol{\frac{q_i q_j}{4\pi\epsilon_0 r_{ij}}} \tag{3}$$

where, q is the charge, ϵ_0 is the permittivity, and r_{ij} is as defined above. N_2 is modeled using parameters taken from the TraPPE force field [4]. **Table S2** shows the parameters used for N_2 in this study. H_2 is modeled as a rigid three-site model with parameters taken from the work of Bucior et al. [5]. **Table S3** shows the parameters used for H_2 in this study. To account for the quantum effects, which are important at cryogenic temperatures [6], the LJ interactions between H_2-H_2 and H_2-framework are modified to include the Feynman-Hibbs correction [7] (4):

$$V_{ij}^{feynman-hibbs} = V_{ij}^{LJ}(r) + \frac{\hbar^2}{24\mu k_B T}\nabla^2 V_{ij}^{LJ}(r) \tag{4}$$

where $\hbar$ is the planck's constant, k_B is the Boltzmann constant, $\mu = m_i m_j/(m_i + m_j)$ is the reduced mass, and T is the temperature. The framework atoms are modeled using LJ parameters taken from both the DREIDING force field [8] and the Universal force field [9]. **Table S4** shows the LJ parameters used to describe the atoms in the framework. The framework is modeled as a rigid structure with periodic boundary conditions applied in all directions. The number of unit cells in the simulation box is computed individually for each of the frameworks to make sure the simulation box is large enough to ensure that a distance of at least twice the cutoff radius is maintained between the periodic images. For structures not present in the DDEC database, the partial charges of the framework atoms are calculated using the EQeq protocol [10]. The geometric properties are calculated using Poreblazer [11].

Table S1. Details about the crystallographic information files (cif) for the 10 benchmark MOF materials. Column 1 lists the name of the material and column 2 lists the remarks for these materials.

Material	Remarks
HKUST-1	Taken from the DDEC database, CSD Refcode: FIQCEN
MOF-5	Taken from the DDEC database, CSD Refcode: MIBQAR
NU-100/PCN-610	Taken from the CSD, Refcode: HABQUY
NU-1501-Al	Taken from the CSD, Refcode: HUHZAO, disorder was corrected
NU-1500-Al	Created in Material Studio using the CIF file of NU-1500-Fe received from the lab of Prof. Omar Farha [12]
Ni(dobdc)	Taken from the CSD, Refcode: ORIVUI
MIL-101	Taken from the CSD, Refcode: OCUNAC, solvent molecules removed and missing hydrogens added
IRMOF-10	Taken from RASPA [3]
UMCM-9	Received from the lab of Prof. Donald Siegel [13]
IRMOF-20	Taken from the CSD, Refcode: VEGBHUG, disorder was corrected

Table S2. Lennard Jones parameters and charges for N_2. Column 1 lists the name of the adsorbate; column 2 lists the corresponding atom types of N_2, where M is the dummy atom; columns 3, 4 and 5 list their corresponding LJ parameters and charges, σ in Å, ε/K_b in K, and q in e, taken from the TraPPE force field.

Adsorbate	Atom	LJ Parameters		**q (e)**
		**σ (Å)**	**ε/k$_B$ (K)**	
	N	3.31	36	-0.482
N_2	M	0	0	0.964
	N	3.31	36	-0.482

Table S3. Lennard Jones parameters and charges for H_2. Column 1 lists the name of the adsorbate; column 2 lists the corresponding atom types of H_2, where COM is the center of mass site; columns 3, 4 and 5 list their corresponding LJ parameters (taken from the Michels-Degraff-Tenseldam model [14]) and charges (taken from the Darkrim-Levesque model [15]), σ in Å, ε/K_b in K, and q in e.

Adsorbate	Atom	LJ Parameters		**q (e)**
		**σ (Å)**	**ε/k$_B$ (K)**	
	H	0	0	0.468
H_2	COM	2.958	36.7	-0.936
	H	0	0	0.468

Table S4. Lennard Jones parameters, and mass for the atoms in the framework. Column 1 lists the atom type; columns 2 and 3 list the Lennard Jones parameters for the corresponding atom types, σ in Å and ε/K_b in K, taken from the DREIDING force field (marked with an asterisk) and from the UFF; Column 4 lists the mass of the atom in amu.

Atom	LJ Parameters		Mass (amu)	Atom	LJ Parameters		Mass (amu)
	**σ (Å)**	**ε/k$_B$ (K)**			**σ (Å)**	**ε/k$_B$ (K)**	
C*	3.473	47.888	12.0107	Al*	3.911	156.102	26.98154
H*	2.846	7.654	1.00794	Si*	3.804	156.102	28.0855
N*	3.263	38.975	14.00674	Ca	3.028	119.846	40.08
O*	3.033	48.190	15.9994	Sc	2.936	9.568	44.9559
P*	3.697	161.138	30.97376	Ti*	4.045	27.696	47.9
F*	3.093	36.508	18.9984	V	2.801	8.057	50.9415
Cl*	3.519	142.658	35.453	Cr	2.693	7.553	51.996
K	3.396	17.624	39.0983	Ga*	3.911	201.423	69.72
Co	2.559	7.050	58.9332	Ge*	3.804	201.423	72.59
Ni	2.525	7.553	58.6934	As*	3.697	206.458	74.9216
Mg	2.691	55.895	24.305	Se*	3.590	216.529	78.96
Mn	2.638	6.546	54.93804	Br*	3.519	186.316	79.904
Fe	2.594	6.546	55.845	Rb	3.665	20.142	85.4678
Zn	2.462	62.441	65.38	Sr	3.244	118.336	87.62
Cu	3.114	2.518	63.546	Y	2.980	36.256	88.9059
S*	3.590	173.223	32.065	Nb	2.820	29.710	92.9064
Zr	2.783	34.745	91.224	Mo	2.719	28.199	95.94
Li	2.184	12.589	6.9411	Ru*	4.045	27.696	101.07
Be	2.446	42.802	9.012182	Rh	2.609	26.688	102.9055
B*	3.581	47.838	10.811	Pd	2.583	24.171	106.4
Na	2.658	15.107	22.98977	Ag	2.805	18.128	107.868

Cd	2.537	114.811	112.41
In*	4.089	276.956	114.82
Sn*	3.982	276.956	118.69
Sb*	3.875	276.956	121.75
Te*	3.769	287.027	127.6
I*	3.697	256.814	126.9045
Cs	4.024	22.660	132.9054
Ba	3.299	183.295	137.33
La	3.138	8.560	138.9055
Ce	3.168	6.546	140.12
Pr	3.213	5.036	140.9077
Nd	3.185	5.036	144.24
Sm	3.136	4.028	150.4
Eu	3.112	4.028	151.96
Gd	3.001	4.532	157.25
Tb	3.074	3.525	158.9254
Dy	3.054	3.525	162.5
Ho	3.037	3.525	164.9304
Er	3.021	3.525	167.26
Tm	3.006	3.021	168.9342
Yb	2.989	114.811	173.04
Lu	3.243	20.646	174.967
Hf	2.798	36.256	178.49
Ta	2.824	40.788	180.9479
W	2.734	33.738	183.85
Re	2.632	33.235	186.207
Os	2.780	18.632	190.2
Ir	2.530	36.760	192.22
Pt	2.454	40.285	195.09
Au	2.934	19.639	196.9665
Hg	2.410	193.869	200.59
Pb	3.828	333.858	207.2
Bi	3.893	260.842	208.9804
Th	3.025	13.092	204.37
U	3.025	11.078	238.029
Np	3.050	9.568	237.0482
Pu	3.050	8.057	242
Am	3.012	7.050	243

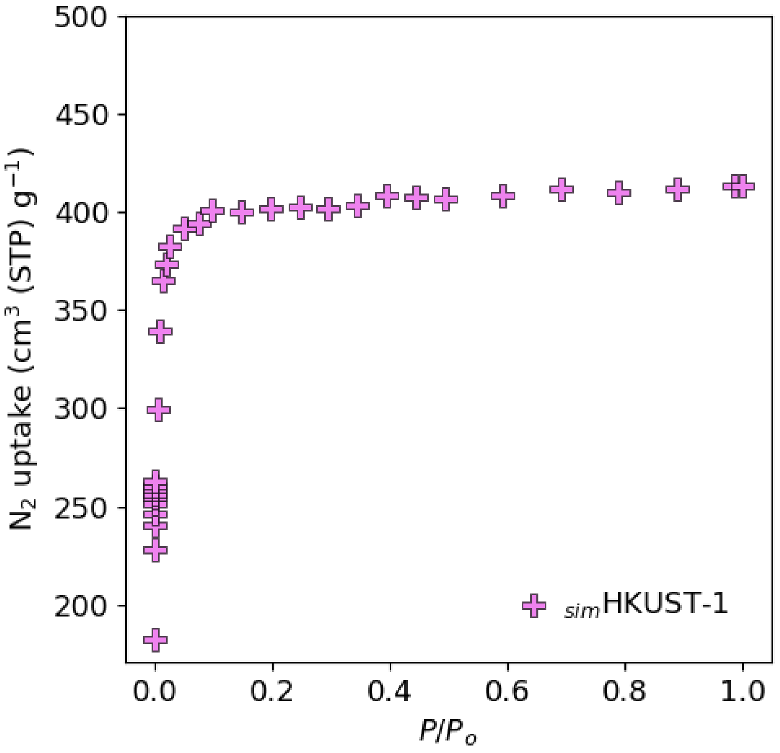

Figure S6. Simulated 77 K N_2 adsorption isotherm for $_{mono}$HKUST-1. Simulations based on crystallographic information file (cif) for HKUST-1. Scaling factor applied based on the properties of $_{mono}$HKUST-1 (Pore Volume (V_{pore}) = 0.634 cm^3 g^{-1}; Envelope density ($\rho_{envelope}$) = 1.064 g cm^{-3})

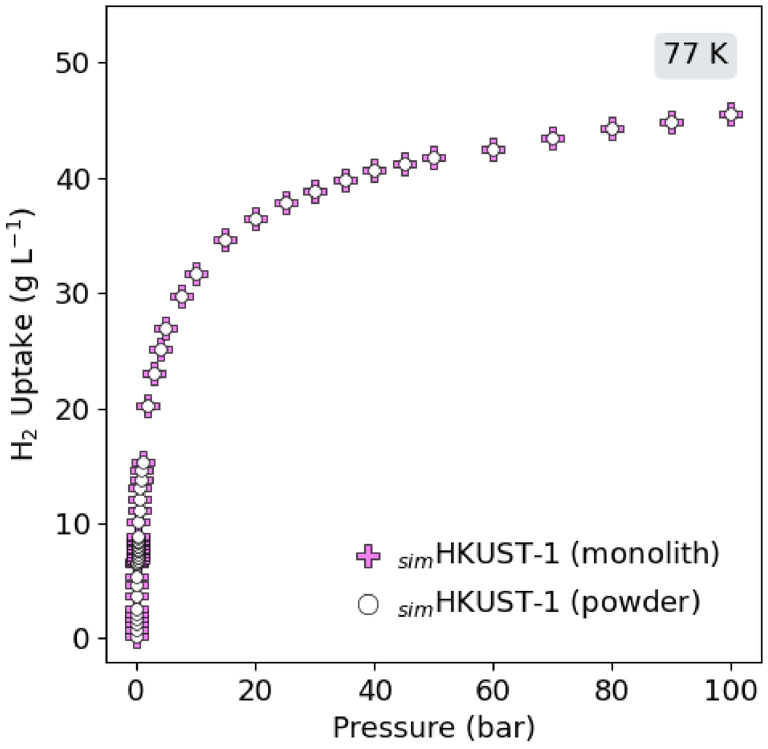

Figure S7. Simulated absolute 77 K H_2 adsorption isotherms for powdered HKUST-1 and $_{mono}$HKUST-1. Scaling factors applied based on the properties of powdered HKUST-1 (Pore Volume (V_{pore}) = 0.764 cm^3 g^{-1}; Crystal density ($\rho_{crystal}$) = 0.883 g cm^{-3}) and $_{mono}$HKUST-1 (Pore Volume (V_{pore}) = 0.634 cm^3 g^{-1}; Envelope density ($\rho_{envelope}$) = 1.064 g cm^{-3})

Table S5. Simulated 77 K N_2 adsorption isotherm for $_{mono}$HKUST-1.

Pressure (bar)	Loading (cm^3 g^{-1})
9.87E-05	182.34
1.97E-04	228.46
2.96E-04	240.56
3.95E-04	246.06
4.94E-04	251.46
5.92E-04	254.36
6.91E-04	256.39
7.90E-04	259.18
8.88E-04	260.42
9.87E-04	263.29
4.94E-03	299.38
9.87E-03	339.77
1.48E-02	364.87
1.97E-02	373.48
2.47E-02	382.31
4.94E-02	391.84
7.40E-02	394.55
9.87E-02	400.54
1.48E-01	400.28
1.97E-01	401.87
2.47E-01	402.34
2.96E-01	401.54
3.45E-01	403.43
3.95E-01	408.76
4.44E-01	407.54
4.94E-01	406.39
5.92E-01	408.75
6.91E-01	412.14
7.90E-01	410.18
8.88E-01	412.14
9.87E-01	413.33
1.00E+00	413.33

Table S6. Simulated 77 K H_2 adsorption isotherms for HKUST-1.

		Powder (Pore volume = 0.764 cm^3 g^{-1}; Density = 0.883 g cm^{-3})	Monolith (Pore volume = 0.634 cm^3 g^{-1}; Density = 1.064 g cm^{-3})
Pressure (bar)	Loading (cm^3 g^{-1})	g L^{-1}	g L^{-1}
0.001	2.14	0.19	0.19
0.005	8.89	0.79	0.79
0.010	14.52	1.28	1.28
0.015	19.37	1.71	1.71
0.020	23.89	2.11	2.11
0.025	29.68	2.62	2.63
0.050	42.27	3.73	3.74
0.075	52.78	4.66	4.67
0.100	61.08	5.39	5.40
0.150	73.09	6.45	6.47
0.160	75.07	6.63	6.64
0.170	76.14	6.72	6.73
0.180	80.17	7.08	7.09
0.190	79.91	7.06	7.07
0.200	83.27	7.35	7.37
0.210	85.38	7.54	7.55
0.220	85.61	7.56	7.57
0.230	88.09	7.78	7.79
0.240	90.79	8.02	8.03
0.250	90.71	8.01	8.02
0.260	94.44	8.34	8.35
0.270	95.85	8.46	8.48
0.280	96.46	8.52	8.53
0.290	99.01	8.74	8.76
0.300	100.45	8.87	8.88
0.400	114.81	10.14	10.15
0.500	126.52	11.17	11.19
0.600	137.31	12.13	12.15
0.700	148.10	13.08	13.10
0.800	155.98	13.77	13.80
0.900	165.88	14.65	14.67
1.000	172.66	15.25	15.27
2.000	229.66	20.28	20.31
3.000	260.98	23.04	23.08
4.000	284.69	25.14	25.18
5.000	304.48	26.89	26.93
7.500	336.93	29.75	29.80
10.000	359.47	31.74	31.79
15.000	392.10	34.62	34.68
20.000	412.45	36.42	36.48
25.000	428.95	37.88	37.94
30.000	440.05	38.86	38.92
35.000	451.25	39.85	39.91
40.000	459.67	40.59	40.66
45.000	466.32	41.18	41.24
50.000	473.50	41.81	41.88
60.000	481.57	42.52	42.59
70.000	492.39	43.48	43.55
80.000	501.87	44.32	44.39
90.000	508.39	44.89	44.96
100.000	515.37	45.51	45.58

Principal Component Analysis (PCA)

We performed Principal Component Analysis (PCA) using the data generated from the high-throughput screening of 2940 MOFs at the following conditions: adsorption at 25/50/100 bar and 77 K with desorption at 5 bar and 160 K. The raw data used for the PCA can be found at: https://aam.ceb.cam.ac.uk/mofexplorer.html - please refer to Part 1 of the dataset. PCA was performed using the Principal Component Analysis Visualization Tool which can be found at [16]: https://hydrogen-storage-pca.herokuapp.com, considering the following properties: pressure (bar), density (g cm^{-3}), gravimetric deliverable capacity (GDC, g g^{-1}), void fraction, accessible surface area per mass (m^2 g^{-1}), accessible surface area per volume (m^2 cm^{-3}), largest cavity diameter (LCD, Å), volumetric deliverable capacity (VDC, g cm^{-3}), and the pore limiting diameter (PLD, Å). In this work, PCA was based on a correlation matrix.

Figure S8 shows the correlation plot for the selected geometric properties and pressure. We can see that the LCD, VDC, Acc. SA per mass, Acc. SA per volume, void fraction, and GDC are highly correlated among themselves. This is not surprising as these geometric properties are just different ways of characterizing the free space within the material. The pressure is not correlated to any of the geometric properties considered. However, the density is inversely correlated to the LCD, VDC, Acc. SA per mass, Acc. SA per volume, void fraction, and GDC. This is not surprising either, as a material with larger pores, will have a lower density.

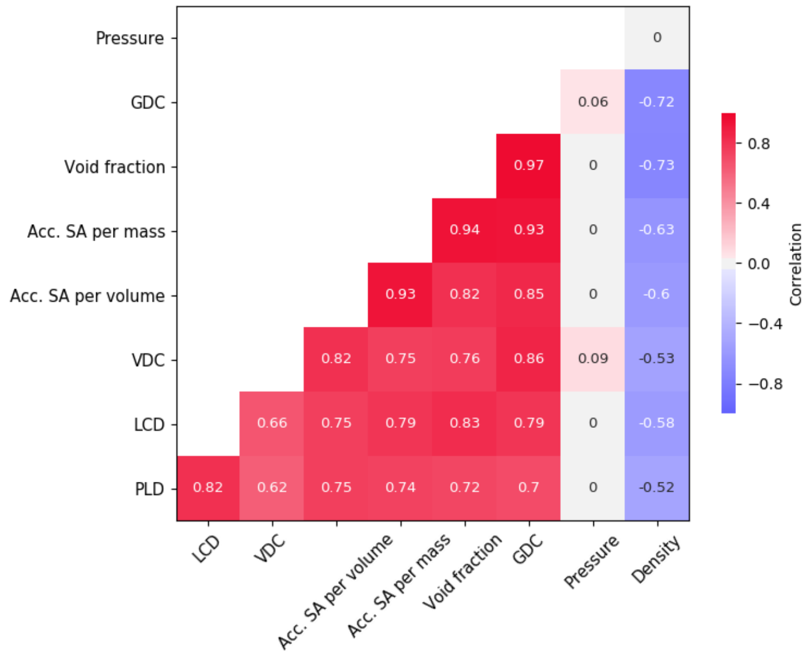

Figure S8. Correlation plot for the selected structural properties and pressure of the set of 2940 MOFs (DDEC + benchmark MOFs). The color scale indicates the correlation between the different variables; red color indicates a positive correlation whereas a blue color indicates a negative correlation. GDC = gravimetric deliverable capacity (g g^{-1}); VDC = volumetric deliverable capacity (g cm^{-3}); Acc. SA = accessible surface area (m^2 g^{-1}); LCD = largest cavity diameter (Å), and PLD = pore limiting diameter (Å).

PCA is commonly used for dimensionality reduction, helping us to choose the minimum number of variables needed to explain the maximum amount of variance in the dataset. **Figure S9** shows the cumulative scree plot, with the region in red depicting that a minimum of 4 principal components are needed to explain ~ 93% of the variance seen in the dataset. Using 1 principal component would help us to explain only ~70 % of the variance in the dataset. Hence, for further analysis we pick 4 principal components. The relationship between the properties and the principal components can be quantified/measured through loadings and contributions. **Table S7** lists the principal component loadings for each property with the properties contributing the most strongly to each principal component highlighted in red. Loadings are the correlation between the variables in the original dataset and the principal components in the new representation. **Figure S10** shows the % contribution of each feature to the 4 different principal components selected (PC1, PC2, PC3, and PC4). We can see that the GDC, void fraction, Acc. SA per mass, Acc. SA per volume, LCD, VDC, and PLD all exhibit strong positive loadings (**Table S7**), contributing in the range of 10-14 % each to PC1 (**Figure S10**). This means that an increase in any of these geometric properties would most likely lead to an increase in performance. The density on the other hand exhibits strong negative loadings, and contributes ~10% to PC1. This means that any further increase in density will most likely lead to a decrease in performance. The pressure exhibits very strong negative loadings, contributing ~ 98% to PC2. This implies that any change in PC2 scores for a material is primarily due to the influence of pressure.

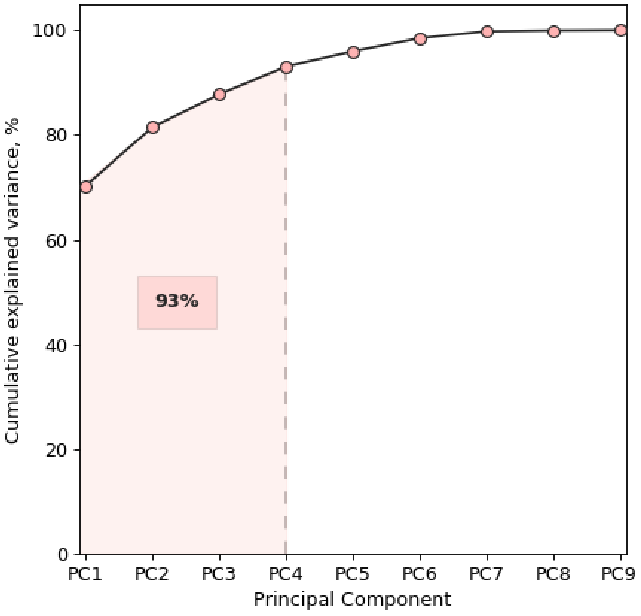

Figure S9. Cumulative scree plot of the explained variance as a function of the number of principal components. The region shaded in red depicts that 93% of the variance in the data can be explained using 4 principal components.

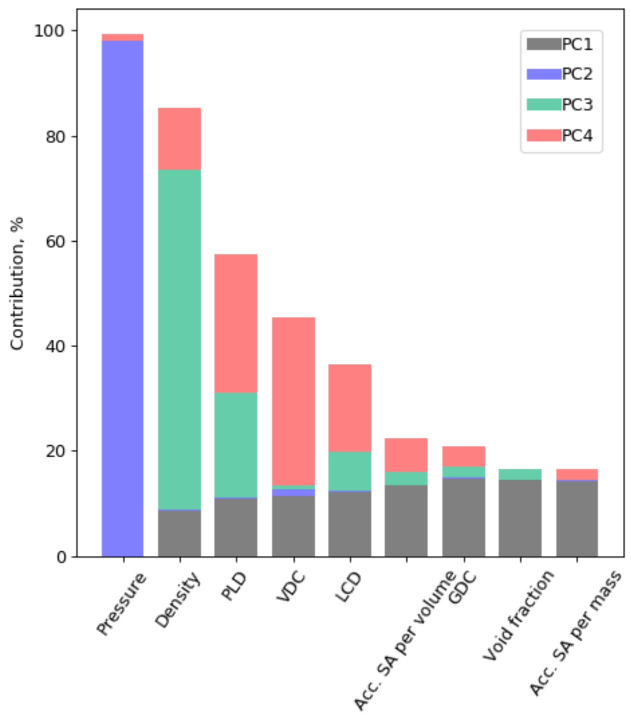

Figure S10. Contributions plot depicting the contribution (%) of each variable to the different principal components (PC) selected; PC1, grey; PC2, blue; PC3, green and PC4, red. GDC = gravimetric deliverable capacity (g g^{-1}); VDC = volumetric deliverable capacity (g cm^{-3}); Acc. SA = accessible surface area (m^2 g^{-1}); LCD = largest cavity diameter (Å), and PLD = pore limiting diameter (Å).

Table S7. Table of principal component loadings for each feature. Column 1 lists the feature; Columns 2, 3, 4, and 5 list the principal component loadings of PC1, PC2, PC3, and PC4 for each feature. The features contributing strongly (loading > 0.3 or < –0.3) to each principal component have been highlighted in red.

Feature	Principal component loadings			
	**PC1**	**PC2**	**PC3**	**PC4**
Gravimetric deliverable capacity (g g^{-1})	0.965333	-0.04743	-0.10903	-0.13388
Void fraction	0.956711	0.02589	-0.10736	-0.00428
Accessible surface area per mass (m^2 g^{-1})	0.951095	0.026489	0.032366	-0.09372
Accessible surface area per volume (m^2 cm^{-3})	0.921337	0.01651	0.120688	-0.17462
Largest Cavity Diameter (Å)	0.878027	0.041308	0.204748	0.280619
Volumetric deliverable capacity (g cm^{-3})	0.84849	-0.11037	0.065679	-0.39006
Pore limiting diameter (Å)	0.826988	0.045531	0.334709	0.353832
Pressure (bar)	0.024562	-0.9958	0.001479	0.079814
Density (g cm^{-3})	-0.73989	-0.03351	0.603263	-0.23429

Figure S11 is a scatter plot showing the scores (PC1, PC2) for each of the 2940 MOFs at three different adsorption pressures of 25 bar, 50 bar, and 100 bar with the top 1 % of materials at each pressure marked with an (x). Here, it is important to point out that the materials in the 25 bar and 50 bar datasets have positive PC2 scores whereas materials in the 100 bar dataset have negative PC2 scores. This implies that as the pressure increases from 50 to 100 bar, the pressure starts to negatively influence the performance of the materials. This tells us that there is an optimum pressure at which the performance of the materials peaks. This is most likely in the range of 50-55 bar, i.e. when most materials in the dataset have PC2 scores close to 0. We also analyzed the top 1 % of materials and found that the 50 bar dataset was best represented in that 90% of the structures present in the top 1 % of the 50 bar dataset were also present in either/both the top 1 % of the 25 bar and 100 bar datasets, with the top 5 materials being same for both the 50 bar and 100 bar datasets. We hope these insights will lead to the rational selection of process conditions for H_2 storage in benchmark MOF materials.

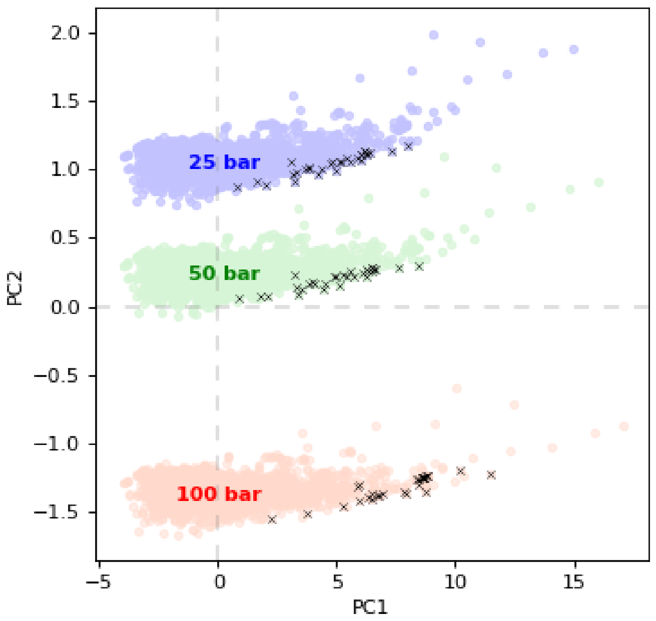

Figure S11. Scatter plot showing the scores (PC1, PC2) for each of the 2940 MOFs present in the database at three different adsorption pressures (T= 77 K) of 25 bar (blue), 50 bar (green), and 100 bar (red). Top 1% of the structures, in terms of working capacity – 77 K and 25/50/100 bar →160 K and 5 bar – are plotted in black (x).

NREL high-pressure gas adsorption measurements

The H_2 gas adsorption measurement at –197 °C at higher pressures (~5 to 105 bar **Figure S12**) was collected on a volumetric adsorption apparatus at the National Renewable Energy Laboratory (NREL). (The temperature of liquid N_2 at the elevation in Golden, CO is 75.6 K). The sample was packed in the custom-made sample holder in a dry box and transported to NREL in argon atmosphere. The sample holder includes a VCR diaphragm isolation valve, 1/8” tubing, and a 1/4” Swagelok VCR union and cap where the sample is located. There is a 2 micron filter gasket to ensure the sample remains in the sample holder. The sample was degassed to 120 °C at a heating rate of 2 °C/min, held at 120 °C for 12 hours, and cooled to ambient temperature at 2 °C/min. The H_2 adsorption isotherm was measured on a custom modified PCTPro 2000. The mass of the sample after measuring the isotherm was 248.5 mg. This system has been previously described in detail [17]. Modifications to the system include water-cooled copper jacket fittings to maintain extended temperature control of the gas dosing arm and the sample holder. For measurements at –197 °C, the bottom portion of the sample 2 holder containing the sample is placed in a liquid nitrogen bath. It is essential that the level of the liquid nitrogen bath remain constant throughout the measurement, as to not change the temperature profile of the sample holder as this would change the headspace calibration for the instrument. The same experimental setup was used to measure the isotherm at 303 K (1 to 140 bar, **Figure S13**) with the exception that a cryostat was used to control the temperature of the sample and the sample mass was 248.5 mg. It is important to note that the NREL experimentally measured values are excess amounts adsorbed (N_{exc}), which were transformed into total uptakes (N_{tot}) by using equation (5):

$$N_{tot} = N_{exc} + \rho(V_{envelope} - V_{skeletal}) \quad (5)$$

where ρ is the density of the gas at the given adsorption pressure and temperature, obtained from the National Institute of Standards and Technology (NIST) [18], and $V_{envelope}$ is the envelope volume and $V_{skeletal}$ is the He skeletal density of the material [19].

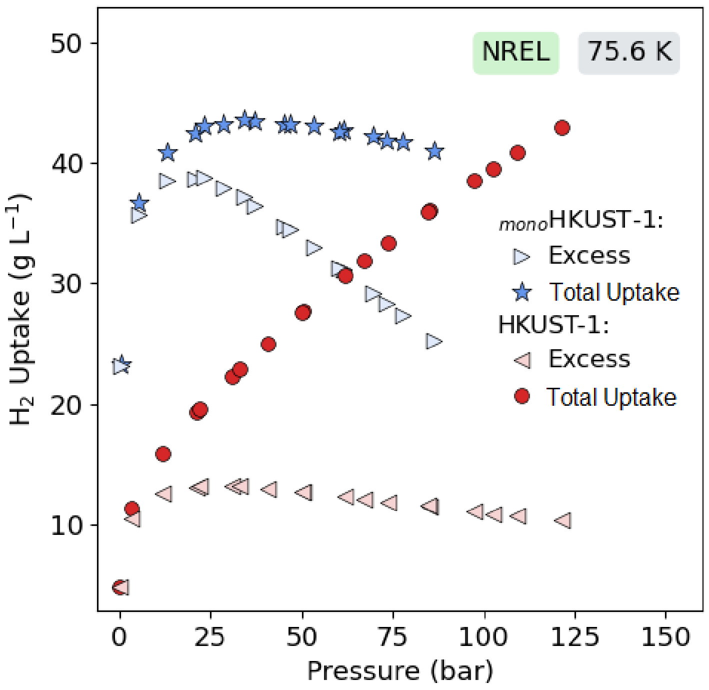

Figure S12. Excess (N_{exc}) and total (N_{tot}) H_2 adsorption isotherms collected by NREL for $_{mono}$HKUST-1 and powdered HKUST-1 at 75.6 K (liquid nitrogen measurement made at NREL, elevation 5768 feet (1758 m)). Open symbols represent excess uptake data, while closed symbols represent the calculated total uptake. Total adsorption values were calculated using the NREL method (Equation 5). An envelope density ($\rho_{envelope}$) of 1.07 g ml^{-1} was used to calculate the volumetric H_2 uptake of the $_{mono}$HKUST-1 material, while a packing density of 0.2979 g cm^{-3} was used to calculate the volumetric H_2 uptake of the powdered HKUST-1 material.

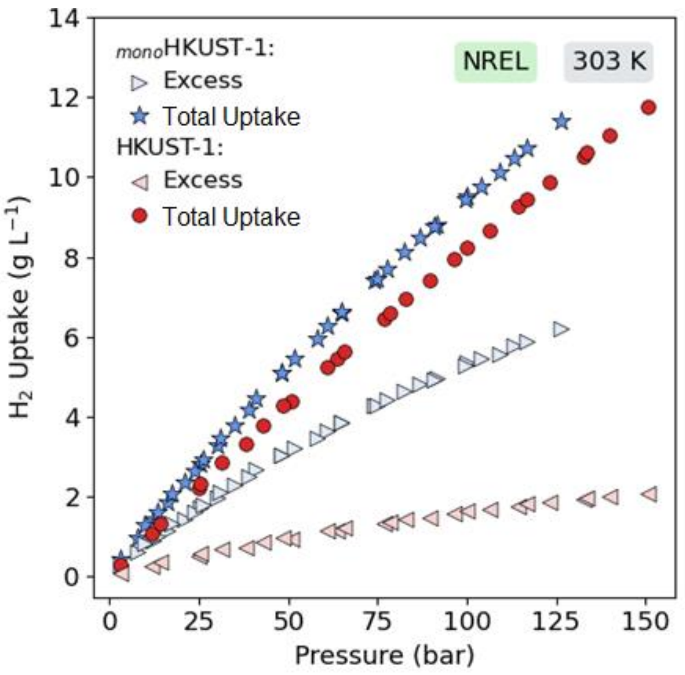

Figure S13. Excess (N_{exc}) and total (N_{tot}) H_2 adsorption isotherms collected by NREL for $_{mono}$HKUST-1 and powdered HKUST-1 at 303 K. Open circles represent excess uptake data, while closed circles represent the calculated total uptake. Total adsorption values were calculated using the NREL method (Equation 5). An envelope density ($\rho_{envelope}$) of 1.07 g ml^{-1} was used to calculate the volumetric H_2 uptake of the $_{mono}$HKUST-1 material, while a packing density of 0.2979 g cm^{-3} was used to calculate the volumetric H_2 uptake of the powdered HKUST-1 material.

Table S8. Excess 75.6 K H_2 adsorption data measured by NREL on $_{mono}$HKUST-1. Volumetric total adsorption (N_{tot}) capacity was calculated by NREL using Equation 6. Volumetric total capacity normalized by the envelope density of $_{mono}$HKUST-1 (1.07 g cm^{-3}).

Pressure	Gravimetric Excess[a]	Volumetric Excess	Volumetric Total[b]
(bar)	wt.% H_2	g H_2 L^{-1}	g H_2 L^{-1}
0.68	2.12	23.19	23.32
5.57	3.23	35.69	36.70
12.97	3.47	38.51	40.87
20.69	3.49	38.69	42.50
28.69	3.42	37.91	43.25
36.95	3.30	36.50	43.42
45.22	3.15	34.76	43.26
53.31	3.00	33.05	43.07
61.40	2.83	31.18	42.69
69.64	2.66	29.24	42.22
77.94	2.50	27.38	41.77
86.40	2.30	25.23	41.00
73.14	2.58	28.33	41.91
60.22	2.84	31.31	42.60
47.02	3.12	34.43	43.27
34.29	3.36	37.17	43.58
23.20	3.49	38.75	43.04

[a]Sample weight = 248.5 mg; [b]Experimental envelope density = 1.07 g cm^{-3}.

Table S9. Excess 303 K H_2 adsorption data measured by NREL on $_{mono}$HKUST-1. Volumetric total adsorption (N_{tot}) capacity was calculated by NREL using Equation 6. Volumetric total capacity normalized by the envelope density of $_{mono}$HKUST-1 (1.07 g cm^{-3}).

Pressure	Gravimetric Excess[a]	Volumetric Excess	Volumetric Total[b]
(bar)	wt.% H_2	g H_2 L^{-1}	g H_2 L^{-1}
3.08	0.03	0.29	0.43
7.87	0.06	0.62	0.97
12.13	0.08	0.89	1.42
16.32	0.11	1.14	1.85
21.12	0.13	1.43	2.35
25.30	0.16	1.72	2.83
30.12	0.19	1.98	3.29
35.04	0.21	2.27	3.79
48.03	0.28	3.02	5.09
61.01	0.34	3.65	6.26
74.01	0.40	4.27	7.41
87.05	0.45	4.82	8.49
100.10	0.50	5.35	9.53
113.21	0.54	5.78	10.48
126.37	0.57	6.19	11.39
109.13	0.52	5.58	10.12
91.79	0.46	4.96	8.81
74.87	0.40	4.27	7.44
58.01	0.32	3.47	5.95
41.15	0.25	2.67	4.45
23.75	0.15	1.62	2.65
13.47	0.09	1.01	1.60
26.21	0.17	1.79	2.93
38.92	0.23	2.49	4.18
51.77	0.30	3.21	5.44
64.75	0.36	3.85	6.61
77.74	0.41	4.41	7.70
90.79	0.46	4.93	8.75
103.88	0.51	5.44	9.77
116.98	0.55	5.88	10.73
99.73	0.49	5.27	9.44
82.47	0.43	4.63	8.11
64.96	0.36	3.86	6.63
48.02	0.28	3.03	5.10
31.09	0.20	2.12	3.47
17.60	0.12	1.32	2.09
10.00	0.08	0.84	1.28

[a]Sample weight = 248.5 mg; [b]Experimental envelope density = 1.07 g cm^{-3}.

Table S10. Excess 75.6 K H_2 adsorption data measured by NREL on powdered HKUST-1. Volumetric total adsorption (N_{tot}) capacity was calculated by NREL using Equation 6. Volumetric total capacity normalized by the particle packing density for powdered HKUST-1 (0.2979 g cm^{-3}).

Pressure	Gravimetric Excess[a]	Volumetric Excess	Volumetric Total[b]
(bar)	wt.% H_2	g H_2 L^{-1}	g H_2 L^{-1}
0.20	1.58	4.78	4.84
3.28	3.38	10.44	11.36
11.90	4.04	12.55	15.96
21.45	4.20	13.07	19.31
31.16	4.23	13.15	22.30
40.88	4.17	12.98	25.07
50.63	4.09	12.72	27.72
62.05	3.97	12.32	30.65
73.56	3.84	11.90	33.42
85.30	3.71	11.48	36.05
97.15	3.60	11.13	38.57
109.19	3.47	10.73	40.84
121.38	3.36	10.35	42.93
102.67	3.53	10.89	39.59
84.55	3.73	11.56	35.95
67.10	3.92	12.15	31.90
49.97	4.10	12.75	27.55
33.05	4.23	13.16	22.89
22.03	4.24	13.21	19.62

[a]Sample weight = 217.7 mg; [b]Experimental particle packing density = 0.2979 g cm^{-3}.

Table S11. Excess 303 K H_2 adsorption data measured by NREL on powdered HKUST-1. Volumetric total adsorption (N_{tot}) capacity was calculated by NREL using Equation 6. Volumetric total capacity normalized by the particle packing density for powdered HKUST-1 (0.2979 g cm^{-3}).

Pressure	Gravimetric Excess[a]	Volumetric Excess	Volumetric Total[b]
(bar)	wt.% H_2	g H_2 L^{-1}	g H_2 L^{-1}
3.02	0.03	0.07	0.28
11.86	0.08	0.25	1.07
24.98	0.17	0.49	2.21
38.04	0.24	0.72	3.33
50.97	0.31	0.94	4.39
63.85	0.38	1.14	5.44
76.70	0.44	1.32	6.45
89.54	0.49	1.47	7.41
106.49	0.56	1.67	8.67
123.37	0.62	1.85	9.88
139.91	0.67	2.01	11.03
150.90	0.69	2.08	11.75
132.65	0.65	1.94	10.53
114.47	0.59	1.77	9.26
96.38	0.53	1.58	7.95
78.47	0.46	1.37	6.61
60.74	0.38	1.14	5.24
43.07	0.29	0.86	3.80
25.47	0.19	0.57	2.32
14.16	0.12	0.36	1.34
31.50	0.23	0.69	2.85
48.70	0.32	0.96	4.27
65.87	0.41	1.21	5.64
83.03	0.48	1.44	6.96
100.09	0.55	1.64	8.24
116.99	0.61	1.82	9.46
133.69	0.66	1.98	10.63

[a]Sample weight = 217.7 mg; [b]Experimental particle packing density = 0.2979 g cm^{-3}.

University of Cambridge/University of Alicante gas adsorption studies

N_2 adsorption isotherms were undertaken at 77 K using a Micromeritics 3Flex instrument. High-pressure H_2 adsorption at 77 K, 195 K and 298 K was conducted using an HPVA II High Pressure Volume Analyser from Micromeritics. The temperature was controlled by using LN_2, dry ice/acetone mixture and a Julabo F25 HE circulator bath for 77 K, 195 K and 298 K, respectively. Prior to the analyses, the samples were activated overnight at 120 °C (vacuum) before measuring the mass, and then degassed in situ thoroughly before the gas adsorption. It is important to note that the experimentally measured values are excess amounts adsorbed (N_{exc}), which are transformed into absolute uptakes (N_{abs}) by using equation (6):

$$N_{abs} = N_{exc} + \rho V_{pore} \qquad (6)$$

where ρ is the density of the gas at the given adsorption pressure and temperature, obtained from the National Institute of Standards and Technology (NIST) [18], and V_{pore} is the pore volume of the adsorbent [20].

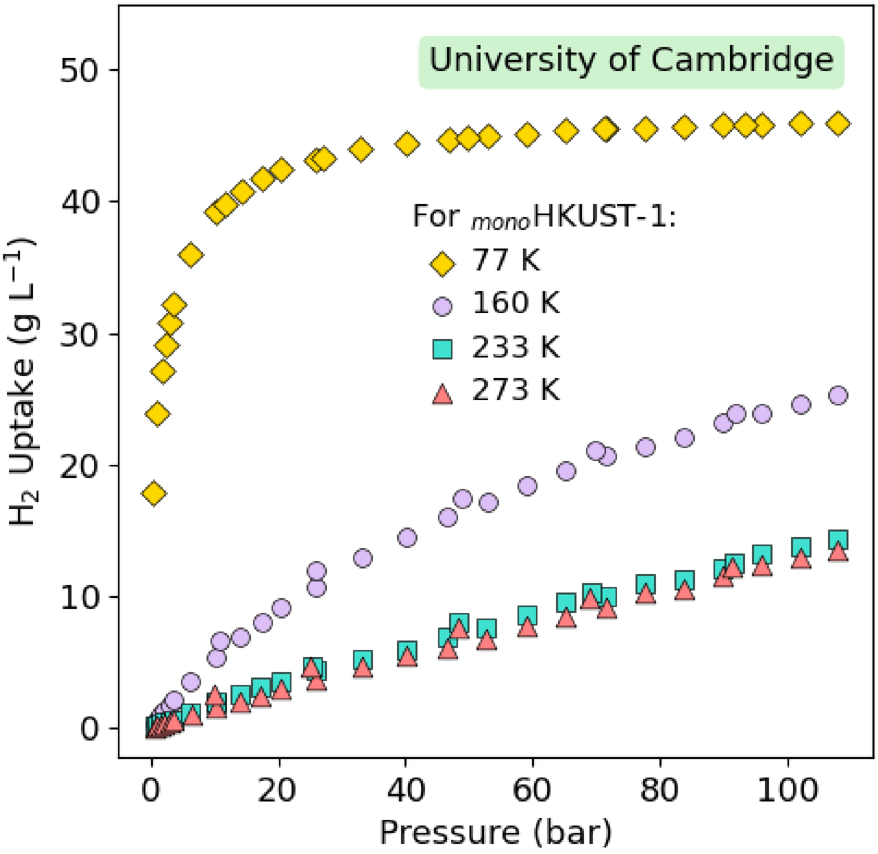

Figure S14. Absolute (N_{abs}) H_2 adsorption isotherms collected at the University of Cambridge for *mono*HKUST-1 at 77, 160, 233 and 273 K. Absolute adsorption values were calculated using Equation 6. An envelope density ($\rho_{envelope}$) of 1.064 g ml^{-1} and total pore volume (V_{pore}) of 0.634 cm^3 g^{-1} was used to calculate the volumetric H_2 uptake of the *mono*HKUST-1 material.

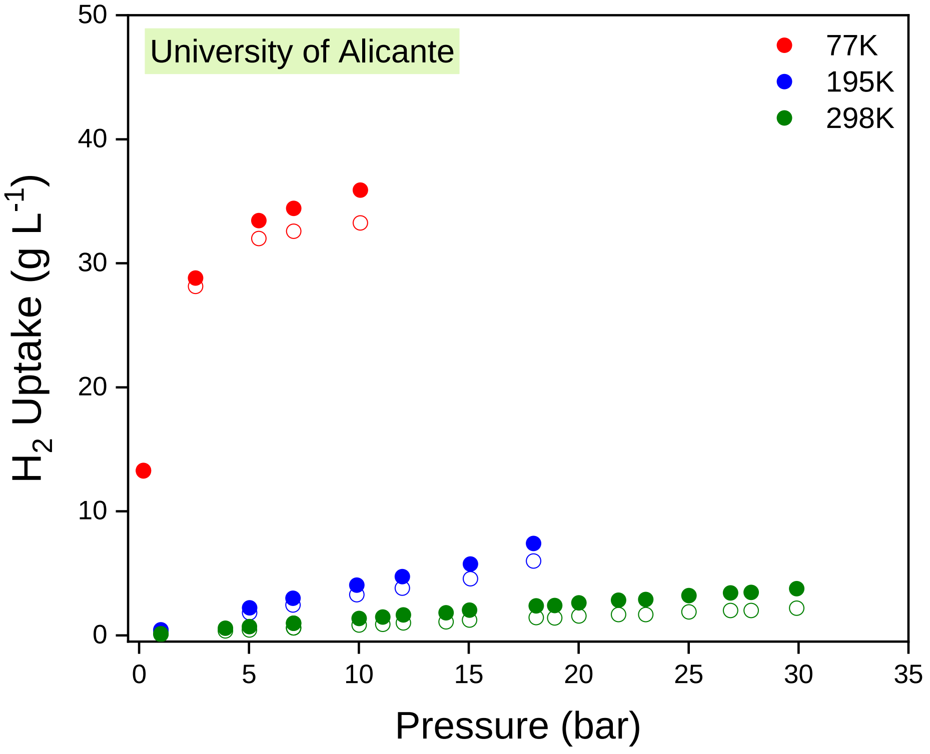

Figure S15. Excess (N_{exc}) and absolute (N_{abs}) H_2 adsorption isotherms collected at the University of Alicante for $_{mono}$HKUST-1 at 77, 195 and 298 K. Open circles represent excess uptake while closed circles represent absolute uptake. Absolute adsorption values were calculated using Equation 6. An envelope density ($\rho_{envelope}$) of 1.064 g ml^{-1} and total pore volume (V_{pore}) of 0.634 cm^3 g^{-1} was used to calculate the volumetric H_2 uptake of the $_{mono}$HKUST-1 material.

Table S12. Excess 75.6 K H_2 adsorption data measured by NREL on $_{mono}$HKUST-1. Volumetric absolute adsorption (N_{abs}) capacity was calculated by the University of Cambridge using Equation 5. Volumetric total capacity normalized by the envelope density for $_{mono}$HKUST-1 (1.07 g cm^{-3}).

Pressure	Gravimetric Excess[a]	Volumetric Excess	Volumetric Absolute[b,c]
(bar)	wt.% H_2	g H_2 L^{-1}	g H_2 L^{-1}
0.70	2.11	22.46	22.65
5.58	3.23	34.36	35.82
13.11	3.47	36.87	40.28
20.78	3.48	37.02	42.37
29.00	3.42	36.43	43.81
37.09	3.30	35.10	44.45
45.18	3.15	33.47	44.74
53.41	3.00	31.92	45.09
61.49	2.83	30.15	45.15
69.72	2.66	28.30	45.12
78.09	2.50	26.60	45.22
86.59	2.30	24.46	44.87

[a]Sample weight = 248.5 mg; [b]Pore volume = 0.621 cm^3 g^{-1}; [c]Experimental envelope density = 1.07 g cm^{-3}.

Table S13. Excess 303 K H_2 adsorption data measured by NREL on *mono*HKUST-1. Volumetric absolute adsorption (N_{abs}) capacity was calculated by the University of Cambridge using Equation 5. Volumetric total capacity normalized by the envelope density for *mono*HKUST-1 (1.07 g cm^{-3}).

**Pressure**	**Gravimetric Excess**[a]	**Volumetric Excess**	**Volumetric Absolute**[b,c]
**(bar)**	**wt.% H_2**	**g H_2 L^{-1}**	**g H_2 L^{-1}**
3.13	0.25	0.26	0.43
8.06	0.57	0.61	1.03
16.46	1.06	1.12	1.99
21.23	1.32	1.41	2.52
24.85	1.59	1.69	2.99
26.34	1.65	1.75	3.13
30.29	1.84	1.95	3.53
35.39	2.12	2.26	4.10
38.85	2.32	2.47	4.48
48.23	2.82	3.00	5.49
51.85	2.98	3.17	5.84
61.07	3.40	3.61	6.74
64.86	3.57	3.80	7.12
74.08	3.97	4.22	7.99
77.87	4.10	4.36	8.31
86.76	4.47	4.76	9.14
41.32	2.47	2.63	4.76
100.10	4.97	5.28	10.31
103.89	5.05	5.38	10.58
112.95	5.37	5.71	11.35
117.06	5.47	5.82	11.64
126.29	5.76	6.12	12.38
108.99	5.20	5.54	10.98
99.61	4.90	5.21	10.21
91.70	4.62	4.92	9.54
82.32	4.28	4.56	8.73
74.74	3.98	4.23	8.04
64.70	3.55	3.78	7.09
57.45	3.22	3.42	6.37
47.74	2.79	2.97	5.44
31.44	1.94	2.07	3.70
23.87	1.49	1.59	2.83
17.94	1.18	1.26	2.20
13.66	0.94	1.00	1.71
10.20	0.75	0.80	1.33

[a]Sample weight = 248.5 mg; [b]Pore volume = 0.621 cm^3 g^{-1}; [c]Experimental envelope density = 1.07 g cm^{-3}.

Table S14. Absolute adsorption data measured at the University of Alicante on $_{mono}$HKUST-1 at 77, 195 and 298 K. Volumetric absolute adsorption (N_{abs}) capacity was calculated by the University of Cambridge using Equation 5. Volumetric total capacity normalized by the envelope density for $_{mono}$HKUST-1 (1.07 g cm^{-3}).

77 K		195 K		298 K	
Pressure	Volumetric Absolute[a,b]	Pressure	Volumetric Absolute[a,b]	Pressure	Volumetric Absolute[a,b]
(bar)	g H_2 L^{-1}	(bar)	g H_2 L^{-1}	(bar)	g H_2 L^{-1}
0.20	14.14	0.99	0.36	0.99	0.71
2.58	30.64	5.03	1.81	5.03	0.96
5.46	35.56	7.00	2.43	7.04	1.37
7.04	36.63	9.91	3.27	10.01	1.65
10.07	38.17	11.98	3.81	12.03	2.03
		15.08	4.56	15.04	2.38
		17.95	5.99	18.07	2.61
				20.01	2.82
				21.81	3.21
				25.02	3.40
				26.91	3.75

[a]Total pore volume = 0.621 cm^3 g^{-1}; [b]Sample mass = 0.5 g; Experimental envelope density = 1.07 g cm^{-3}.

Table S15. Absolute adsorption data measured at the University of Cambridge on $_{mono}$HKUST-1 at 77, 160, 233 and 273 K. Volumetric absolute adsorption (N_{abs}) capacity was calculated by the University of Cambridge using Equation 5. Volumetric total capacity normalized by the envelope density for $_{mono}$HKUST-1 (1.07 g cm^{-3}).

77 K		160 K		233 K		273 K	
**Pressure**	**Volumetric Absolute[a,b]**	**Pressure**	**Volumetric Absolute[a,b]**	**Pressure**	**Volumetric Absolute[a,b]**	**Pressure**	**Volumetric Absolute[a,b]**
**(bar)**	**g H_2 L^{-1}**	**(bar)**	**g H_2 L^{-1}**	**(bar)**	**g H_2 L^{-1}**	**(bar)**	**g H_2 L^{-1}**
0.36	17.83	0.56	0.37	0.60	0.13	0.60	0.08
1.05	23.97	0.99	0.64	1.00	0.21	1.00	0.15
1.72	27.14	1.53	0.98	1.52	0.30	1.52	0.23
2.30	29.09	2.18	1.36	2.17	0.42	2.17	0.32
2.90	30.74	2.89	1.76	2.90	0.53	2.89	0.45
3.56	32.19	3.59	2.16	3.59	0.68	3.58	0.57
6.19	36.04	6.26	3.54	6.34	1.22	6.35	0.99
10.29	39.16	10.30	5.35	10.31	1.91	10.32	1.57
14.19	40.83	14.14	6.87	14.04	2.56	14.02	2.09
17.46	41.76	17.40	8.08	17.26	3.08	17.24	2.51
20.45	42.39	20.42	9.14	20.33	3.51	20.32	2.94
25.83	43.21	25.94	10.68	26.01	4.36	26.03	3.72
32.88	43.92	33.08	12.91	33.17	5.25	33.18	4.68
40.10	44.42	40.09	14.47	40.06	5.99	40.15	5.50
46.74	44.75	46.63	15.99	46.53	6.87	46.56	6.14
52.95	44.99	52.88	17.22	52.74	7.60	52.73	6.79
59.09	45.18	59.01	18.47	58.97	8.66	58.96	7.72
65.23	45.34	65.19	19.53	65.21	9.55	65.24	8.41
71.44	45.47	71.50	20.64	71.45	10.03	71.47	9.15
77.67	45.58	77.66	21.37	77.66	10.97	77.67	10.24
83.86	45.68	83.75	22.13	83.73	11.34	83.76	10.57
89.97	45.76	89.85	23.17	89.79	12.12	89.84	11.55
96.01	45.83	95.95	23.87	95.90	13.18	95.98	12.42
102.04	45.89	102.04	24.62	101.93	13.84	101.93	13.00
107.91	45.95	107.98	25.37	107.94	14.31	107.88	13.47
93.45	45.80	91.97	23.86	91.52	12.54	91.30	12.20
71.31	45.47	69.69	21.10	69.14	10.25	68.99	9.94
49.86	44.88	48.82	17.46	48.37	8.00	48.28	7.57
26.99	43.35	26.05	12.00	25.38	4.66	25.21	4.71
11.57	39.80	10.92	6.65	10.23	2.07	10.07	2.60

[a]Total pore volume = 0.621 cm^3 g^{-1}; [b]Sample mass = 0.5 g; Experimental envelope density = 1.07 g cm^{-3}.

Dual-Process Langmuir Model

The single-gas DPL model describes the adsorption of gas *i* on a heterogeneous adsorbent that is composed of two homogeneous but energetically different sites. Assuming that the adsorbate-adsorbent free energy on each patch is constant, the amount adsorbed m_i for component *i* is given by

$$n_{ads}[{}^{mmol}/_{g}] = m_1 \frac{bP}{1+bP} + m_2 \frac{dP}{1+dP} \qquad (7)$$

where m_1 and b are respectively the saturation capacity and affinity parameter on site 1, m_2 and d are respectively the saturation capacity and affinity parameter on site 2, and P is the absolute pressure. All of the assumptions of the Langmuir model apply to each patch, and the two patches do not interact with each other. The Henry's law constant is simply the sum of those on each patch or site (i.e., [$(m_1b)_{\text{site 1}} + (m_2d)_{\text{site 2}}$]). In this formulation, the saturation capacity for each component on each site is allowed to be different. The free energy or affinity parameter for two different sites is expressed as:

$$b = b_0 \exp\left(\frac{Q_1}{RT}\right) \qquad (8)$$

$$d = d_0 \exp\left(\frac{Q_2}{RT}\right) \qquad (9)$$

where the subscript *b* and *d* represents the free-energy level of site 1 or 2, Q_1 and Q_2 are the adsorption energy of components on site 1 or 2, and b_o and d_o are the pre-exponential factor or adsorption entropy of component of site 1 or 2. In these calculations, site 1 always denotes the higher adsorbate-adsorbent free energy and site 2 always denotes the lower adsorbate-adsorbent free energy. For single-gas adsorption, this always makes the free energy of site 1 higher than that of site 2. The fitting parameters used can be found in **Table S16**.

Table S16. Dual-Process Langmuir Parameters for temperature dependent adsorption of H_2 on *mono*HKUST-1.

Parameter	Units	Input
m_1	mmol/g	12.18
m_2	mmol/g	9.75
b		1.61E-03
d		6.16E-06
Q_1	J/ mol	3200.14
Q_2	J/ mol	9051.90
b_o	1/bar	1.61E-03
d_o	1/bar	6.16E-06

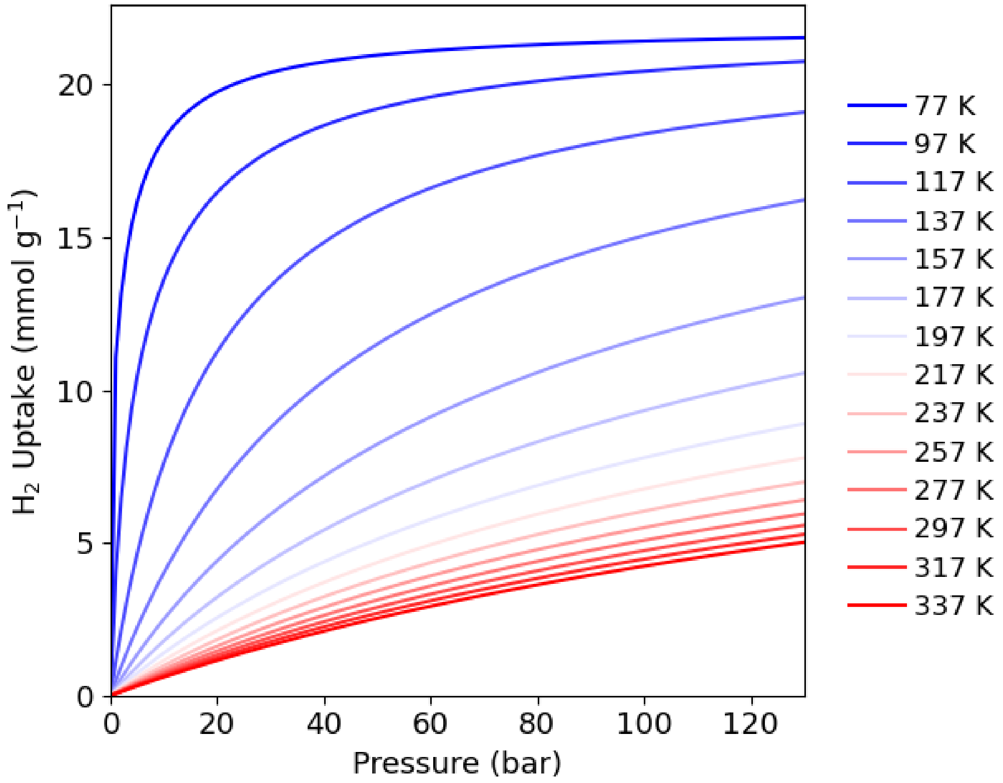

Figure S16. Dual-Process Langmuir (Equation 7) calculated H_2 isotherms. Dual-Process Langmuir fitting values are presented in **Table S16**.

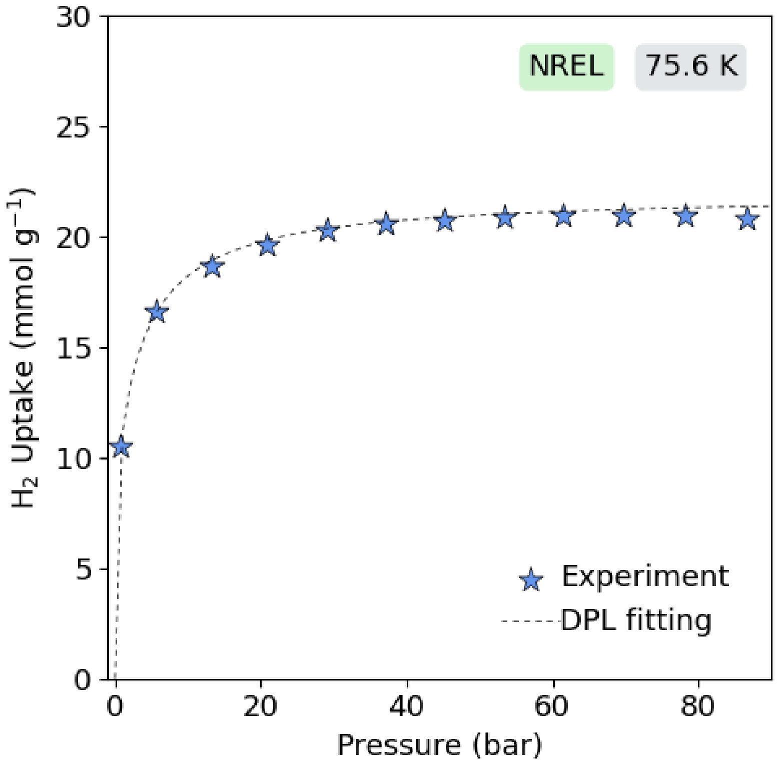

Figure S17. Dual-Process Langmuir (Equation 7) calculated H_2 isotherms and corresponding experimental absolute (N_{abs}) isotherms calculated using data collected by NREL at 75.6 K for $_{mono}$HKUST-1. Absolute adsorption values were calculated using excess adsorption collected at NREL and Equation 6. A total pore volume (V_{pore}) of 0.634 cm^3 g^{-1} was used to calculate the H_2 uptake of the $_{mono}$HKUST-1 material. Dual-Process Langmuir fitting values are presented in **Table S16**.

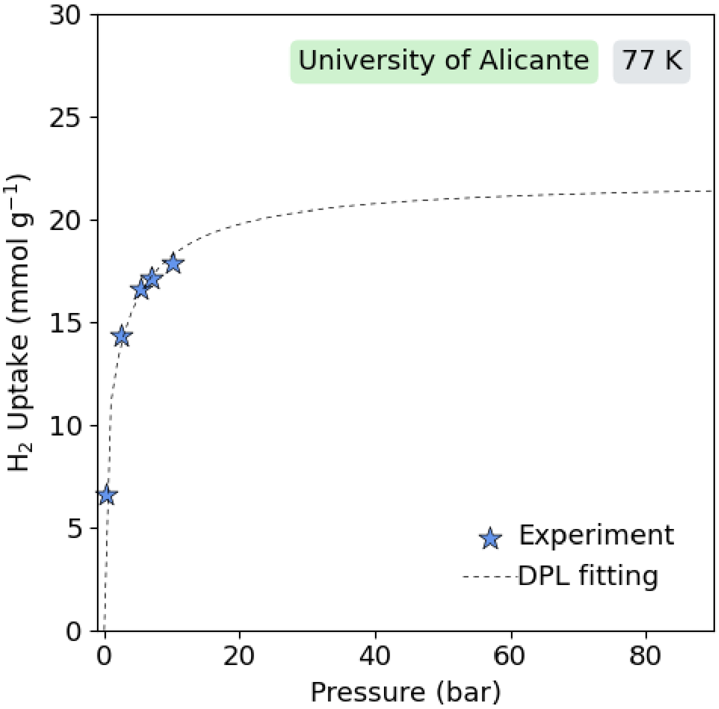

Figure S18. Dual-Process Langmuir (Equation 7) calculated H_2 isotherms and corresponding experimental absolute (N_{abs}) isotherms calculated using data collected by University of Alicante at 77 K for $_{mono}$HKUST-1. Absolute adsorption values were calculated using excess adsorption collected at the University of Alicante and Equation 6. A total pore volume (V_{pore}) of 0.634 cm^3 g^{-1} was used to calculate the H_2 uptake of the $_{mono}$HKUST-1 material. Dual-Process Langmuir fitting values are presented in **Table S16**.

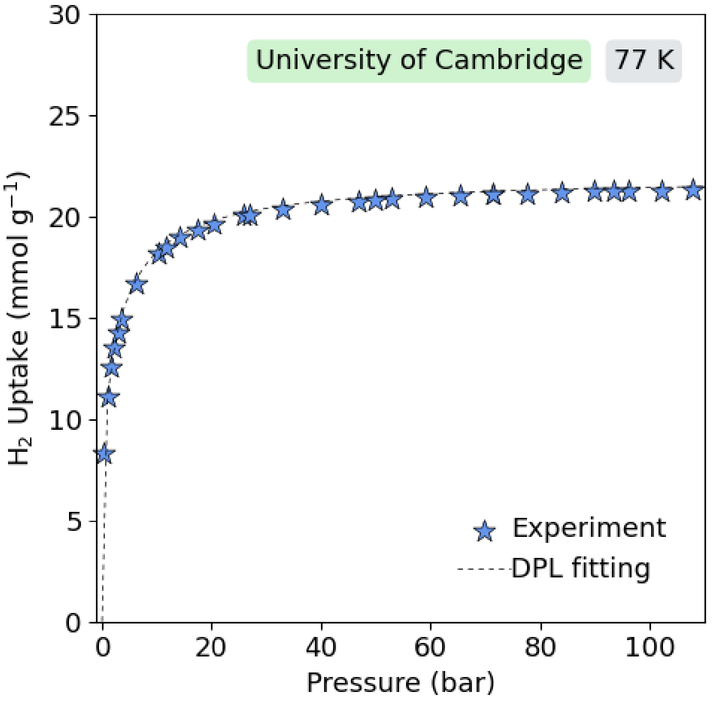

Figure S19. Dual-Process Langmuir (Equation 7) calculated H_2 isotherms and corresponding experimental absolute (N_{abs}) isotherms calculated using data collected by University of Cambridge at 77 K for $_{mono}$HKUST-1. Absolute adsorption values were calculated using excess adsorption collected at the University of Cambridge and Equation 6. A total pore volume (V_{pore}) of 0.634 cm^3 g^{-1} was used to calculate the H_2 uptake of the $_{mono}$HKUST-1 material. Dual-Process Langmuir fitting values are presented in **Table S16**.

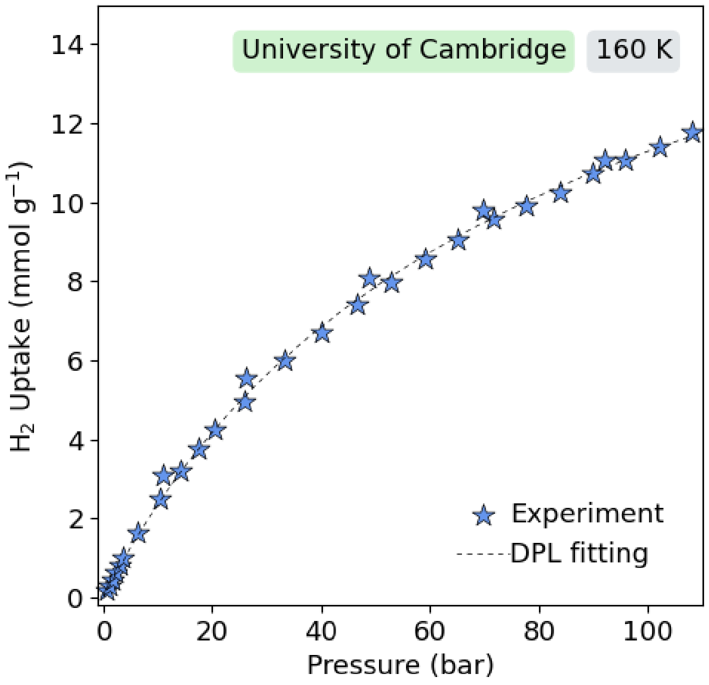

Figure S20. Dual-Process Langmuir (Equation 7) calculated H_2 isotherms and corresponding experimental absolute (N_{abs}) isotherms calculated using data collected by University of Cambridge at 160 K for *mono*HKUST-1. Absolute adsorption values were calculated using excess adsorption collected at the University of Cambridge and Equation 6. A total pore volume (V_{pore}) of 0.634 cm^3 g^{-1} was used to calculate the H_2 uptake of the *mono*HKUST-1 material. Dual-Process Langmuir fitting values are presented in **Table S16**.

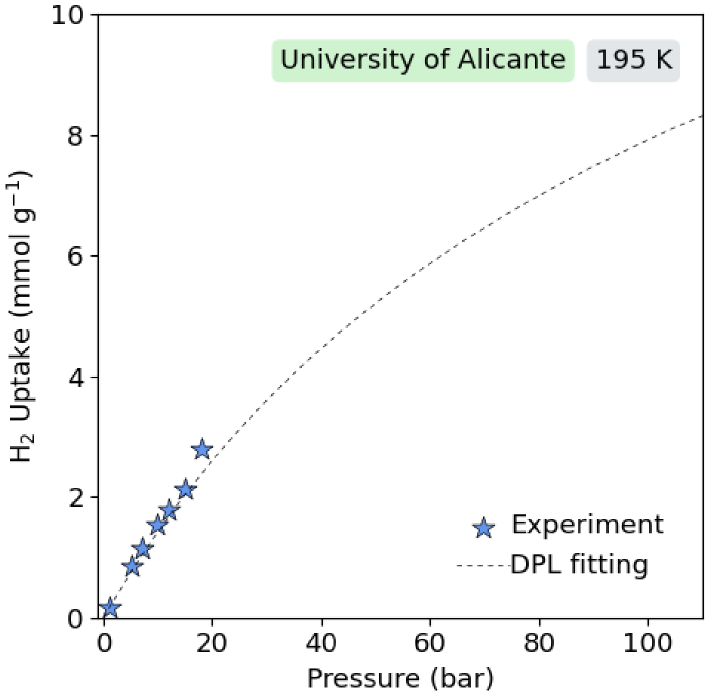

Figure S21. Dual-Process Langmuir (Equation 7) calculated H_2 isotherms and corresponding experimental absolute (N_{abs}) isotherms calculated using data collected by University of Alicante at 195 K for *mono*HKUST-1. Absolute adsorption values were calculated using excess adsorption collected at the University of Alicante and Equation 6. A total pore volume (V_{pore}) of 0.634 cm^3 g^{-1} was used to calculate the H_2 uptake of the *mono*HKUST-1 material. Dual-Process Langmuir fitting values are presented in **Table S16**.

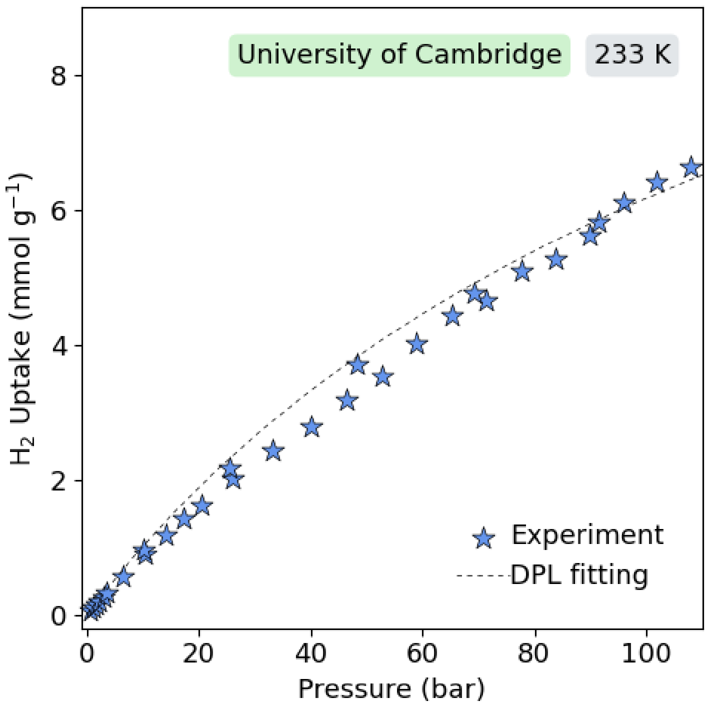

Figure S22. Dual-Process Langmuir (Equation 7) calculated H_2 isotherms and corresponding experimental absolute (N_{abs}) isotherms calculated using data collected by University of Cambridge at 233 K for $_{mono}$HKUST-1. Absolute adsorption values were calculated using excess adsorption collected at the University of Cambridge and Equation 6. A total pore volume (V_{pore}) of 0.634 cm^3 g^{-1} was used to calculate the H_2 uptake of the $_{mono}$HKUST-1 material. Dual-Process Langmuir fitting values are presented in **Table S16**.

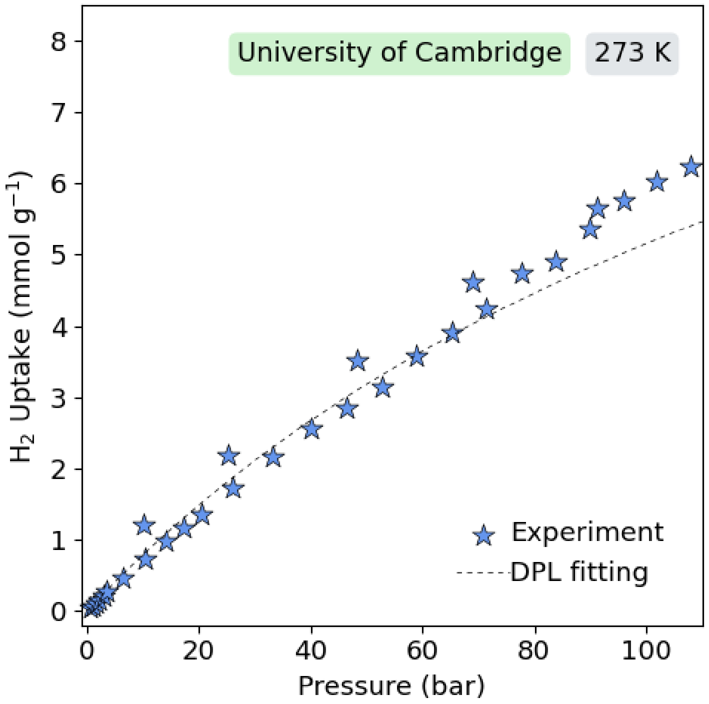

Figure S23. Dual-Process Langmuir (Equation 7) calculated H_2 isotherms and corresponding experimental absolute (N_{abs}) isotherms calculated using data collected by University of Cambridge at 273 K for $_{mono}$HKUST-1. Absolute adsorption values were calculated using excess adsorption collected at the University of Cambridge and Equation 6. A total pore volume (V_{pore}) of 0.634 cm^3 g^{-1} was used to calculate the H_2 uptake of the $_{mono}$HKUST-1 material. Dual-Process Langmuir fitting values are presented in **Table S16**.

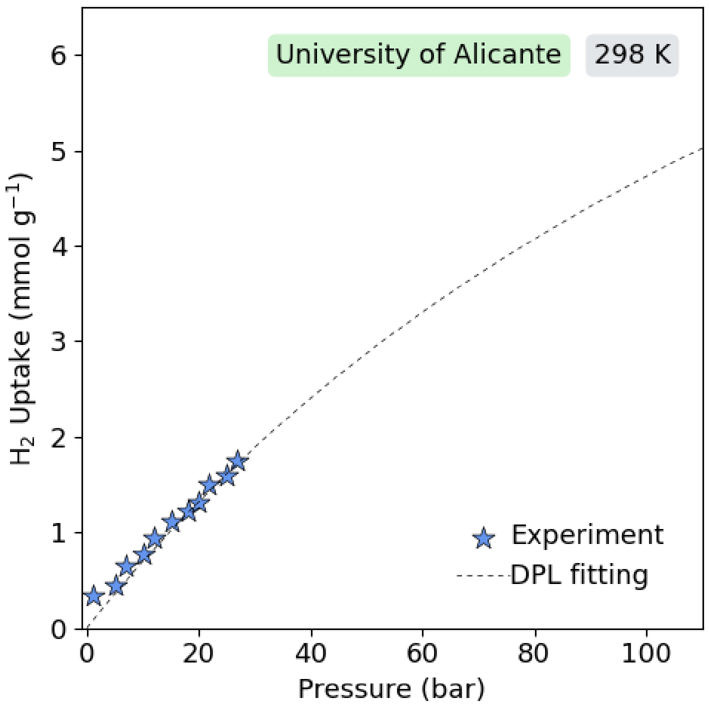

Figure S24. Dual-Process Langmuir (Equation 7) calculated H_2 isotherms and corresponding experimental absolute (N_{abs}) isotherms calculated using data collected by University of Alicante at 298 K for $_{mono}$HKUST-1. Absolute adsorption values were calculated using excess adsorption collected at the University of Alicante and Equation 6. A total pore volume (V_{pore}) of 0.634 cm^3 g^{-1} was used to calculate the H_2 uptake of the $_{mono}$HKUST-1 material. Dual-Process Langmuir fitting values are presented in **Table S16**.

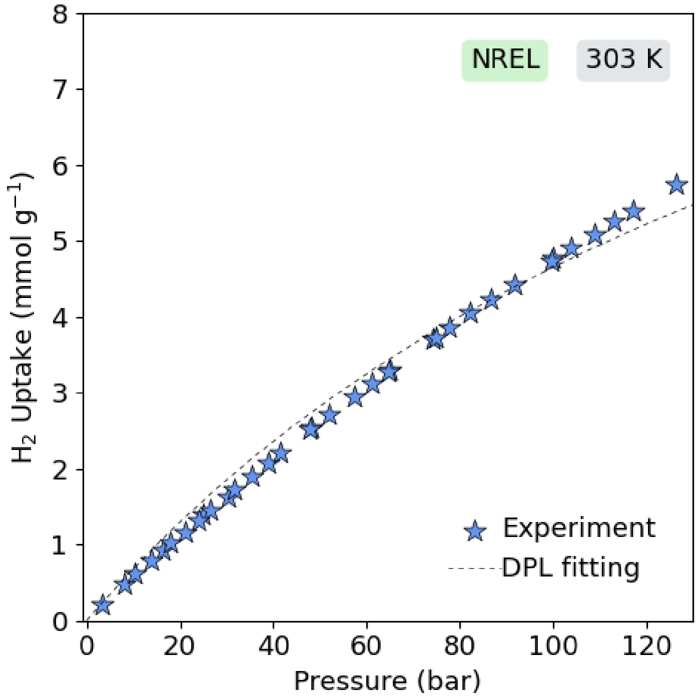

Figure S25. Dual-Process Langmuir (Equation 7) calculated H_2 isotherms and corresponding experimental absolute (N_{abs}) isotherms calculated using data collected by NREL at 303 K for $_{mono}$HKUST-1. Absolute adsorption values were calculated using excess adsorption collected at NREL and Equation 6. A total pore volume (V_{pore}) of 0.634 cm^3 g^{-1} was used to calculate the H_2 uptake of the $_{mono}$HKUST-1 material. Dual-Process Langmuir fitting values are presented in **Table S16**.

Mercury porosimetry density measurement

Mercury porosimetry was obtained up to a final pressure of 2,000 bar using an AutoPore IV 9500 instrument from Micromeritics. This technique was used to estimate the envelope density of the $_{mono}$HKUST-1 at atmospheric pressure. Prior to the analysis, all samples were activated overnight at 120 °C (vacuum) before measuring the mass, and then degassed *in situ* thoroughly before the mercury porosimetry. As a part of the NREL calculated measurements, the envelope density was confirmed to be 1.07 g/cm^3 by mercury porosity measurement performed by Particle Authority [21].

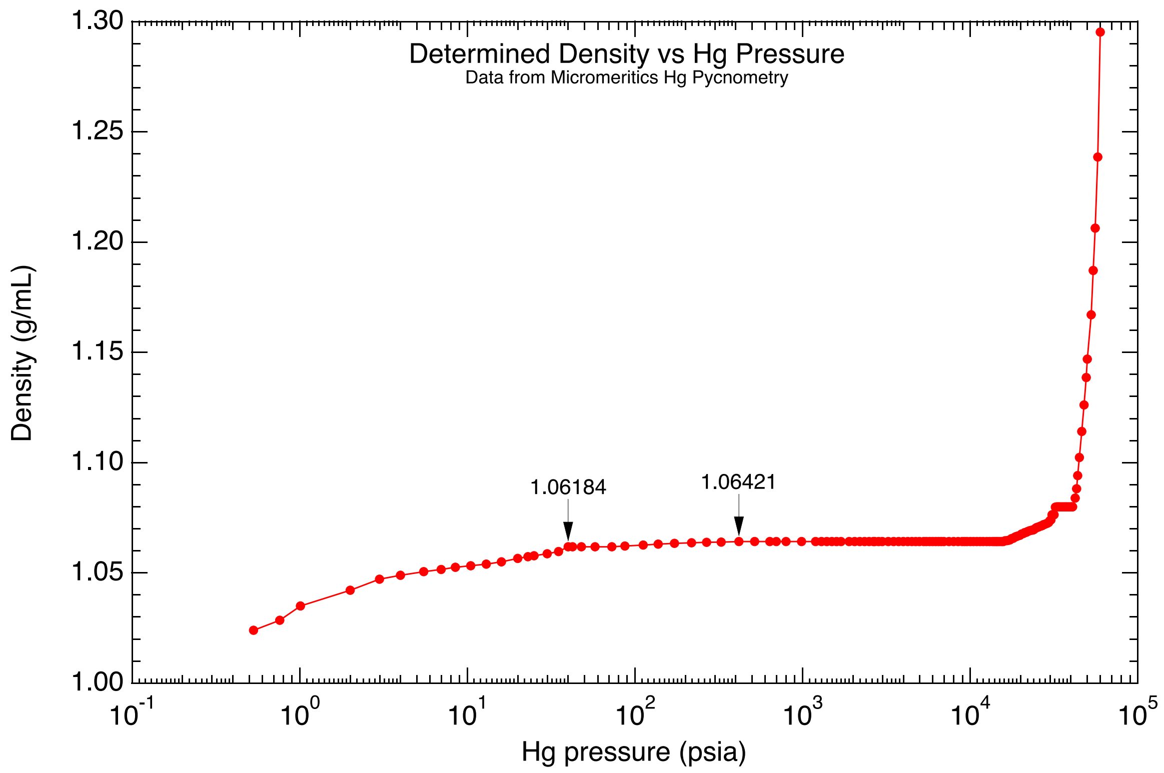

Figure S26. Mercury (Hg) porosimetry and envelope density measurements for $_{mono}$HKUST-1.

Table S17. NREL experimental density parameters for powdered and monolithic HKUST-1 used in high pressure H_2 adsorption calculations.

Material	Skeletal Density (g cm^{-3})	Density (g cm^{-3})
**HKUST-1 powder**	0.668	0.2979 Packing density
**$_{mono}$HKUST-1**	2.41	1.07 Envelope density

Solid State Nuclear Magnetic Resonance (NMR)

HKUST-1 samples were activated at 120 °C in flowing N_2 for 3-4 h. Samples were packed into 3.2 mm rotors inside a N_2 filled glovebag. ^{13}C NMR experiments were then performed at 16.4 T with a sample magic angle spinning frequency of 20 kHz. In each case a spin echo pulse sequence was used with a single rotor period as the interpulse delay time, and with a recyle delay of 100 ms. Owing to the large range of ^{13}C chemical shifts for these paramagnetic samples, two separate spectra were acquired with different transmitter frequencies. ^{13}C NMR spectra are referenced relative to the tertiary (eft-hand) carbon resonance in adamantane at 38.5 ppm.

Table S18. Summary of ^{13}C NMR chemical shifts from our work and Ref. [22].

	δ^{13}C/ppm		
**Site**	**HKUST-1 Powder**	***mono*HKUST-1**	**Ref. Powder**
**C1**	-87	-88	-89
**C2**	794	788	796
**C3**	228.3	228.7	228

X-ray Total Scattering Studies

Synchrotron X-ray total scattering data was collected at beamline 11-ID-B of the Advanced Photon Source (APS), Argonne National Laboratory (ANL), IL, USA [23], using a Perkin Elmer area detector and X-ray wavelength = 0.2115 Å. To allow 2D mapping, a monolithic sample of HKUST-1 was sliced into four parallel sections, each approximately 1 mm thick, and secured in the centre of a nylon washer between two sheets of Kapton™ tape (**Figure S27, Figure 4(a)**). Data suitable for Bragg diffraction analysis were collected at a sample-to-detector distance of ca. 947 mm, while data suitable for pair distribution function studies were collected at a sample-to-detector distance of ca. 197 mm. Using a 500 μm^2 X-ray beam, the cross sections were scanned both horizontally and vertically in steps of 500 μm to afford a map consisting of 500 μm^2 pixels. Data was collected across the entire sample holder, and the edges of the monolith sections were estimated based on the presence of (i) diffraction rings or (ii) PDF atom-atom distances consistent with Cu. A CeO_2 diffraction standard was used to calibrate experimental geometry in both cases and all scattering images were integrated using GSAS-2 [24]. Pair distribution functions, $G(r)$, were extracted from the total scattering data within xPDFsuite [25], with X-ray scattering measured for an empty sample holder used for background subtraction. $G(r)$

was calculated using scattering data in the range 0.1 Å^{-1} ≤ Q ≤ 22.7 Å^{-1}. Inspection of the diffraction patterns reveals a number of spurious peaks present around the edge of the mapped monoliths and are emphasised upon LeBail fitting of HKUST-1 lattice parameters to the data in Topas (**Figure S28**).To compare the relative presence of impurities, the normalized integral intensity of the spurious diffraction peak at Q = 0.57 Å^{-1} and the (222) peak (Q = 0.83 Å^{-1}) of HKUST-1 were fitted using the cumulative trapezoid method as implemented in the Python package scipy.integrate. (**Figure 4(e-h)**). To further probe the monolithic HKUST-1 PDF, we analyzed the data using previously described non-negative matrix factorization (NMF) techniques [26]. Two components were used to describe the data (**Figure 4 (d, i-l) & Figure S29**). Comparison of these maps (**Figure S30**) reveals reasonable corroboration between the PDF-NMF components and the spurious diffraction peaks observed, with component A having a mean Pearson correlation of 0.92 with the (222) peak of Slice 1 and 0.98 with the (222) peak of Slice 2, and component B having a mean Pearson correlation of 0.78 with the spurious peak of Slice 1 and 0.91 with the spurious peak of Slice 2.

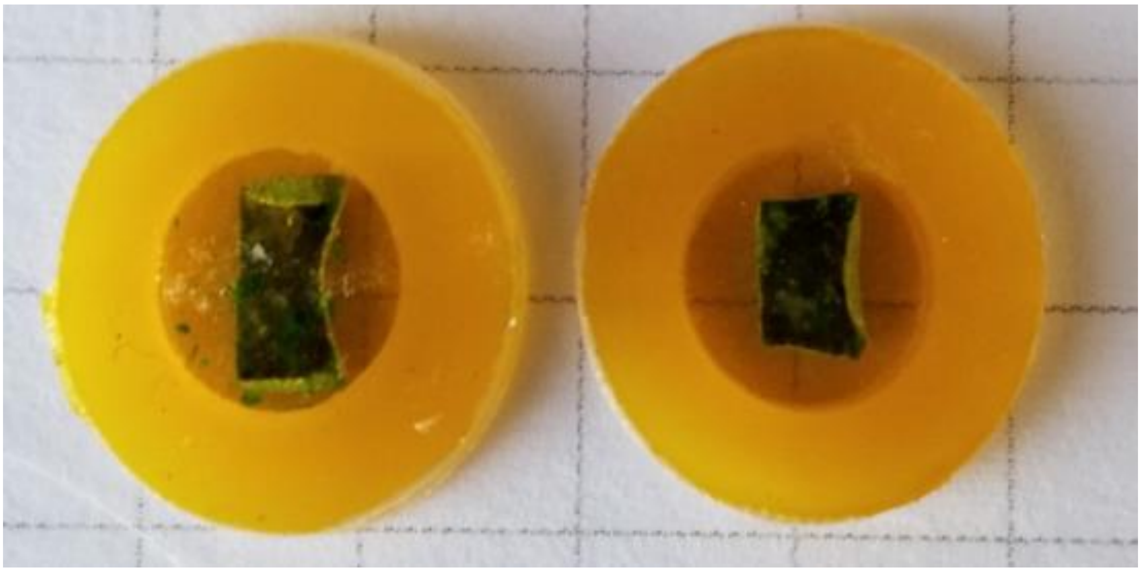

Figure S27. A sample of monolithic HKUST-1 was sliced to afford mapping across the cross section of the monolith at different points.

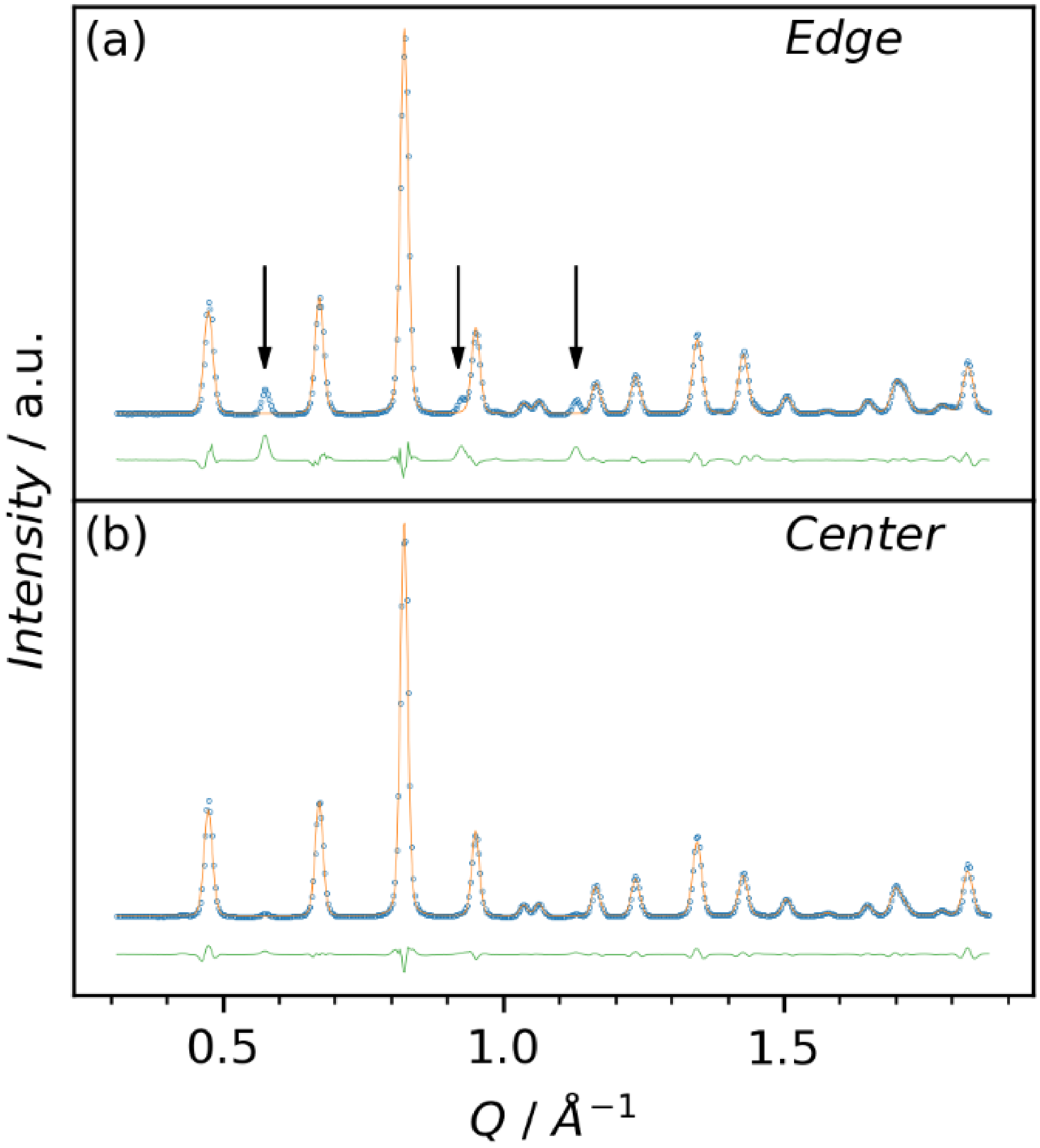

Fig. S28. LeBail fitted diffraction patterns of $_{mono}$HKUST-1 mapped at (**a**) the edge and (**b**) the center of *slice 1* which demonstrate the difference in intensity of spurious peaks associated with hydrolytic decomposition of the MOF (a, black arrows); these peaks are far less observable at the center of the monolithic sample when compared to the edge. (blue circles = experimental profile; orange line = fitted HKUST-1 profile; green line = observed – calculated intensities).

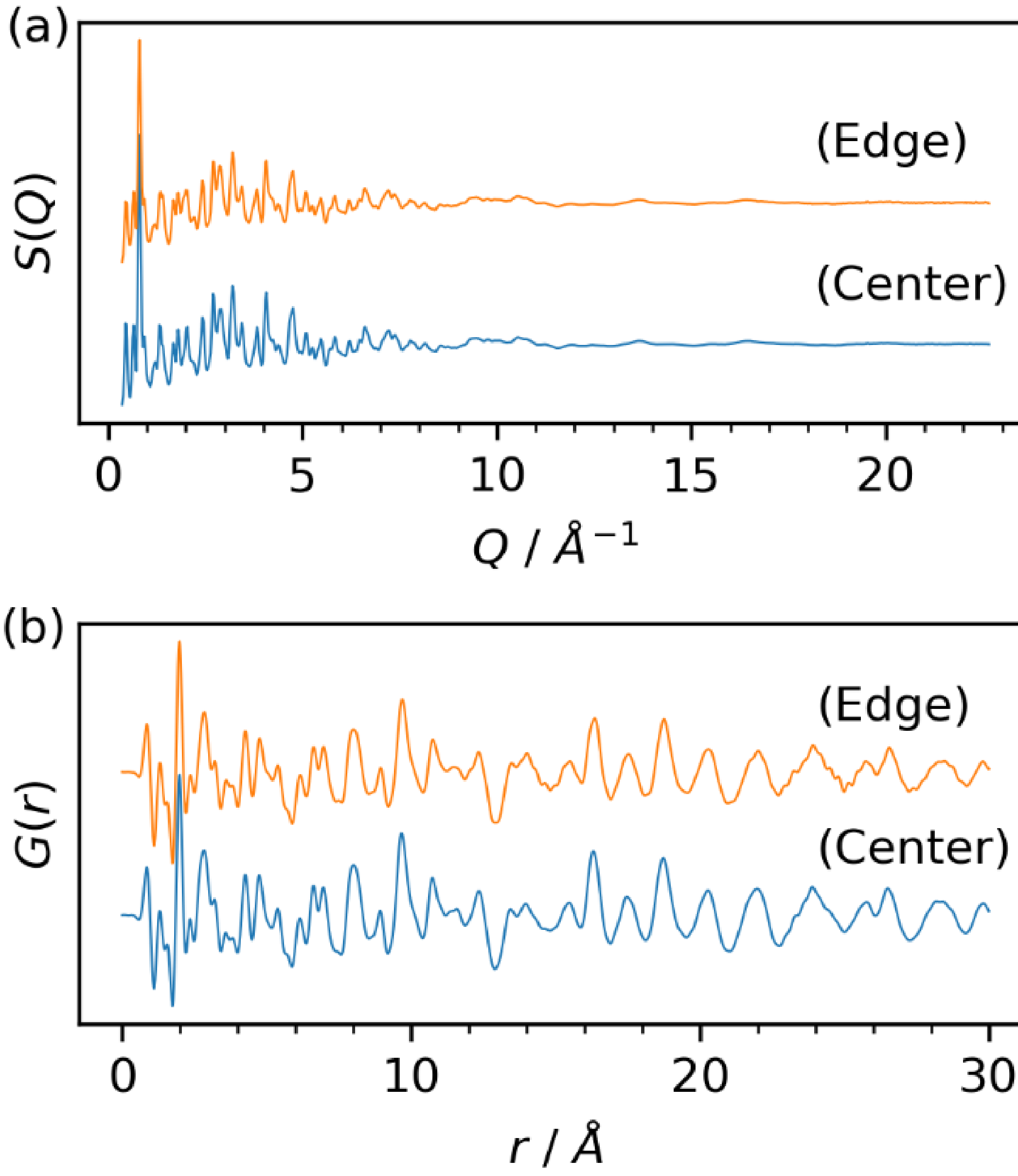

Fig. S29. (**a**) total structure function for samples collected at the edge and center of $_{mono}$HKUST-1. (**b**) Corresponding pair distribution functions. Neither raw functions exhibit substantial qualitative differences to correspond with observed spurious diffraction peaks.

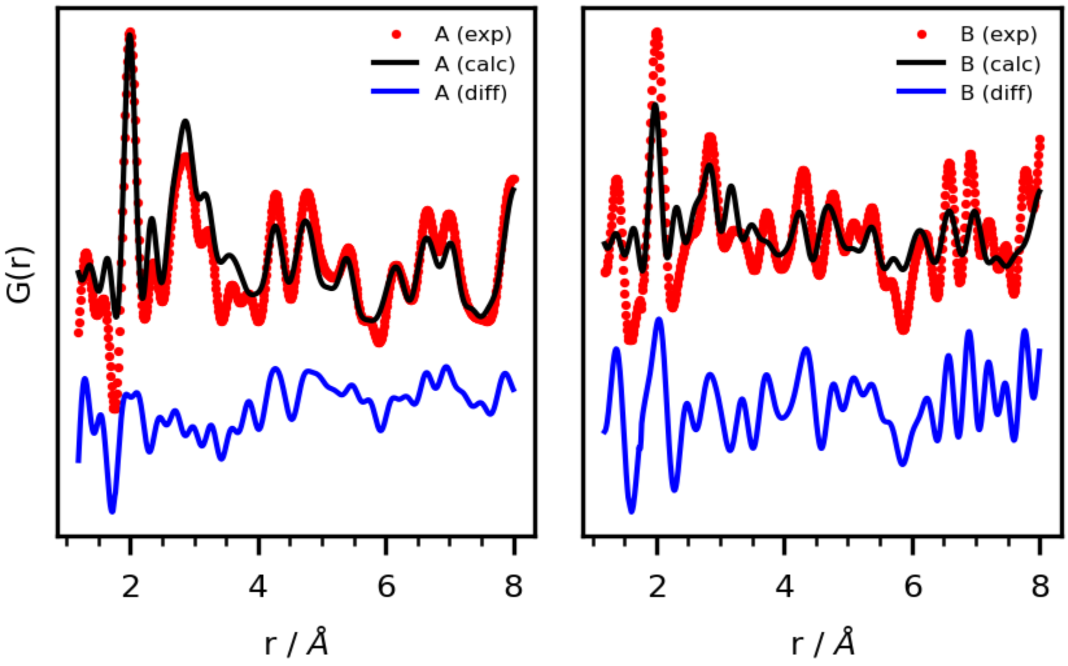

Figure S30. Pair distribution function, G(r), of component A (**a**, blue dots) and component B (**b**, red dots) with peaks associated with Cu-O (blue), Cu-Cu (red), and Cu-C (green) atom-atom distances in HKUST-1. In contrast to component A, the relative amplitude of Cu-O atom-atom distances is significantly reduced in component B which correlates with suggested decomposition of HKUST-1.

Small Angle X-Ray Scattering (SAXS) Studies

SAXS data was collected at the Materials Science beamline of the Paul Scherrer Institut (PSI), Switzerland, and at Synchrotron SOLEIL, France. SAXS data was collected at the Materials Science beamline of the Paul Scherrer Institut (PSI), CH, with an X-ray wavelength = 1.0000 Å as calibrated by the diffraction of a Si NIST standard on the high-resolution powder diffractometer. A total of 6 samples (3 powdered; 3 monolithic) were fitted using the IRENA package in Igor within the range 0.0009 $Å^{-1}$ < Q < 0.0170 $Å^{-1}$ (**Figure S31**). The SAXS measurements performed on the SWING beamline at Synchrotron SOLEIL were collected using a sample-to-detector distance of 6 m at a wavelength of 1.033 Å, a momentum transfer q range of 1.1×10^{-3}-0.15 $Å^{-1}$ was reached with the EIGERX 4 M detector. Size distribution was calculated from the X-ray scattering intensity (I) as a function of scattering vector (Q) such that:

$$I(Q) = |\Delta\rho|^2 \int_0^\infty |F(Q,r)|^2 V^2(r) N P\,(r) \Delta r \qquad (10)$$

Where $\Delta\rho$ = contrast, $F(Q,r)$ = scattering form factor, $V(r)$ is the particle volume, N is the total number of scattering particles, and $P(r)$ is the probability of occurrence of a scatterer size r, and were modelled with a log-normal distribution using a spheroidal form factor. The Guinier regions of these materials, the region which describes a particles radius of gyration (R_g), were outside the range probed and could

not be fit. The power law slope of *I(Q)* at low scattering angle for each sample was therefore fitted using the simplified relationship:

$$I = B(Q)^{-P} \qquad (11)$$

Where, intensity (*I*) can be fitted by a prefactor (*B*) across the scattering range (*Q*) and a power law slope (*P*) and indicated surface fractals for both powder and monolithic samples (*ca.* 3.6). Size distribution models were fitted with a log normal size distribution and spheroidal form factors. Powder samples consistently indicated two broad size distributions (mean diameters = *ca.* 24 nm and *ca.* 92 nm), whereas monolithic samples consisted of a single size distribution (diameters = *ca.* 20 nm) (**Figure S32**).

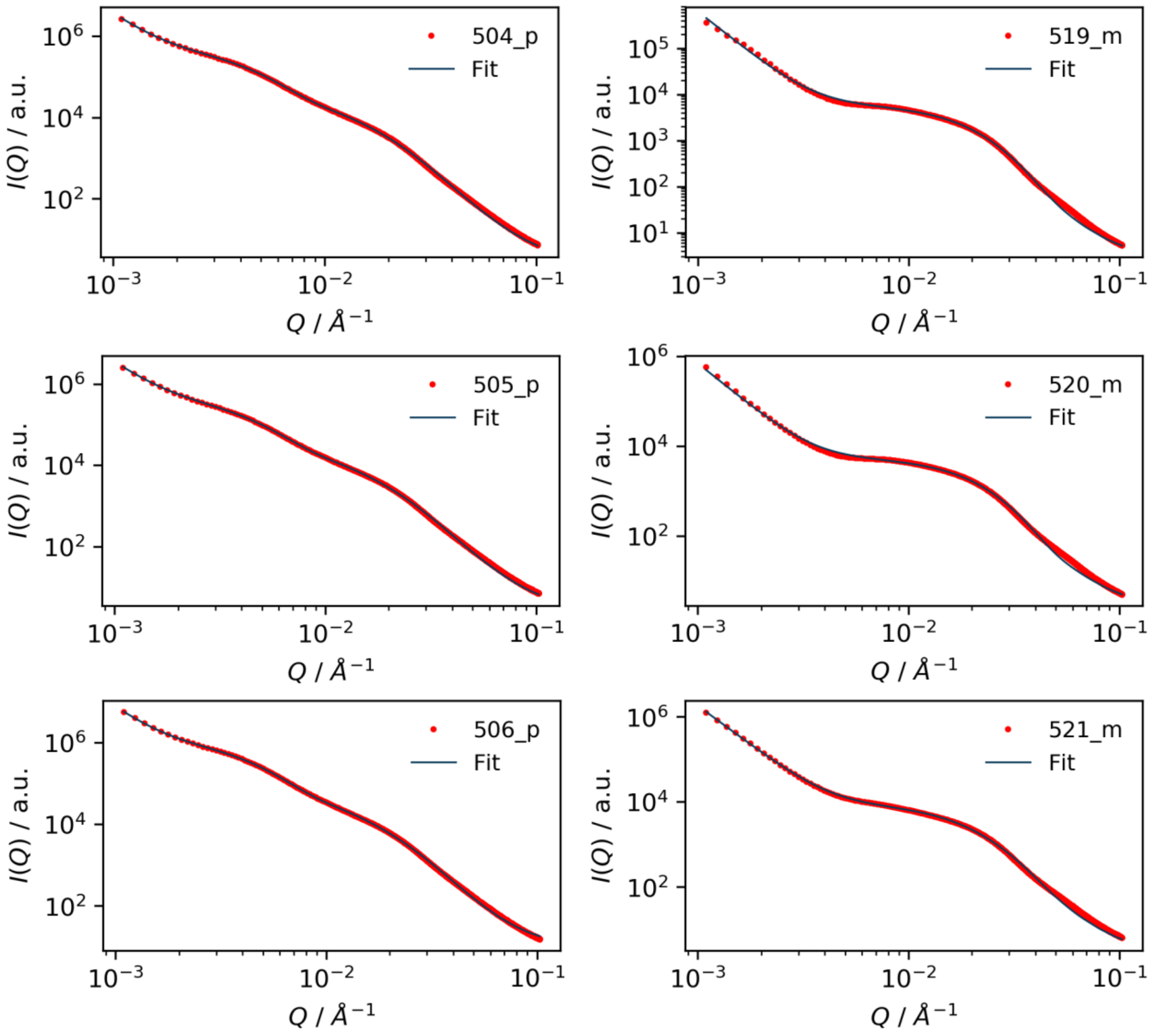

Figure S31. SAXS Fits for Powdered (p) and Monolithic (m) HKUST-1

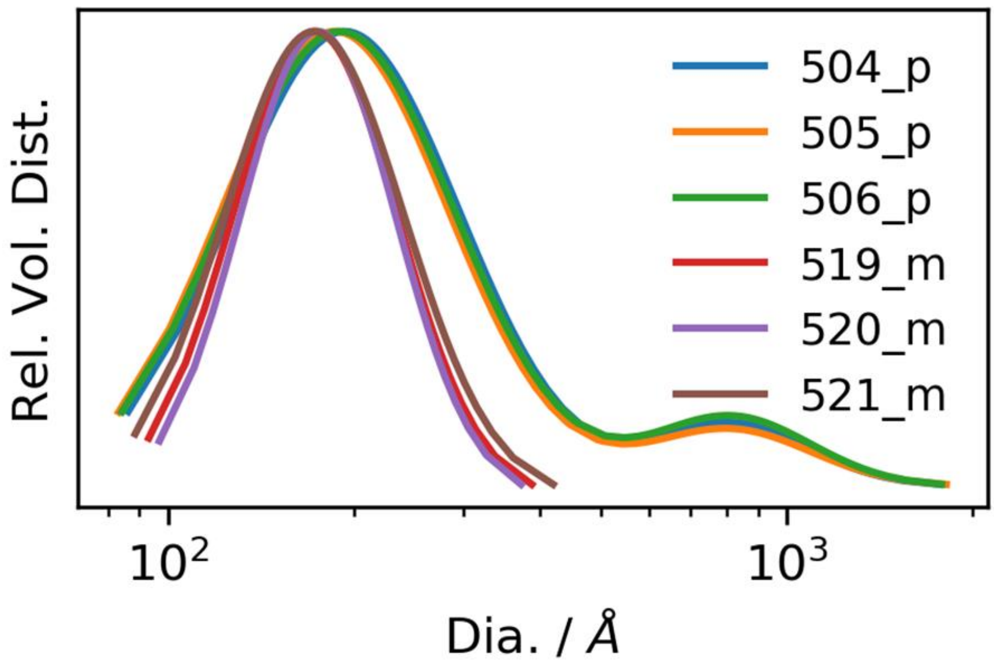

Figure S32. SAXS particle size distributions for (p) Powdered and (m) Monolithic HKUST-1.

Raman microscopy experimental

Raman microscopy was performed using a Horiba LabRAM HR Raman microscope equipped with an automated *xyz* stage (Märzhäuser). To simultaneously scan a range of Raman shifts, a 600 lines mm^{-1} rotatable diffraction grating along a path length of 800 mm was employed. Spectra were acquired using a Synapse CCD detector (1024 pixels) thermoelectrically cooled to −60 °C. Before spectra collection, the instrument was calibrated using the zero-order line and a standard Si(100) reference band at 520.7 cm^{-1}.

For single point measurements, spectra were acquired using a 532 nm laser (at a power of 0.2 mW), a 100× objective, and a confocal pinhole of 200 µm, over the range 50-4000 cm^{-1} (3 spectral windows) with an acquisition time of 60 seconds and 2 accumulations to improve the signal to noise ratio and remove the spikes due to cosmic rays. The spectral resolution in this configuration is better than 1.9 cm^{-1}. Spectra were averaged by collection from a minimum of two locations with similar colouration from optical images.

An interfacial spectroscopic map was acquired using a 532 nm laser (at a power of 0.2 mW), a 100x objective, and a confocal pinhole of 200 µm, over the range 190-1800 cm^{-1} (1 spectral window) at ~1.1 µm steps along a diagonal line ~22 µm in length (21 spectra). Changes in the topography were corrected using the ViewSharp™ and AutoFocus functionality within instrument software. As each individual spectrum was collected for 30 seconds, repeated once, the map required ~25 minutes of

acquisition time. The phase distribution within the map was evaluated using classic least squares (CLS) regression analysis within LabSpec6.5 software. The mean spectra obtained from the light blue and dark blue parts of the sample were used as loadings for the fitting analysis. The error in fitting was <0.6% for all spectra within the map.

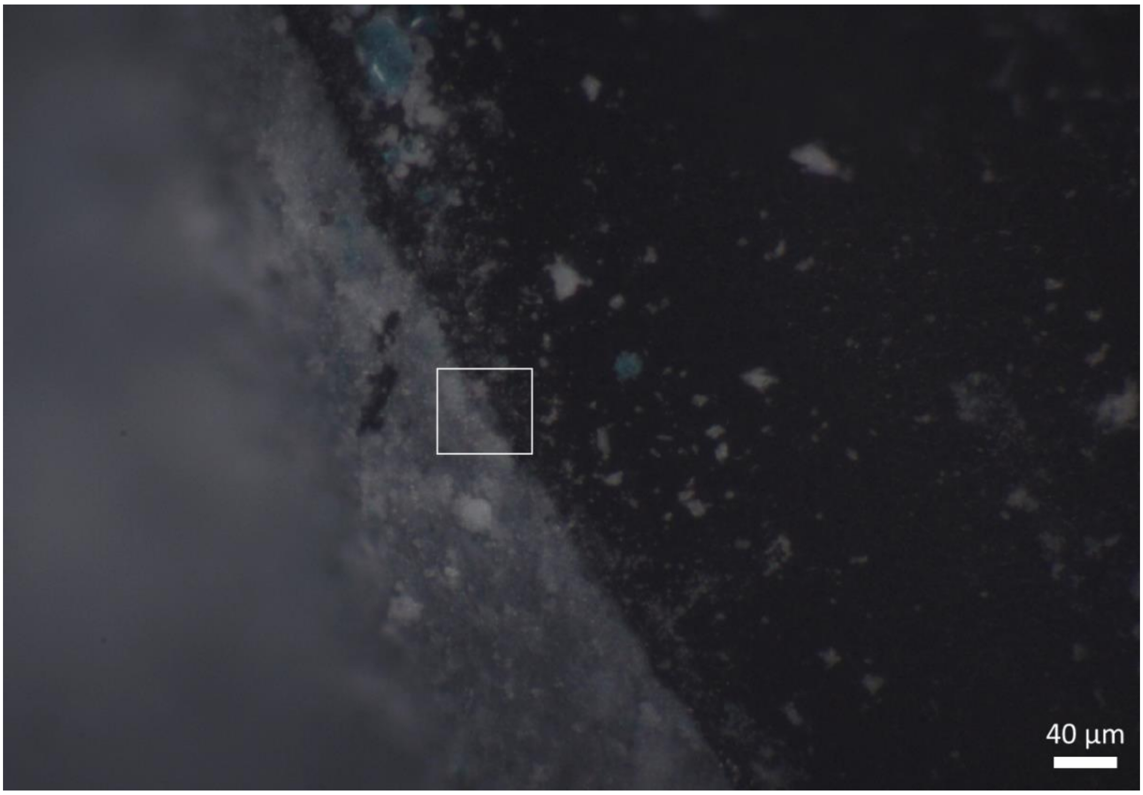

Figure S33. Raman microscopy image of *mono*HKUST-1 area analysed.

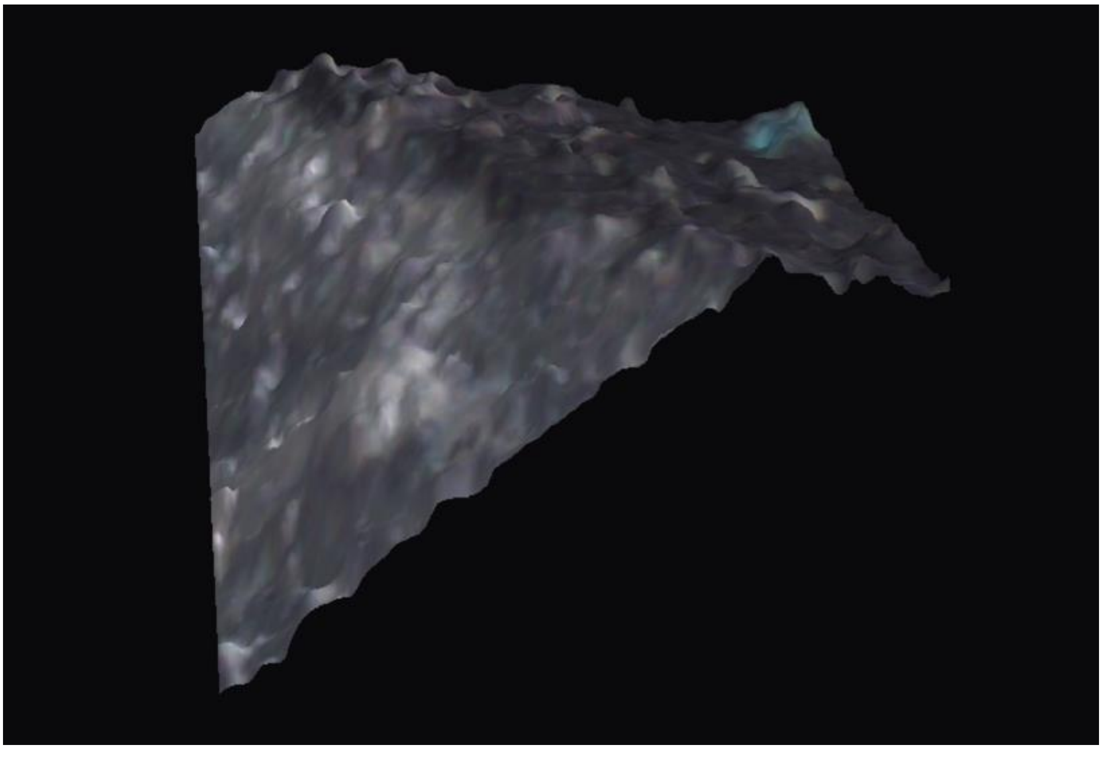

Figure S34. 3D mapping of *mono*HKUST-1 area analysed using Raman microscopy.

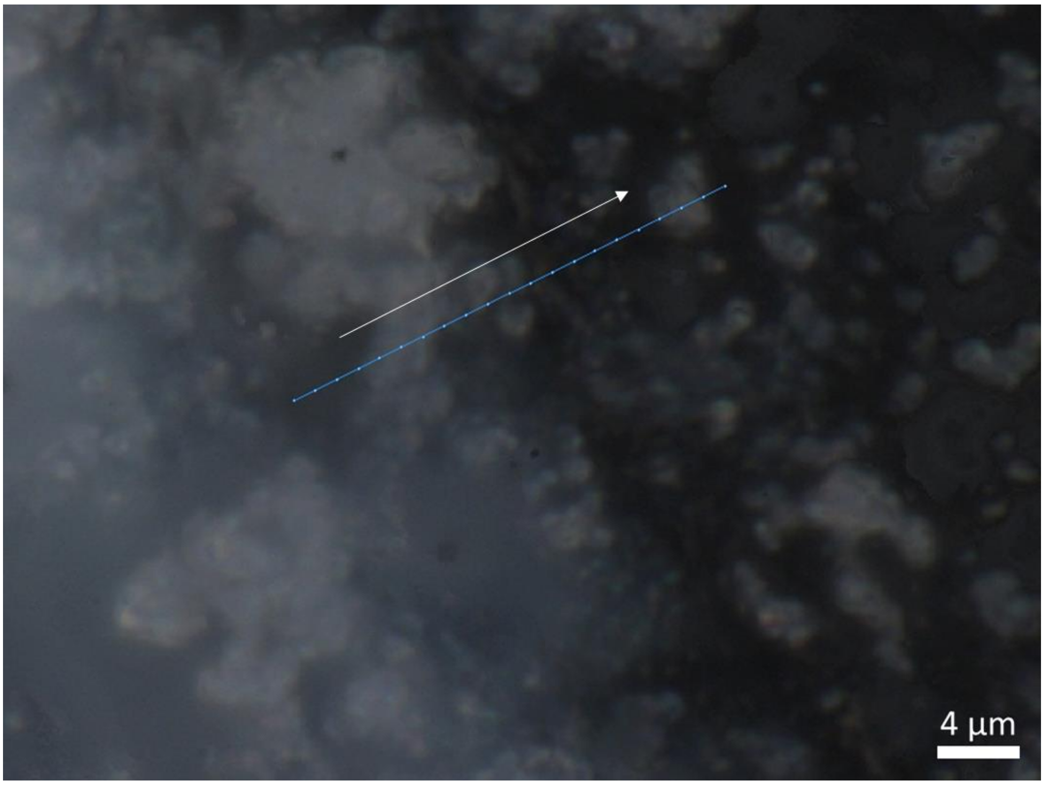

Figure S35. Raman probe pathway used during mapping of ${}_{mono}$HKUST-1 area during Raman microscopy.

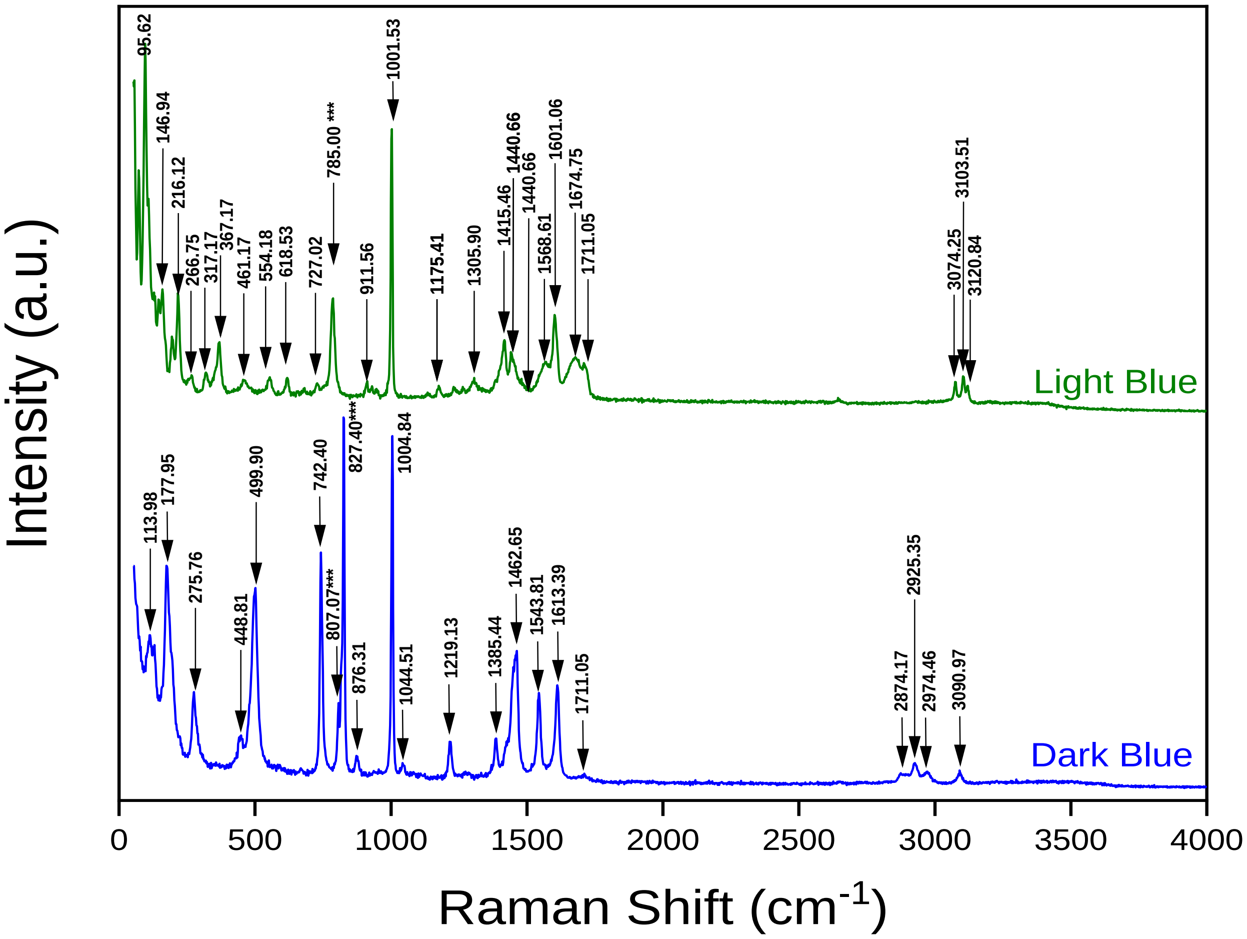

Figure S36. Raman spectra of light blue and dark blue areas observed in ${}_{mono}$HKUST-1.

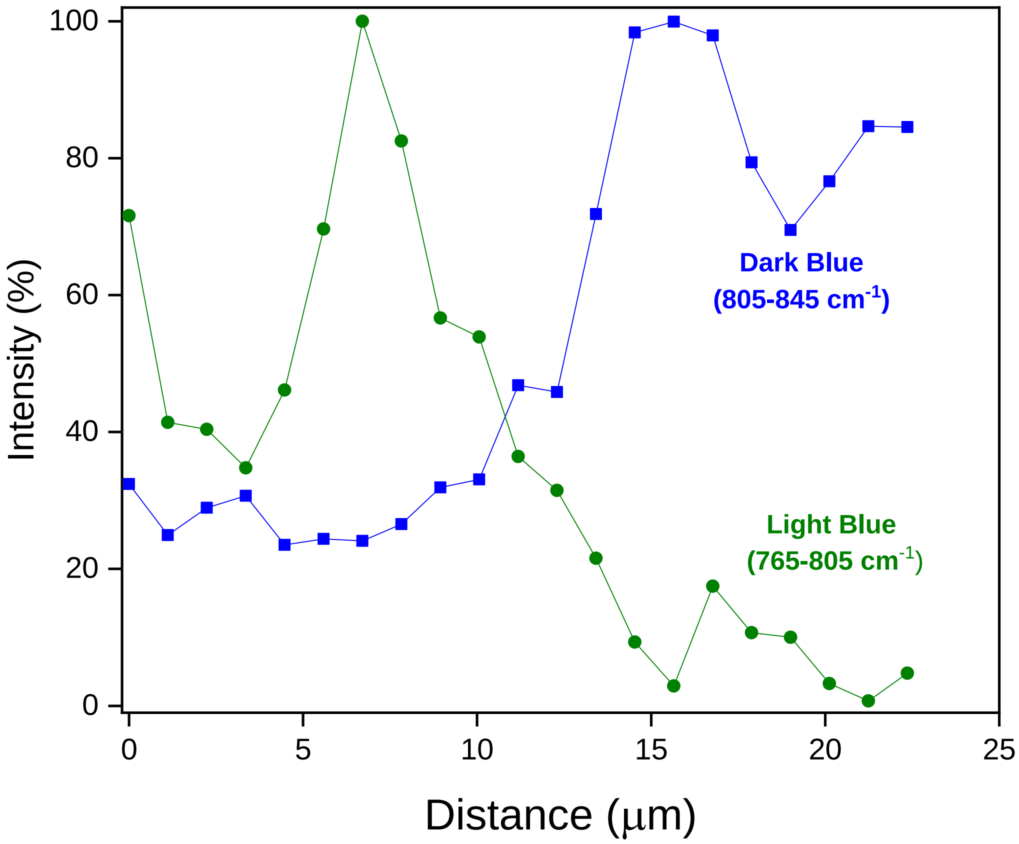

Figure S37. Raman spectra intensities of peak intensities associated with dark blue (805-845 cm^{-1}) and light blue (765-805 cm^{-1}) over the mapped section for $_{mono}$HKUST-1.

Isosteric heat of adsorption (Q_{st}) calculations.

A virial-type expression of the form below was used to fit the combined isotherm data for all the compounds at 273 and 298 K, where P is the pressure described in Pa, N is the adsorbed amount in mmol/g, T is the temperature in K, a_i and b_i are virial coefficients, and m and n are the number of coefficients used to describe the isotherms. Q_{st} is the coverage-dependent enthalpy of adsorption and R is the universal gas constant. All the related fitting curves are shown in **Figure S38-S39**.

$$\ln P = \ln N + \sum_{i=0}^{m} a_i N^i + \sum_{i=0}^{n} \binom{n}{k} b_i N^i \quad (12)$$

$$\mathrm{Q}_{st} = -R \sum_{i=0}^{m} a_i N^i \quad (13)$$

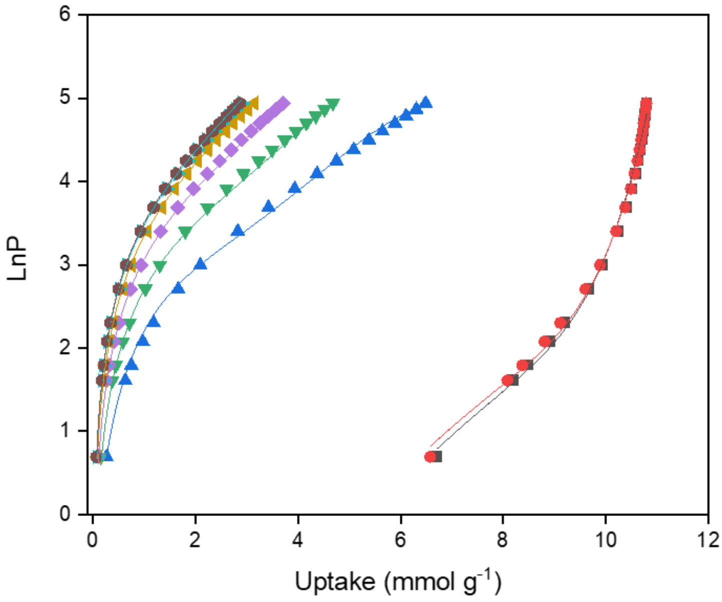

Model	VirialMethod (User)
Equation	ln(x)+1/T*(a0+a1*x+a2*x^2+a3*x^3+a4*x^4+a5*x^5)+(b0+b1*x+b2*x^2+b3*x^3)
a0*	-495.97254 ± 18.01508
a1*	144.31701 ± 27.42109
a2*	-109.59639 ± 12.81778
a3*	23.75641 ± 2.43565
a4*	-2.16152 ± 0.20504
a5*	0.07826 ± 0.00682
b0*	4.95057 ± 0.06757
b1*	-0.21895 ± 0.08795
b2*	0.25235 ± 0.03138
b3*	-0.02659 ± 0.00309
Reduced Chi-Sqr*	0.00338
R-Square (COD)	0.99379
R-Square (COD)*	0.99799
Adj. R-Square*	0.99787

Figure S38. Virial fittings for H_2 absolute (N_{abs}) isotherms collected at multiple temperatures (75.6, 77, 160, 195, 233, 273, 298 and 303 K) for $_{mono}$HKUST-1.

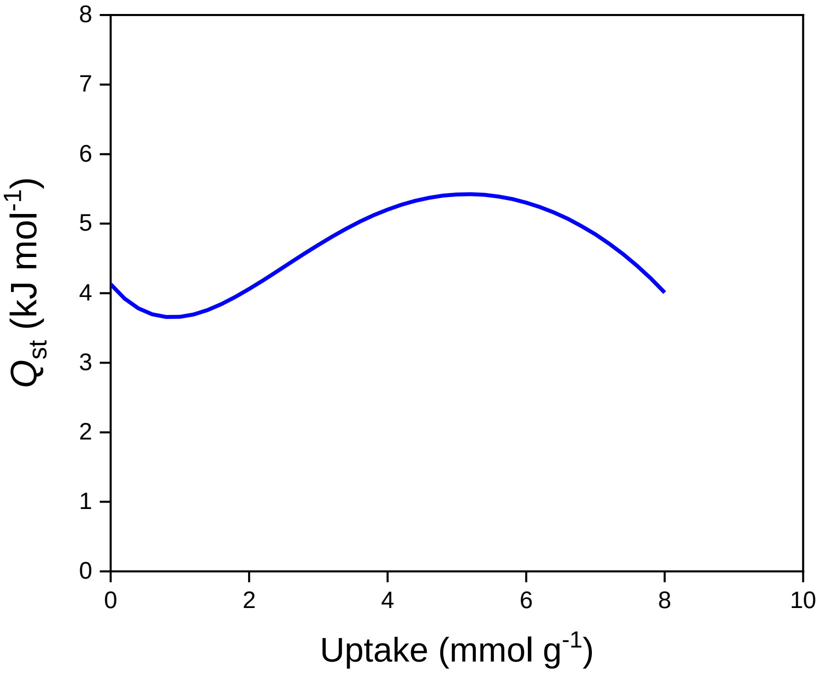

Figure S39. H_2 isosteric heat of adsorption calculated using the Virial method (Equation 12 & 13) for $_{mono}$HKUST-1.

High Pressure H_2 Adsorption Comparisons

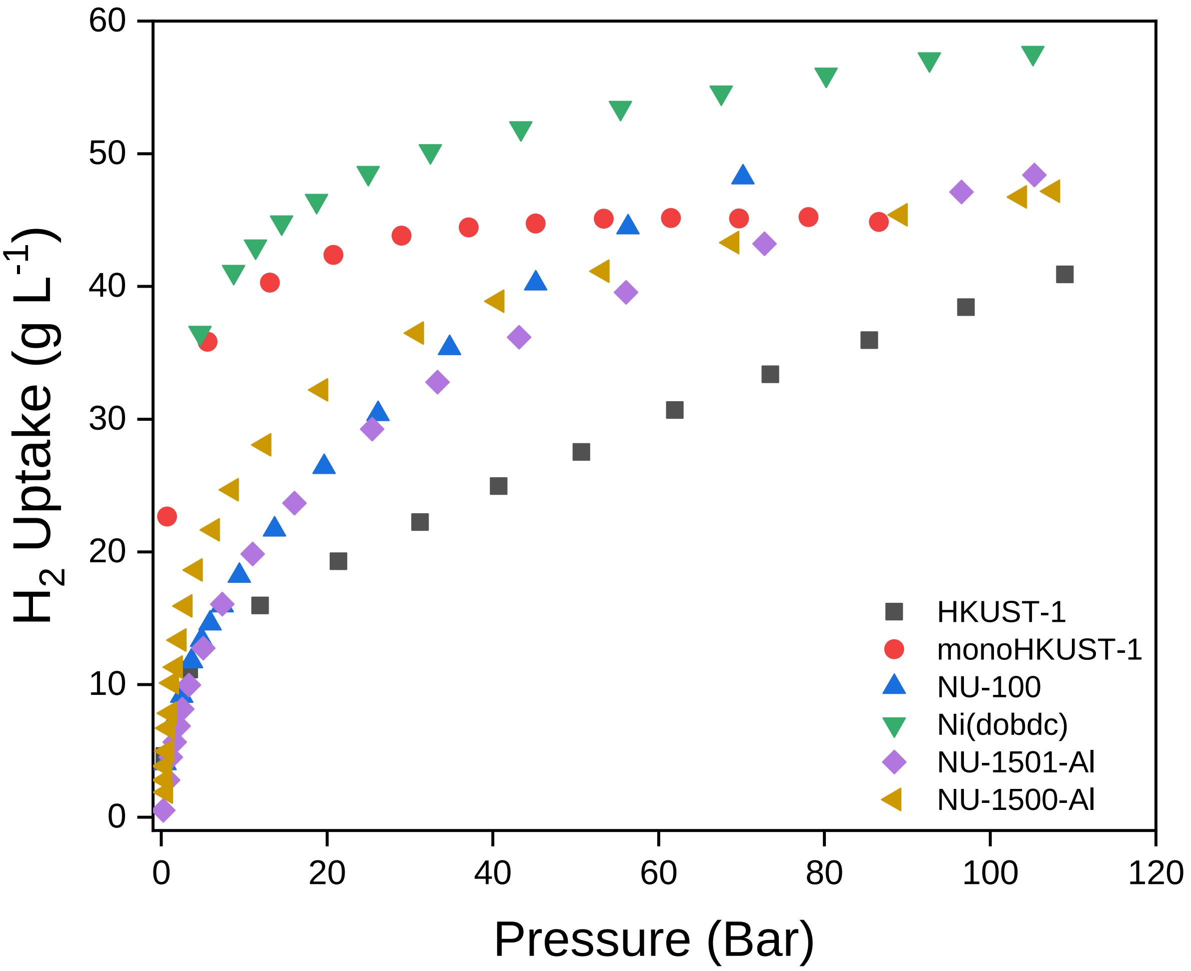

Figure S40. Absolute (N_{abs}) H_2 adsorption isotherms at 77 K for $_{mono}$HKUST-1 compared to existing benchmark MOFs for high pressure H_2 adsorption [12,27,28]. Note: All densities based on theoretical crystal densities except for HKUST-1 powder and $_{mono}$HKUST-1 which are based upon experimental packing density (0.2979 g cm^{-3}) and envelope density (1.07 g cm^{-3}), respectively.

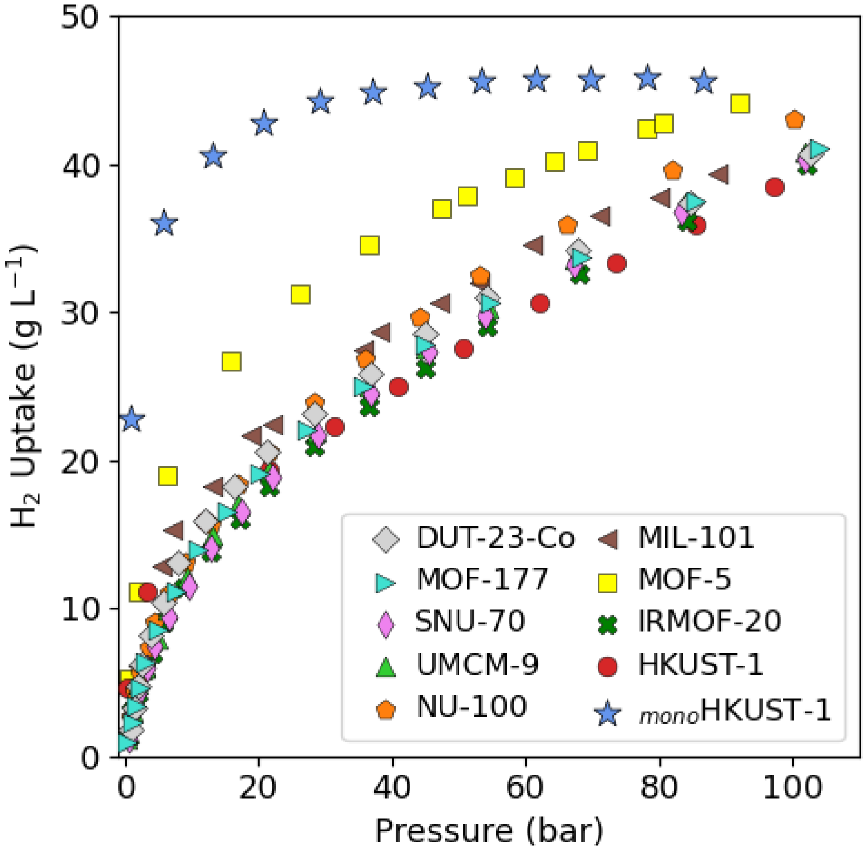

Figure S41. Absolute (N_{abs}) H_2 adsorption isotherms at 77 K for $_{mono}$HKUST-1 compared to existing benchmark MOFs for high pressure H_2 adsorption [29,30]. Note: Volumetric performance for IRMOF-20, DUT-23-Co, MOF-177, SNU-70, UMCM-9, NU-100 and MOF-5 are based on a packing density of 0.2 g cm^{-3} [29]. A packing density of 0.447 g cm^{-3} was used to estimate the volumetric H_2 uptake for MIL-101 [30,31]. HKUST-1 powder and $_{mono}$HKUST-1 volumetric performances were based upon an experimental packing density (0.2979 g cm^{-3}) and envelope density (1.07 g cm^{-3}), respectively.

Table S19. Physicochemical, textual and absolute (N_{abs}) H_2 sorption properties of benchmark MOFs.

Materials	S_{BET} $m^2\ g^{-1}$	V_{Tot} $cm^3\ g^{-1}$	ρ_{bulk} $g\ cm^{-3}$	$\rho_{crystal}$ $g\ cm^{-3}$	S_{BET} $m^2 cm^{-1}$	V_{Tot} $cm^3 cm^{-3}$	**Total H_2 Uptake at 77 K**				Ref.
							**100 bar wt. %**	**100 bar g L^{-1}**	**50 bar g L^{-1}**	**25 bar g L^{-1}**	
NU-1500-Al	3560	1.460	-	0.498	-	-	8.2	46.2[a]	38.0[a]	35.0[a]	12
NU-1501-Al	7310	2.910	-	0.283	-	-	14.5	47.9[a]	36.0[a]	29.0[a]	12
Ni(dobdc)	1321	0.560	-	1.194	-	-	4.8	57.3[a]	52.2[a]	48.6[a]	28
NU-100	6143	2.820	-	0.291	-	-	16.5	53.0[a]	42.0[a]	30.0[a]	27
NU-125	3230	1.330	-	0.578	-	-	6.7	47.0[a]	43.0[a]	39.0[a]	32
MOF-177	3450	1.600	0.390	0.430	1346	0.624	11.5	45.0[b]	37.5[b]	34.0[b]	33,34
MOF-5	2268	0.903	0.510	0.605	1157	0.461	8.1	43.0[b]	39.0[b]	31.0[b]	35,36
MIL-101	2856	1.370	0.447	0.440	1277	0.612	9.3	41.5[b]	33.0[b]	24.0[b]	30,31
IRMOF-20	4145	1.688	0.200	0.510	829	0.338	9.3	40.0[b]	27.2[b]	19.3[b]	29
DUT-23-Co	5181	2.110	0.200	0.413	1036	0.422	10.7	40.7[b]	29.9[b]	21.4[b]	29
SNU-70	4944	2.014	0.200	0.405	989	0.403	10.8	40.4[b]	28.1[b]	20.1[b]	29
UMCM-9	5039	2.053	0.200	0.370	1008	0.411	11.5	40.7[b]	28.9[b]	20.2[b]	29
NU-100	5869	2.391	0.200	0.291	1174	0.478	14.1	43.1[b]	31.4[b]	22.3[b]	29
CA-4600	2001	0.950	0.710	N/A	1420	0.675	-	-	-	43.0[b]	37
CA-4700	3771	1.750	0.460	N/A	1734	0.805	-	-	-	37.0[b]	37
AX21	2975	1.620	0.500	N/A	1487	0.810	9.1	47.0[b]	41.0[b]	37.0[b]	38
$_{mono}$HKUST-1	1552	0.634	1.064	0.883	1651	0.675	4.3	46.7[c]	45.7[c]	43.8[c]	This Work
HKUST-1	1850	1.290	0.500	0.883	925	0.645	7.1	39.0[b]	27.5[b]	20.7[b]	This Work

[a]Volumetric values (g L^{-1}) based on theoretical Crystal densities; [b]Values for volumetric adsorption based on experimental packing densities; [c] Values for volumetric adsorption based on experimental densities using Hg porosimetry.[e]measured and calculated by NREL.

Table S20. Cryogenic absolute (N_{abs}) H_2 working capacities of *mono*HKUST-1 compared to benchmark MOFs [12,13,27,28,32,39] for pressure swing and combined temperature-pressure swing delivery systems.

	100 bar 77 K → 5 bar 77 K		100 bar 77 K → 5 bar 160 K				
**MOF**	**wt. %**	**g L^{-1}**	**wt. %**	**g L^{-1}**	**Q_{st} (kJ mol^{-1})**	**$\rho_{crystal}$ (g cm^{-3})**	**Ref.**
**NU-100**	13.7	40.0^a	N/A	N/A	N/A	0.291	27
**Ni(dobdc)**	1.7	20.1^a	N/A	N/A	N/A	1.194	28
**HKUST-1**	2.0	17.0^a	5.2	46.0^a	6.5	0.879	32
**NOTT-112**	5.3	24.0^a	9.1	41.0^a	5.1	0.446	32
**NU-125**	4.1	24.0^a	8.5	49.0^a	5.1	0.578	32
**rht-MOF-7**	1.8	14.0^a	4.7	37.0^a	5.9	0.789	32
**Cu-MOF-74**	1.0	13.0^a	3.0	39.0^a	5.6	1.323	32
**PCN-250**	1.8	16.0^a	5.2	47.0^a	6.6	0.896	32
**NU-1000**	5.2	30.0^a	8.3	48.0^a	5.0	0.571	32
**UiO-67**	2.9	20.0^a	6.0	41.0^a	5.8	0.688	32
**UiO-68-Ant**	4.3	26.0^a	7.8	47.0^a	6.0	0.607	32
**CYCU-3-Al**	5.5	27.0^a	8.7	41.0^a	4.5	0.447	32
**DMOF-1**	1.6	14.0^a	4.8	42.0^a	4.9	0.873	32
**NU-1101**	6.1	30.0^a	9.1	47.0^a	5.5	0.459	39
**NU-1102**	6.9	31.0^a	9.6	44.0^a	4.5	0.403	39
**NU-1103**	10.1	33.0^a	12.6	43.0^a	3.8	0.298	39
**NU-1500-Al**	4.4	26.0^a	8.2	44.6^a	4.9	0.283	12
**NU-1501-Al**	9.0	35.3^a	14.0	46.2^a	4.0	0.283	12
**MOF-5**	4.5	31.1	7.8	51.9	N/A	0.605	13
**SNU-70**	7.8	34.3	10.6	47.9	N/A	0.405	13
**IRMOF-20**	5.7	33.4	9.1	51.0	N/A	0.510	13
***mono*HKUST-1**	2.0	21.1^b	4.0	43.3^b	4.2	1.07^c	This Work

^{a}Values based on theoretical crystal densities; ^{b}Experimental envelope density = 1.07 g cm^{-3}; ^{c}Values based on experimental densities using Hg porosimetry.

Table S21. Near ambient absolute (N_{abs}) H_2 working capacities of *mono*HKUST-1 compared to benchmark MOFs [28,40] for pressure swing and combined temperature-pressure swing delivery systems.

	100 bar 25 °C → 5 bar 25 °C	100 bar -75 °C → 5 bar -75 °C	100 bar -40 °C → 5 bar 25 °C	100 bar -75 °C → 5 bar 25 °C	100 bar -75 °C → 5 bar 100 °C	
	**g L^{-1}**	**g L^{-1}**	**g L^{-1}**	**g L^{-1}**	**g L^{-1}**	**Ref.**
***mono*HKUST-1**[a,b]	9.3	14.8	12.4	15.7	15.9	This Work
**Co_2(dobdc)**[c]	10.5	18.2	17.3	21.9	22.3	28
**Ni_2(dobdc)**[c]	11	19	18.2	23	23.4	28
**Co_2(dobdc)**[c]	8.8	16.5	14	18.3	18.6	28
**Ni_2(dobdc)**[c]	9.9	18.4	16.6	21.4	21.8	28
**MOF-5**[c]	8.8	15.8	12.8	16.5	16.7	28
**$V_2Cl_{2.8}$(btdd)**[c]	7.6	12.2	13.1	15.5	17	40

[a]Pore volume = 0.621 cm^3 g^{-1}; [b]Experimental envelope density = 1.07 g cm^{-3}; [c]Values based on theoretical crystal densities.

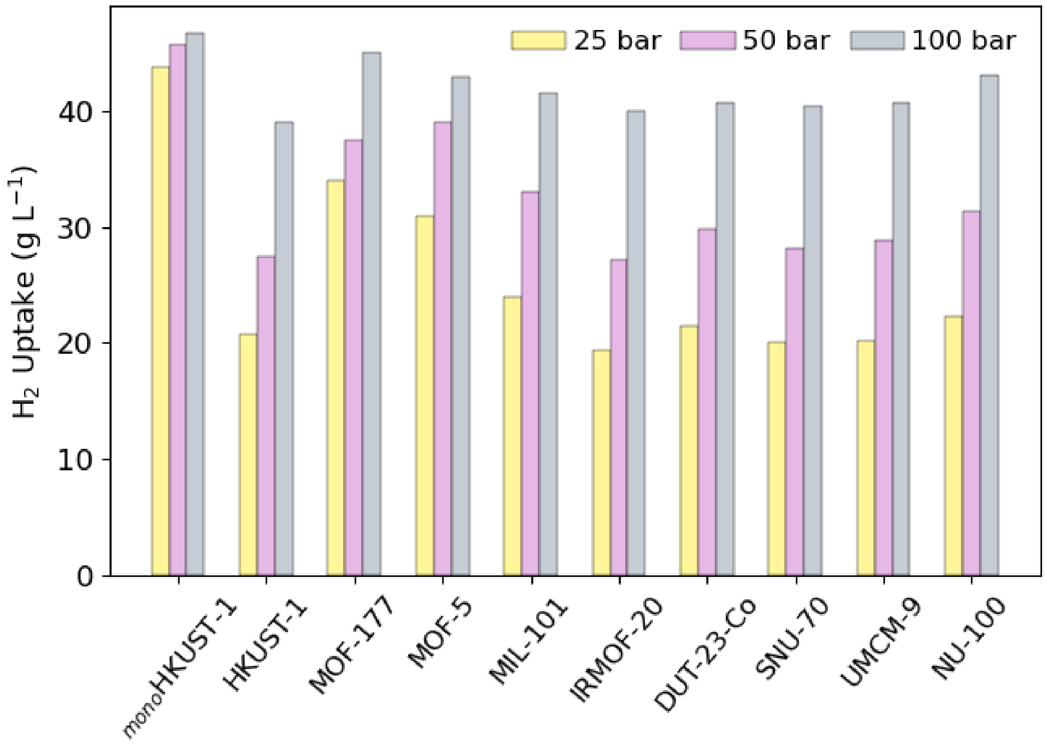

Figure S42. Comparison of the absolute (N_{abs}) H_2 storage capacity of *mono*HKUST-1 compared to existing benchmark MOFs at 25, 50 and 100 bar and 77 K. Note: Volumetric performance for IRMOF-20, DUT-23-Co, MOF-177, SNU-70, UMCM-9, NU-100 and MOF-5 are based on a packing density of 0.2 g cm^{-3} [29]. A packing density of 0.447 g cm^{-3} was used to estimate the volumetric H_2 uptake for MIL-101 [30,31]. HKUST-1 powder and *mono*HKUST-1 volumetric performances were based upon an experimental packing density (0.2979 g cm^{-3}) and envelope density (1.07 g cm^{-3}), respectively.

17

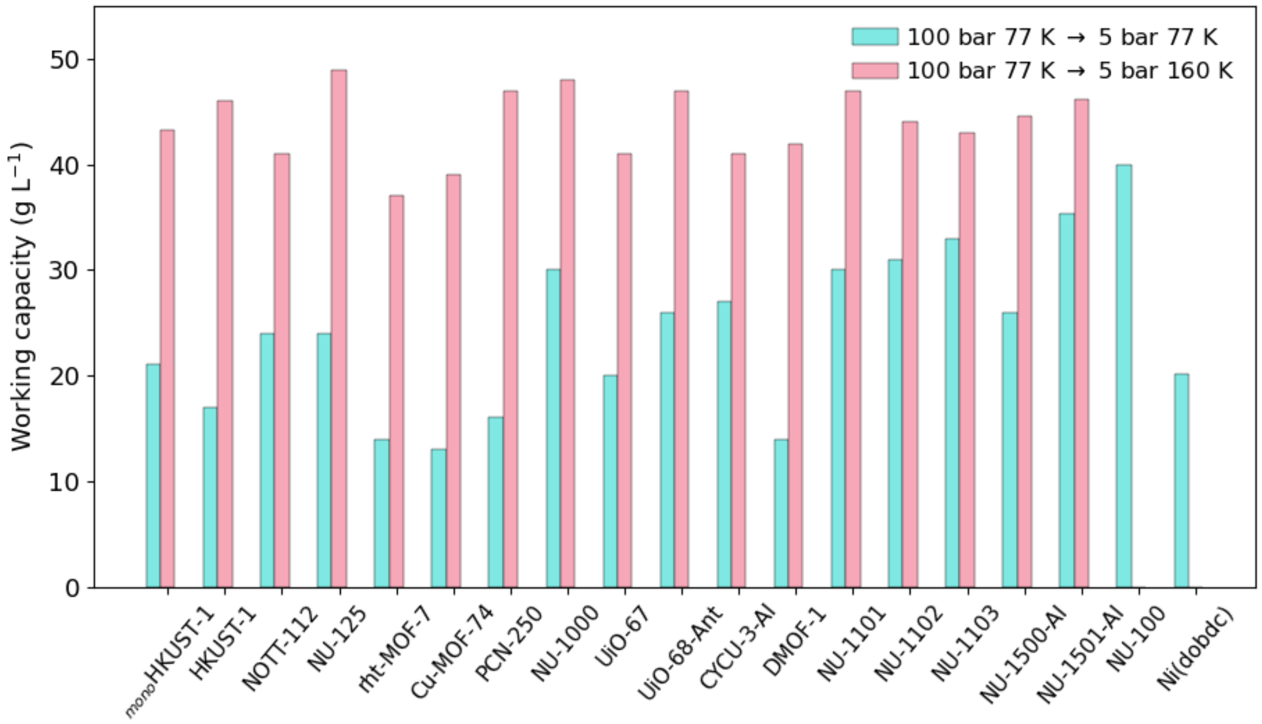

Figure S43. Comparison of the absolute (N_{abs}) H_2 working capacity of $_{mono}$HKUST-1 compared to existing benchmark MOFs for pressure swing (100 bar 77 K → 5 bar 77 K) and temperature-pressure swing (100 bar 77 K → 5 bar 160 K) delivery systems. Note: Volumetric performance for NU-100, Ni(dobdc), HKUST-1, NOTT-112, NU-125, rht-MOF-7, Cu-MOF-74, PCN-250, NU-1000, UiO-67, UiO-68-Ant, CYCU-3-Al, DMOF-1, NU-1101, NU-1102, NU-1103, NU-1500-Al and NU-1501-Al are based on reported theoretical crystal densities [12,27,28,39]. $_{mono}$HKUST-1 volumetric performance was based upon an experimental envelope density (1.07 g cm^{-3}).

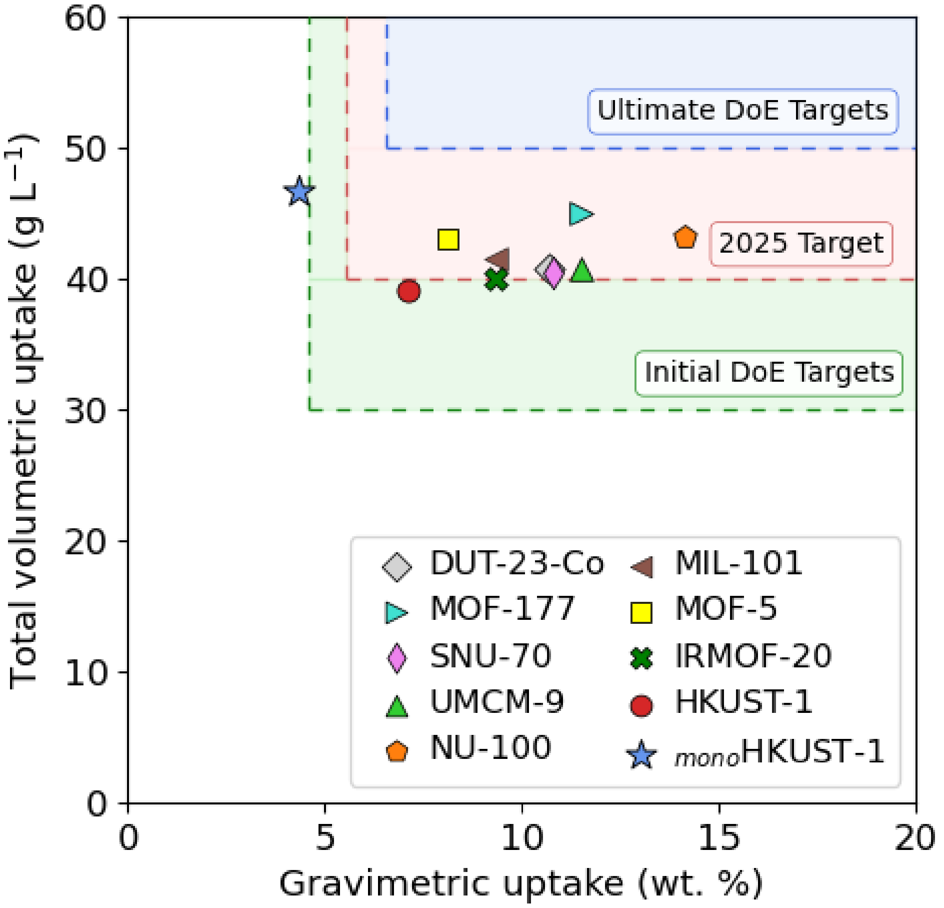

Figure S44. Comparison of the gravimetric *vs.* volumetric H_2 adsorption performance of $_{mono}$HKUST-1 compared to existing benchmark MOFs at 77 K and 100 bar. Note: Volumetric performance for IRMOF-20, DUT-23-Co, MOF-177, SNU-70, UMCM-9, NU-100 and MOF-5 are based on a packing density of 0.2 g cm^{-3} [29]. A packing density of 0.447 g cm^{-3} was used to estimate the volumetric H_2 uptake for MIL-101 [30,31]. HKUST-1 powder and $_{mono}$HKUST-1 volumetric performances were based upon an experimental packing density (0.2979 g cm^{-3}) and envelope density (1.07 g cm^{-3}), respectively.

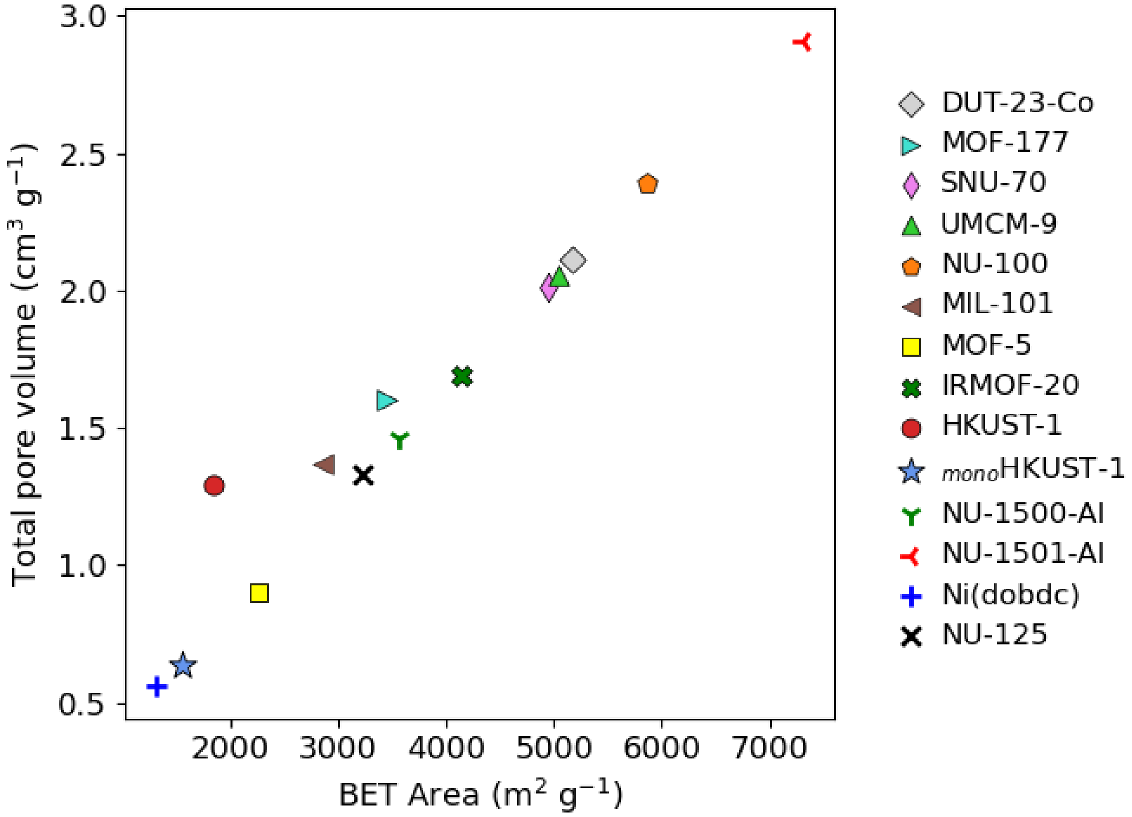

Figure S45. Comparison of the gravimetric total pore volume *vs*. gravimetric BET area of $_{mono}$HKUST-1 compared to existing benchmark MOFs [12,27–31].

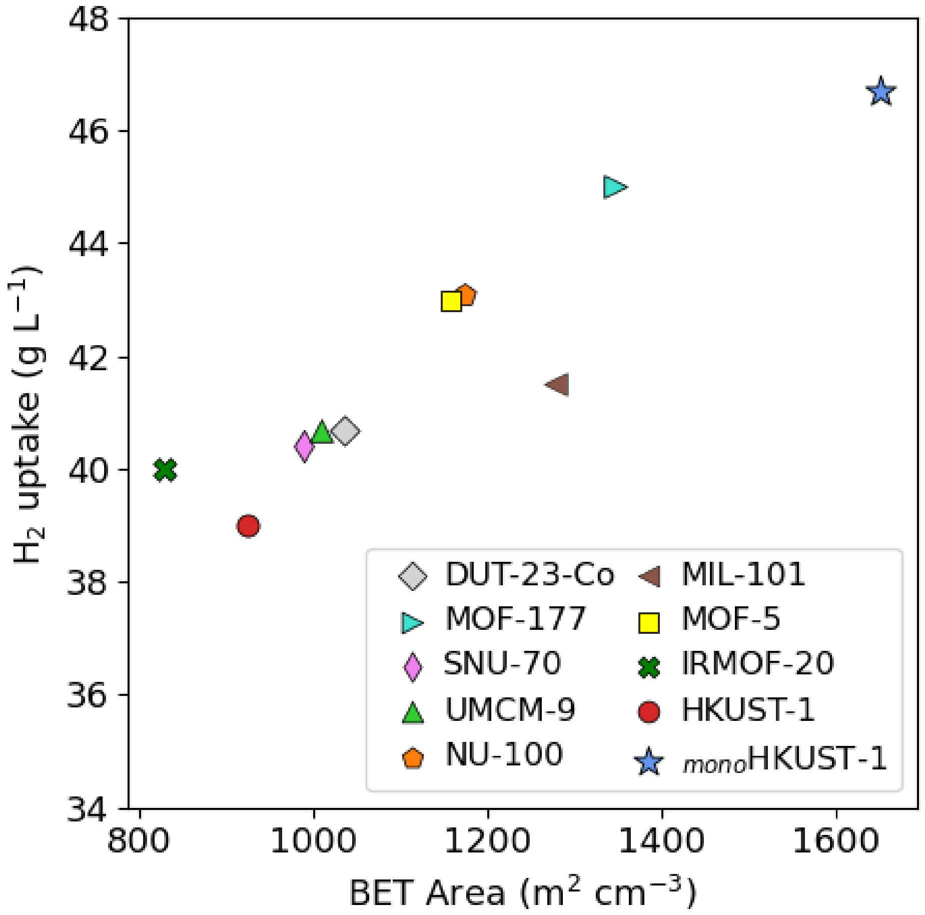

Figure S46. Comparison of the volumetric BET area vs. volumetric H_2 adsorption capacity at 100 bar and 77 K for *mono*HKUST-1 compared to existing benchmark MOFs. Note: Volumetric performance for IRMOF-20, DUT-23-Co, MOF-177, SNU-70, UMCM-9, NU-100 and MOF-5 are based on a packing density of 0.2 g cm^{-3} [29]. A packing density of 0.447 g cm^{-3} was used to estimate the volumetric H_2 uptake for MIL-101 [30,31]. Ni(dobdc) performance was calculated using the previously reported packing density of 0.366 g cm^{-3} [28]. HKUST-1 powder and *mono*HKUST-1 volumetric performances were based upon an experimental packing density (0.2979 g cm^{-3}) and envelope density (1.07 g cm^{-3}), respectively.

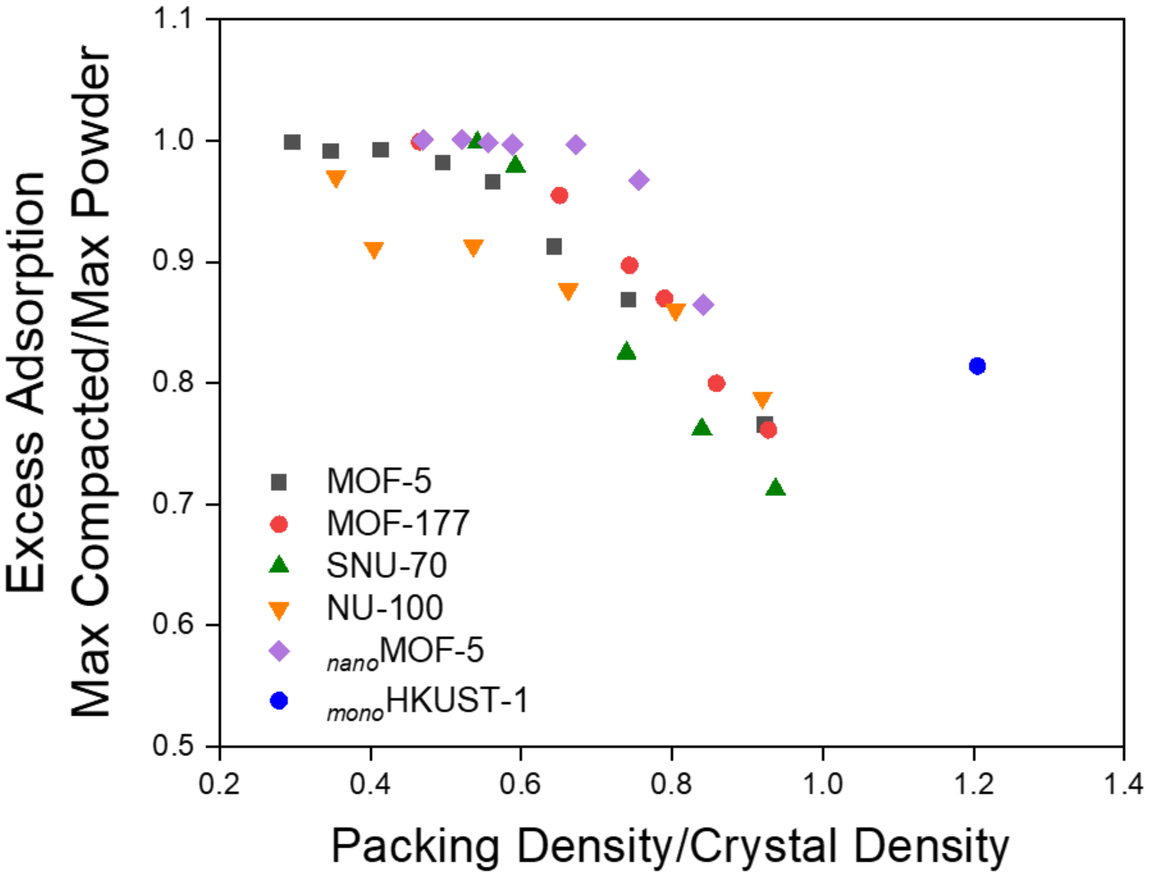

Figure S47. Comparison of the mechanical degradation of MOF *vs.* compaction density for *mono*HKUST-1 compared to existing benchmark MOFs. The y-axis corresponds to the ratio between the maximum excess adsorption at 77 K for a MOF compacted to a specific density divided by the value for the initial value measured for the powder. The x-axis corresponds to the density of the compacted MOF divided by its crystal density. Note: Values for MOF-5, MOF-177, SNU-70 and NU-100 have been adapted from Ref. [29]. *nano*MOF-5 has been adapted from Ref. [41]. *mono*HKUST-1 volumetric performances were based upon an experimental envelope density of 1.07 g cm^{-3}.

Monolith Stability Testing

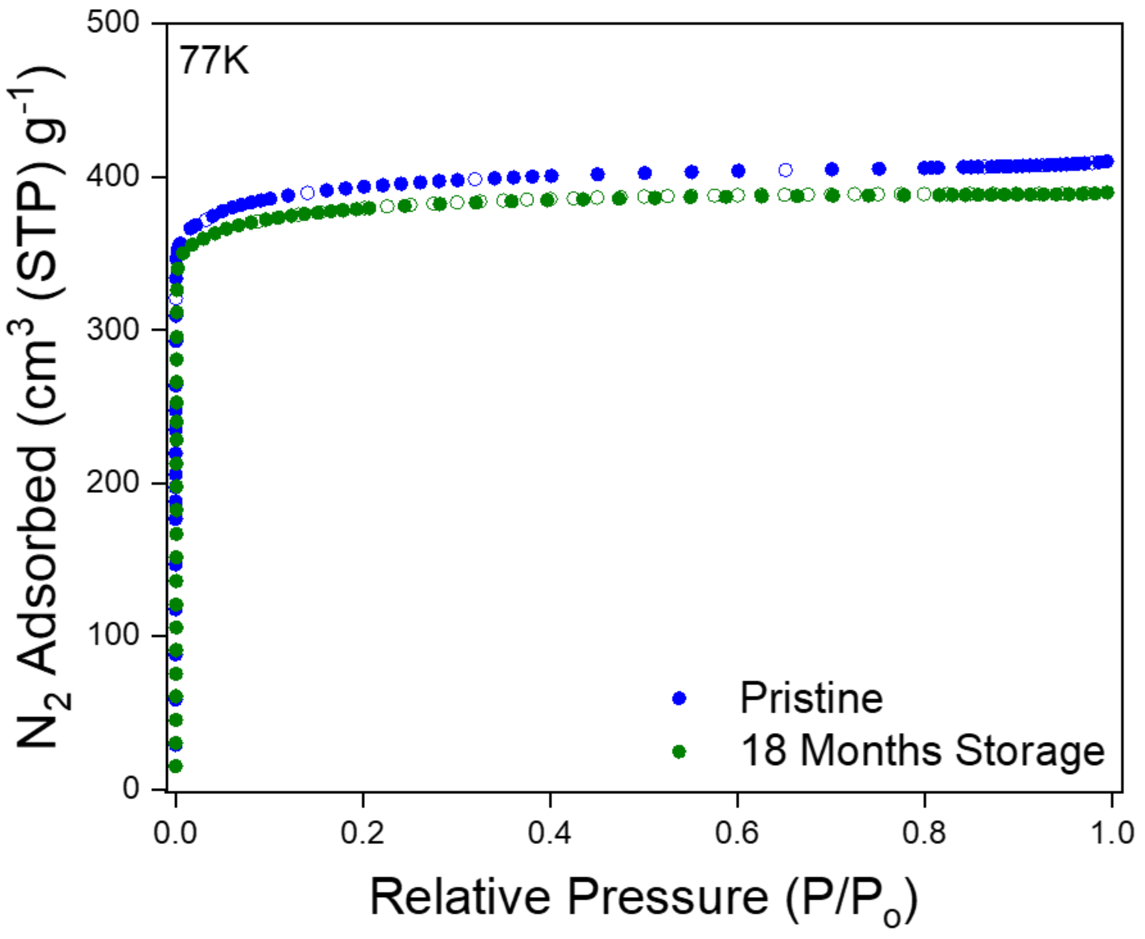

Figure S48. Linear 77 K N_2 adsorption isotherm for pristine $_{mono}$HKUST-1 and a $_{mono}$HKUST-1 sample stored at room temperature in a desiccator for 18 months. Closed circles represent adsorption whilst open circles represent desorption.

Synthesis Scale-Up Cost Reductions

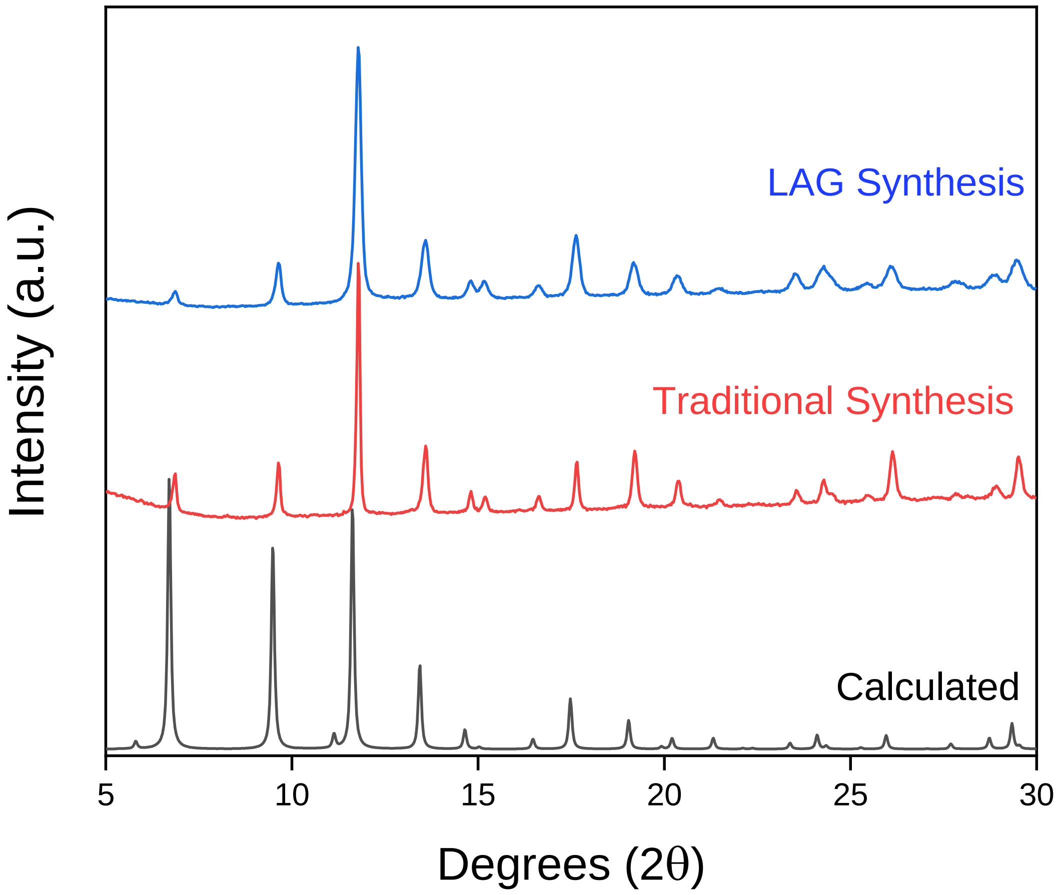

Figure S49. Powder X-ray diffraction patterns (PXRD) for $_{mono}$HKUST-1 synthesised *via* traditional approach and synthesised using liquid assisted grinding (LAG) HKUST-1 primary particles compared to calculated PXRD.

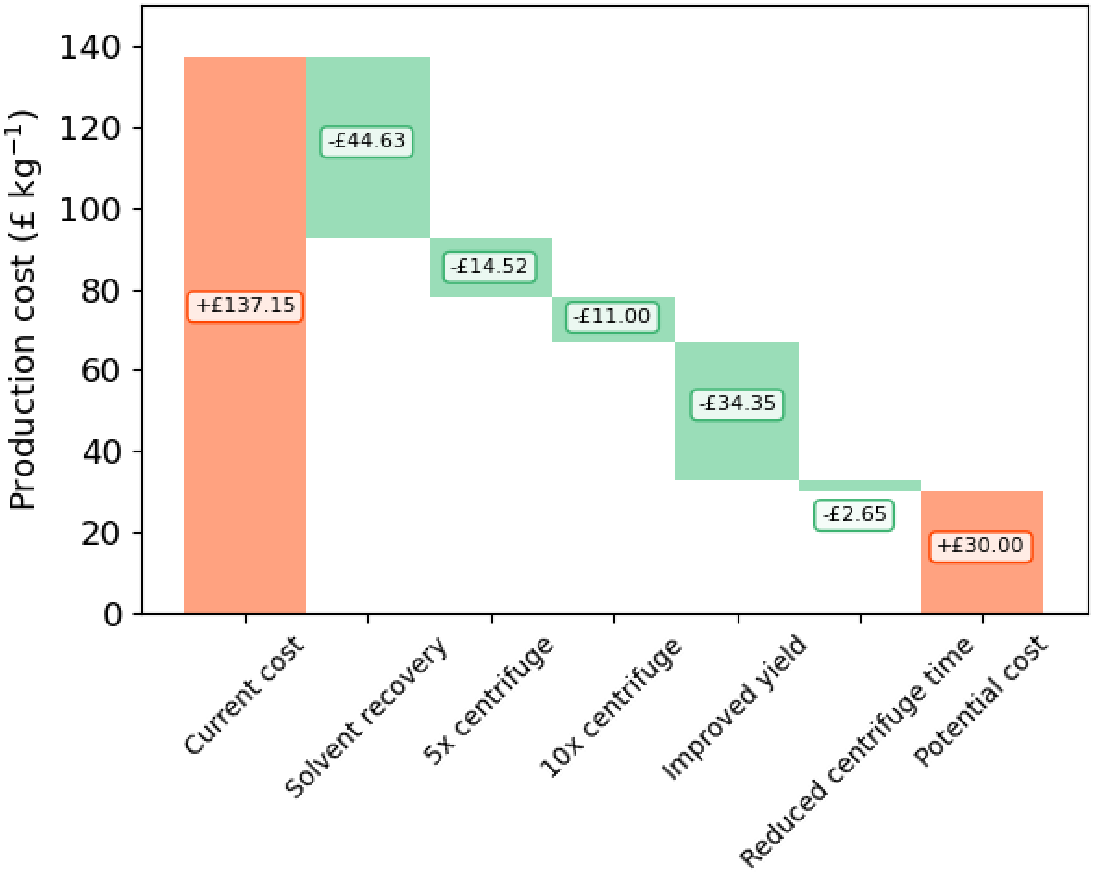

Figure S50. Waterfall charts for $_{mono}$HKUST-1 showing the potential costs of utilizing solvent recovery, improved yield and reduced cycling times to lower overall MOF production costs.

References

(1) Tian, T.; Zeng, Z.; Vulpe, D.; Casco, M. E.; Divitini, G.; Midgley, P. A.; Silvestre-Albero, J.; Tan, J. C.; Moghadam, P. Z.; Fairen-Jimenez, D. A Sol-Gel Monolithic Metal-Organic Framework with Enhanced Methane Uptake. *Nat. Mater.* **2018**, *17* (2), 174–179. https://doi.org/10.1038/NMAT5050.

(2) Nazarian, D.; Camp, J. S.; Sholl, D. S. A Comprehensive Set of High-Quality Point Charges for Simulations of Metal-Organic Frameworks. *Chem. Mater.* **2016**, *28* (3), 785–793. https://doi.org/10.1021/acs.chemmater.5b03836.

(3) Dubbeldam, D.; Calero, S.; Ellis, D. E.; Snurr, R. Q. RASPA: Molecular Simulation Software for Adsorption and Diffusion in Flexible Nanoporous Materials. *Mol. Simul.* **2016**, *42* (2), 81–101. https://doi.org/10.1080/08927022.2015.1010082.

(4) Potoff, J. J.; Siepmann, J. I. Vapor-Liquid Equilibria of Mixtures Containing Alkanes, Carbon Dioxide, and Nitrogen. *AIChE J.* **2001**, *47* (7), 1676–1682. https://doi.org/10.1002/aic.690470719.

(5) Bucior, B. J.; Bobbitt, N. S.; Islamoglu, T.; Goswami, S.; Gopalan, A.; Yildirim, T.; Farha, O. K.; Bagheri, N.; Snurr, R. Q. Energy-Based Descriptors to Rapidly Predict Hydrogen Storage in Metal-Organic Frameworks. *Mol. Syst. Des. Eng.* **2019**, *4* (1), 162–174. https://doi.org/10.1039/c8me00050f.

(6) Liu, J.; Culp, J. T.; Natesakhawat, S.; Bockrath, B. C.; Zande, B.; Sankar, S. G.; Garberoglio, G.; Johnson, J. K. Experimental and Theoretical Studies of Gas Adsorption in Cu 3(BTC) 2: An Effective Activation Procedure. *J. Phys. Chem. C* **2007**, *111* (26), 9305–9313. https://doi.org/10.1021/jp071449i.

(7) Manousakis, E. *Quantum Mechanics and Path Integrals*; Courier Corporation, 2015. https://doi.org/10.1093/acprof:oso/9780198749349.003.0013.

(8) Mayo, S. L.; Olafson, B. D.; Goddard, W. A. DREIDING: A Generic Force Field for Molecular Simulations. *J. Phys. Chem.* **1990**, *94* (26), 8897–8909. https://doi.org/10.1021/j100389a010.

(9) Rappé, A. K.; Casewit, C. J.; Colwell, K. S.; Goddard, W. A.; Skiff, W. M. UFF, a Full Periodic Table Force Field for Molecular Mechanics and Molecular Dynamics Simulations. *J. Am. Chem. Soc.* **1992**, *114* (25), 10024–10035. https://doi.org/10.1021/ja00051a040.

(10) Wilmer, C. E.; Kim, K. C.; Snurr, R. Q. An Extended Charge Equilibration Method. *J. Phys. Chem. Lett.* **2012**, *3* (17), 2506–2511. https://doi.org/10.1021/jz3008485.

(11) Sarkisov, L.; Harrison, A. Computational Structure Characterisation Tools in Application to Ordered and Disordered Porous Materials. *Mol. Simul.* **2011**, *37* (15), 1248–1257. https://doi.org/10.1080/08927022.2011.592832.

(12) Chen, Z.; Li, P.; Anderson, R.; Wang, X.; Zhang, X.; Robison, L.; Redfern, L. R.; Moribe, S.; Islamoglu, T.; Gómez-Gualdrón, D. A.; et al. Balancing Volumetric and Gravimetric Uptake in Highly Porous Materials for Clean Energy. *Science (80-.).* **2020**, *368* (6488), 297–303. https://doi.org/10.1126/science.aaz8881.

(13) Ahmed, A.; Seth, S.; Purewal, J.; Wong-Foy, A. G.; Veenstra, M.; Matzger, A. J.; Siegel, D. J. Exceptional Hydrogen Storage Achieved by Screening Nearly Half a Million Metal-Organic Frameworks. *Nat. Commun.* **2019**, *10* (1). https://doi.org/10.1038/s41467-019-09365-w.

(14) Michels, A.; de Graaff, W.; Ten Seldam, C. A. Virial Coefficients of Hydrogen and Deuterium at Temperatures between -175°C and +150°C. Conclusions from the Second Virial Coefficient with Regards to the Intermolecular Potential. *Physica* **1960**, *26* (6), 393–408. https://doi.org/10.1016/0031-8914(60)90029-X.

(15) Hu, N.; Sun, X.; Hsu, A. Monte Carlo Simulations of Hydrogen Adsorption in Alkali-Doped Single-Walled Carbon Nanotubes. *J. Chem. Phys.* **2005**, *123* (4), 4981–4984. https://doi.org/10.1063/1.1954727.

(16) Sarkisov, L.; Bueno-Perez, R.; Sutharson, M.; Fairen-Jimenez, D. Materials Informatics with PoreBlazer v4.0 and the CSD MOF Database. *Chem. Mater.* **2020**, *32* (23), 9849–9867.

https://doi.org/10.1021/acs.chemmater.0c03575.

(17) Hurst, K. E.; Parilla, P. A.; O'Neill, K. J.; Gennett, T. An International Multi-Laboratory Investigation of Carbon-Based Hydrogen Sorbent Materials. *Appl. Phys. A Mater. Sci. Process.* **2016**, *122* (1), 1–9. https://doi.org/10.1007/s00339-015-9537-x.

(18) Lemmon, E. W.; McLinden, M. O.; Friend, D. G. Thermophysical Properties of Fluid Systems. NIST Chemistry WebBook, NIST Standard Reference Database Number 69, National Institute of Standards and Technology, Gaithersburg MD, 20899. 2005.

(19) Parilla, P. A.; Gross, K.; Hurst, K.; Gennett, T. Recommended Volumetric Capacity Definitions and Protocols for Accurate, Standardized and Unambiguous Metrics for Hydrogen Storage Materials. *Appl. Phys. A Mater. Sci. Process.* **2016**, *122* (3), 1–18. https://doi.org/10.1007/s00339-016-9654-1.

(20) Fairen-Jimenez, D.; Colón, Y. J.; Farha, O. K.; Bae, Y. S.; Hupp, J. T.; Snurr, R. Q. Understanding Excess Uptake Maxima for Hydrogen Adsorption Isotherms in Frameworks with Rht Topology. *Chem. Commun.* **2012**, *48* (85), 10496–10498. https://doi.org/10.1039/c2cc35711a.

(21) Micromeritics' Particle Testing Authority https://www.particletesting.com/.

(22) Dawson, D. M.; Jamieson, L. E.; Mohideen, M. I. H.; McKinlay, A. C.; Smellie, I. A.; Cadou, R.; Keddie, N. S.; Morris, R. E.; Ashbrook, S. E. High-Resolution Solid-State 13C NMR Spectroscopy of the Paramagnetic Metal-Organic Frameworks, STAM-1 and HKUST-1. *Phys. Chem. Chem. Phys.* **2013**, *15* (3), 919–929. https://doi.org/10.1039/c2cp43445h.

(23) Chupas, P. J.; Chapman, K. W.; Lee, P. L. Applications of an Amorphous Silicon-Based Area Detector for High-Resolution, High-Sensitivity and Fast Time-Resolved Pair Distribution Function Measurements. *J. Appl. Crystallogr.* **2007**, *40* (3), 463–470. https://doi.org/10.1107/S0021889807007856.

(24) Toby, B. H.; Von Dreele, R. B. GSAS-II: The Genesis of a Modern Open-Source All Purpose Crystallography Software Package. *J. Appl. Crystallogr.* **2013**, *46* (2), 544–549. https://doi.org/10.1107/S0021889813003531.

(25) Yang, X.; Juhas, P.; Farrow, C. L.; Billinge, S. J. L. XPDFsuite: An End-to-End Software Solution for High Throughput Pair Distribution Function Transformation, Visualization and Analysis. *arXiv Prepr. arXiv1402.3163* **2014**.

(26) O'Nolan, D.; Huang, G.; Kamm, G. E.; Grenier, A.; Liu, C. H.; Todd, P. K.; Wustrow, A.; Tran, G. T.; Montiel, D.; Neilson, J. R.; et al. A Thermal-Gradient Approach to Variabletemperature Measurements Resolved in Space. *J. Appl. Crystallogr.* **2020**, *53* (3), 662–670. https://doi.org/10.1107/S160057672000415X.

(27) Farha, O. K.; Yazaydin, A. Ö.; Eryazici, I.; Malliakas, C. D.; Hauser, B. G.; Kanatzidis, M. G.; Nguyen, S. T.; Snurr, R. Q.; Hupp, J. T. De Novo Synthesis of a Metal-Organic Framework Material Featuring Ultrahigh Surface Area and Gas Storage Capacities. *Nat. Chem.* **2010**, *2* (11), 944–948. https://doi.org/10.1038/nchem.834.

(28) Kapelewski, M. T.; Runčevski, T.; Tarver, J. D.; Jiang, H. Z. H.; Hurst, K. E.; Parilla, P. A.; Ayala, A.; Gennett, T.; Fitzgerald, S. A.; Brown, C. M.; et al. Record High Hydrogen Storage Capacity in the Metal-Organic Framework Ni2(m-Dobdc) at Near-Ambient Temperatures. *Chem. Mater.* **2018**, *30* (22), 8179–8189. https://doi.org/10.1021/acs.chemmater.8b03276.

(29) Purewal, J.; Veenstra, M.; Tamburello, D.; Ahmed, A.; Matzger, A. J.; Wong-Foy, A. G.; Seth, S.; Liu, Y.; Siegel, D. J. Estimation of System-Level Hydrogen Storage for Metal-Organic Frameworks with High Volumetric Storage Density. *Int. J. Hydrogen Energy* **2019**, *44* (29), 15135–15145. https://doi.org/10.1016/j.ijhydene.2019.04.082.

(30) Blanita, G.; Coldea, I.; Misan, I.; Lupu, D. Hydrogen Cryo-Adsorption by Hexagonal Prism Monoliths of MIL-101. *Int. J. Hydrogen Energy* **2014**, *39* (30), 17040–17046. https://doi.org/10.1016/j.ijhydene.2014.08.038.

(31) Ardelean, O.; Blanita, G.; Borodi, G.; Lazar, M. D.; Misan, I.; Coldea, I.; Lupu, D. Volumetric Hydrogen Adsorption Capacity of Densified MIL-101 Monoliths. *Int. J. Hydrogen Energy* **2013**, *38* (17), 7046–7055. https://doi.org/10.1016/j.ijhydene.2013.03.161.

(32) García-Holley, P.; Schweitzer, B.; Islamoglu, T.; Liu, Y.; Lin, L.; Rodriguez, S.; Weston, M. H.; Hupp, J. T.; Gómez-Gualdrón, D. A.; Yildirim, T.; et al. Benchmark Study of Hydrogen Storage in Metal-Organic Frameworks under Temperature and Pressure Swing Conditions. *ACS Energy Lett.* **2018**, *3* (3), 748–754. https://doi.org/10.1021/acsenergylett.8b00154.

(33) Zacharia, R.; Cossement, D.; Lafi, L.; Chahine, R. Volumetric Hydrogen Sorption Capacity of Monoliths Prepared by Mechanical Densification of MOF-177. *J. Mater. Chem.* **2010**, *20* (11), 2145–2151. https://doi.org/10.1039/b922991d.

(34) Dailly, A.; Poirier, E. Evaluation of an Industrial Pilot Scale Densified MOF-177 Adsorbent as an on-Board Hydrogen Storage Medium. *Energy Environ. Sci.* **2011**, *4* (9), 3527–3534. https://doi.org/10.1039/c1ee01426a.

(35) Purewal, J. J.; Liu, D.; Yang, J.; Sudik, A.; Siegel, D. J.; Maurer, S.; Müller, U. Increased Volumetric Hydrogen Uptake of MOF-5 by Powder Densification. *Int. J. Hydrogen Energy* **2012**, *37* (3), 2723–2727. https://doi.org/10.1016/j.ijhydene.2011.03.002.

(36) Purewal, J.; Liu, D.; Sudik, A.; Veenstra, M.; Yang, J.; Maurer, S.; Müller, U.; Siegel, D. J. Improved Hydrogen Storage and Thermal Conductivity in High-Density MOF-5 Composites. *J. Phys. Chem. C* **2012**, *116* (38), 20199–20212. https://doi.org/10.1021/jp305524f.

(37) Blankenship, T. S.; Balahmar, N.; Mokaya, R. Oxygen-Rich Microporous Carbons with Exceptional Hydrogen Storage Capacity. *Nat. Commun.* **2017**, *8* (1), 1545. https://doi.org/10.1038/s41467-017-01633-x.

(38) Tian, M.; Rochat, S.; Polak-Kraśna, K.; Holyfield, L. T.; Burrows, A. D.; Bowen, C. R.; Mays, T. J. Nanoporous Polymer-Based Composites for Enhanced Hydrogen Storage. *Adsorption* **2019**, *25* (4), 889–901. https://doi.org/10.1007/s10450-019-00065-x.

(39) Gómez-Gualdrón, D. A.; Wang, T. C.; García-Holley, P.; Sawelewa, R. M.; Argueta, E.; Snurr, R. Q.; Hupp, J. T.; Yildirim, T.; Farha, O. K. Understanding Volumetric and Gravimetric Hydrogen Adsorption Trade-off in Metal-Organic Frameworks. *ACS Appl. Mater. Interfaces* **2017**, *9* (39), 33419–33428. https://doi.org/10.1021/acsami.7b01190.

(40) Jaramillo, D. E.; Jiang, H. Z. H.; Evans, H. A.; Chakraborty, R.; Furukawa, H.; Brown, C. M.; Head-Gordon, M.; Long, J. R. Ambient-Temperature Hydrogen Storage via Vanadium(II)-Dihydrogen Complexation in a Metal-Organic Framework. *J. Am. Chem. Soc.* **2021**, *143* (16), 6248–6256. https://doi.org/10.1021/jacs.1c01883.

(41) Suresh, K.; Aulakh, D.; Purewal, J.; Siegel, D. J.; Veenstra, M.; Matzger, A. J. Optimizing Hydrogen Storage in MOFs through Engineering of Crystal Morphology and Control of Crystal Size. *J. Am. Chem. Soc.* **2021**, *143* (28), 10727–10734. https://doi.org/10.1021/jacs.1c04926.
